# Miro1-mediated mitochondrial transfer boosts stem cell therapy for muscle atrophy via restoring mitochondrial homeostasis

**DOI:** 10.1186/s13287-026-05191-2

**Published:** 2026-07-20

**Authors:** Fangling Zhao, Jingxin Fu, Linli Gao, Mengting Zhou, Yuhui Xia, Zexian Zhao, MengNa Yang, Jiahui He, Wei Zhu, Mengxiong Pan, Kaiming Liu

**Affiliations:** 1 Department of Neurology, The Second Affiliated Hospital, Zhejiang University School of Medicine, No.88 Jiefang Road, Hangzhou, Zhejiang China; 2 Department of Cardiology, Provincial Key Lab of Cardiovascular Research, Second Affiliated Hospital, Zhejiang University School of Medicine, Hangzhou, Zhejiang China; 3 Department of Neurology, Zhejiang Hospital, Zhejiang University School of Medicine, No.1229 Gudun Road, Hangzhou, China; 4 Department of Neurology, Hangzhou Normal University Affiliated Hospital, Hangzhou, Zhejiang China; 5 College of Life and Environmental Sciences, Hangzhou Normal University, Hangzhou, Zhejiang China; 6 Department of Neurology, Hangzhou Hospital of Traditional Chinese Medicine, Zhejiang Chinese Medicine University, No.453 Tiyuchang Road, Hangzhou, Zhejiang China; 7 Department of Neurology, The First People’s Hospital of Huzhou, First Affiliated Hospital of Huzhou University, Huzhou, China

**Keywords:** Muscle atrophy, Miro1, Stem cells, Mitochondrial transfer, Mitochondrial homeostasis

## Abstract

Mitochondrial dysfunction underlies the major defect in muscle atrophy (characterized by the loss of skeletal muscle mass and function). Mesenchymal stem cells (MSCs), which can mediate mitochondrial transfer (MT) via tunneling nanotubes (TNTs), have been shown to exert therapeutic effects, yet the underlying mechanism remains unclear. Mitochondrial Rho GTPase 1 (Miro1) is crucial for regulating mitochondrial homeostasis; in this study, we aimed to investigate the roles of Miro1 and Milton in MSC-based therapy for muscle atrophy. Dexamethasone (DEX)-induced C2C12 cells and chronically aged mice were used as in vitro cellular and in vivo muscle atrophy models, respectively. In vitro experiments demonstrated that overexpression of Milton alone failed to enhance MT in DEX-induced C2C12 cells. Although Milton could promote the formation of TNTs, it was unable to drive mitochondrial movement along microtubules in the absence of Miro1.In contrast, Miro1 knockdown (MSCmiro1^Lo^) significantly reduced MT in vivo, while Miro1 overexpression (MSCmiro1^Hi^) improved mitochondrial morphology, increased muscle fiber count and cross-sectional area, upregulated the expression of type I/III collagen, and downregulated the expression of Atrogin1 and MURF1. Additionally, Miro1 overexpression ameliorated functional outcomes such as grip strength, running distance, and physical activity, and elevated the levels of proteins related to mitochondrial fusion, mitophagy, and biogenesis in damaged muscle cells. These findings indicate that Miro1 is a critical driver of MT, and Miro1-enhanced MT confers substantial in vivo therapeutic benefits. This study provides robust evidence supporting Miro1 as a potential target for the treatment of muscle atrophy-related disorders.

**Supplementary Information:**

The online version contains supplementary material available at https://doi.org/10.1186/s13287-026-05191-2.

## Introduction

Muscle atrophy refers to a pathophysiological state resulting from various causes, characterized by reduced volume of skeletal muscle tissue (predominantly striated muscle), thinning of muscle fibers, or a decrease in their number—accompanied by diminished muscle strength and impaired function. Its pathological mechanisms involve three core pathways: inadequate energy supply, oxidative stress-mediated damage, and enhanced cellular apoptosis. Furthermore, it engages in a vicious cycle with mitochondrial dysfunction: muscle atrophy exacerbates mitochondrial impairment, and conversely, mitochondrial dysfunction further drives muscle atrophy [1]. Myofibers, highly dependent on mitochondrial energy production, require strict mitochondrial homeostasis mediated by mitochondrial quality control (MQC) pathways: biogenesis, dynamics (fission/fusion), mitophagy, and proteostasis. When mitochondrial homeostasis is disrupted, dysfunctional mitochondria have impaired oxidative phosphorylation and reduced biosynthesis; reactive oxygen species (ROS) production increases and accumulates, activating the ubiquitin-proteasome system and accelerating muscle atrophy [2, 3].

The mitochondrial outer membrane protein mitochondrial Rho GTPase 1 (Miro1) acts as a calcium-dependent sensor, regulates mitochondrial transport and distribution along microtubules [4], affects mitochondrial morphology [5] and ultrastructure [6], and regulates MQC mechanisms such as mitophagy [7], thereby affecting mitochondrial function and cellular physiology. Miro1 is commonly expressed in humans, but its expression level may be related to the mitochondrial density and energy requirements of specific cell types, and studies have shown that it is highly expressed in cardiac and skeletal muscle tissues [8]. However, the relationship between Miro1 and mitochondrial function in muscle atrophy remains unknown.

While exercise is the most practical strategy for alleviating muscle atrophy, its application is limited by patient mobility [9], stem cell transplantation with regenerative and repair properties is emerging as an attractive therapy. MSCs have been shown to improve muscle atrophy by exerting anti-inflammatory effects, promoting the activation of endogenous satellite cells for muscle regeneration, reducing apoptosis, and enhancing mitochondrial biogenesis [10–12]. Notably, there is growing evidence that MSCs can mediate MT into different cell types, including endothelial cells, cardiomyocytes, and epithelial cells via TNTs, thereby improving mitochondrial function and metabolic status in recipient cells [13–15], however, their ability to transfer mitochondria to damaged myocytes remains unclear. Interestingly, Miro1 was shown to influence the therapeutic efficacy of MSCs by modulating the efficiency of MT from MSCs to pathologic tracheal epithelial cells [16], neural stem cells [17], and neurons [18].

Since restoration of mitochondrial function is the key to the treatment of muscle atrophy, our study aimed to explore the role of Miro1 in mitochondrial dysfunction in muscle atrophy and its feasibility as a therapeutic target, to clarify the mitochondrial donation capacity of MSCs harboring mitochondria with normal expression of Miro1 to damaged muscle cells, and the salvage effect of MSCs on the mitochondrial function and MQC mechanism in muscle atrophy. In this study, we applied dexamethasone (DEX)-induced C2C12 and chronological aging mice to successfully construct an in vitro and an in vivo model of muscle atrophy and observed the presence of myotube atrophy and mitochondrial dysfunction associated with Miro1 under-expression. Despite failed rescue by direct Miro1 overexpression in muscle atrophy models, MSCs transferred functional mitochondria via TNTs, restoring MQC and muscle homeostasis. Miro1 overexpression in MSCs enhanced therapeutic efficacy, while knockdown or TNTs inhibition attenuated benefits. This study reveals Miro1 as a key regulator of MSC-mediated mitochondrial repair in muscle atrophy.

## Methods and materials

Please refer to the supplementary information for details. And the work has been reported in line with the ARRIVE guidelines 2.0.

## Results

### Mitochondrial dysfunction in muscle atrophy models is accompanied by downregulation of Miro1

**Fig. 1:**
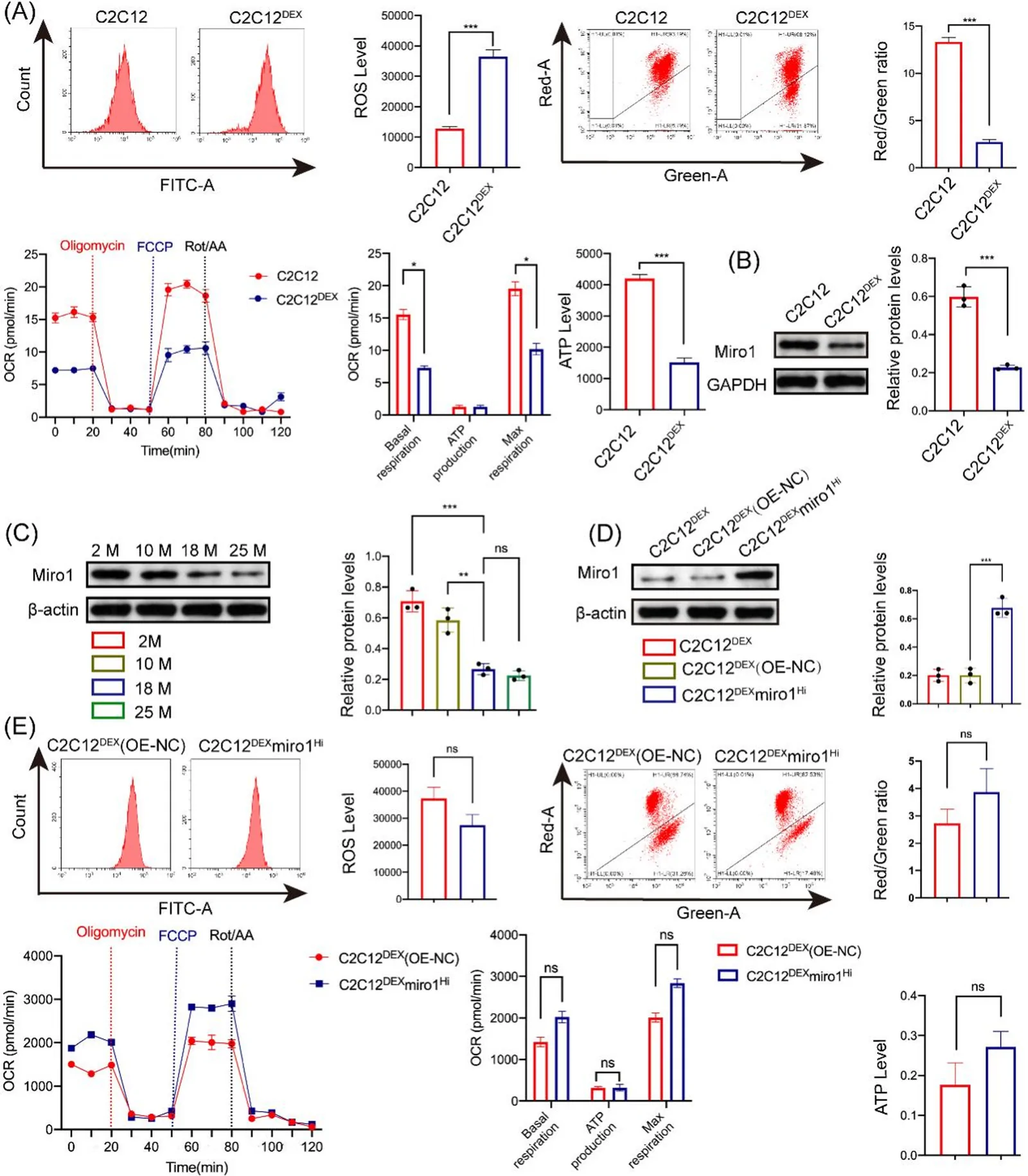
Mitochondrial dysfunction in muscle atrophy models is accompanied by downregulation of Miro1. **A** Flow cytometric analysis and histogram of ROS and MMP levels in C2C12 or C2C12^DEX^. The OCR of C2C12 or C2C12^DEX^ was measured over time (in minutes), and the basal mitochondrial OCR, ATP production, and maximal respiration of C2C12 or C2C12^DEX^ were calculated. Histogram showing the ATP levels in C2C12 or C2C12^DEX^ *n* = 6. **B** Western blot analysis of Miro1 expression levels in C2C12 and C2C12^DEX^. **C** Western blot analysis of Miro1 expression levels in GC of C57BL/6J mice of different ages. **D** Protein expression of Miro1 in C2C12^DEX^ transduced with either a lipofectamine3000 vector encoding Miro1 (C2C12 ^DEX^miro1^Hi^) or an empty vector (C2C12^DEX^(OE-NC)) as a control was evaluated. **E** Mitochondrial function indicators (ROS, MMP, OCR, and ATP levels) were measured in C2C12^DEX^miro1^Hi^ or C2C12 ^DEX^(OE-NC). Data are means ± SD. (*P < 0.05 by one-way ANOVA, **P < 0.01, ***P < 0.001, ****P < 0.0001 by using one-way ANOVA followed by Tukey’s test)

Aging is closely associated with and often regarded as the main cause of primary muscle atrophy, while secondary muscle atrophy is related to various other factors, such as glucocorticoids that can independently cause skeletal muscle atrophy by reducing protein synthesis and increasing protein degradation. Based on previous studies showing that muscle atrophy is often seen in the aging process, we therefore selected 25-month-old C57BL/6J male mice as an in vivo model of primary muscle atrophy, where mitochondrial dysfunction was also observed in skeletal muscle [19]. First, we used 1 µM DEX-induced differentiated mouse C2C12myotubes as an in vitro cell culture model of secondary muscle atrophy (C2C12^DEX^) [20] and observed that C2C12^DEX^ exhibited significantly higher ROS (*P* < 0.0001), lower mitochondrial membrane potential (MMP) (*P* < 0.0001), oxygen consumption rate (OCR) (*P* < 0.05), and ATP levels (*P* < 0.0001) compared to C2C12 (Fig. 1A), suggesting the presence of an impaired mitochondrial respiratory chain and oxidative stress in this cell culture muscle atrophy model. We then measured the expression level of the mitochondrial outer membrane protein Miro1 and demonstrated that C2C12^DEX^ exhibited lower Miro1 expression than control C2C12 (*P* < 0.001) (Fig. 1B). Importantly, the expression of Miro1 in Gastrocnemius (GC)decreased significantly as mice aged from 2 months (2 M) to 25 months (25 M) (*P* < 0.01) (Fig. 1C). These findings suggest that mitochondrial dysfunction in muscle atrophy may be related to the downregulation of Miro1 expression. To test whether exogenous Miro1 could rescue mitochondrial dysfunction, the C2C12^DEX^ cells were transduced with either lipofectamine 3000 vector encoding Miro1 (C2C12 ^DEX^miro1^Hi^) or an empty vector (C2C12^DEX^(OE-NC)) as a control. Although Miro1 expression was significantly elevated in C2C12 ^DEX^miro1^Hi^ at both the mRNA and protein levels (*P* < 0.001) (Fig. 1D) Full-length blots are presented in Supplementary Figure S7, there was no significant improvement in ROS, MMP, OCR, or ATP levels (*P*>0.05) (Fig. 1E). These results suggest that while mitochondrial dysfunction in muscle atrophy is accompanied by downregulation of Miro1, overexpression of Miro1 in C2C12^DEX^ alone did not restore mitochondrial function, indicating that mitochondrial damage in muscle atrophy outstrips the rescuing capacity of direct Miro1 overexpression. Only when the processes of mitochondrial biogenesis, fusion, and fission are restored to normal — facilitated by “normal mitochondrial replenishment” — can the energy metabolism of cells be effectively repaired. Mitochondria, after all, are the “energy factories” of cells; their normalized function, in turn, alleviates disease symptoms and lays a critical physiological foundation for treatment.

### Miro1 is critical for MSC-mediated MT and myotube rescue

**Fig. 2:**
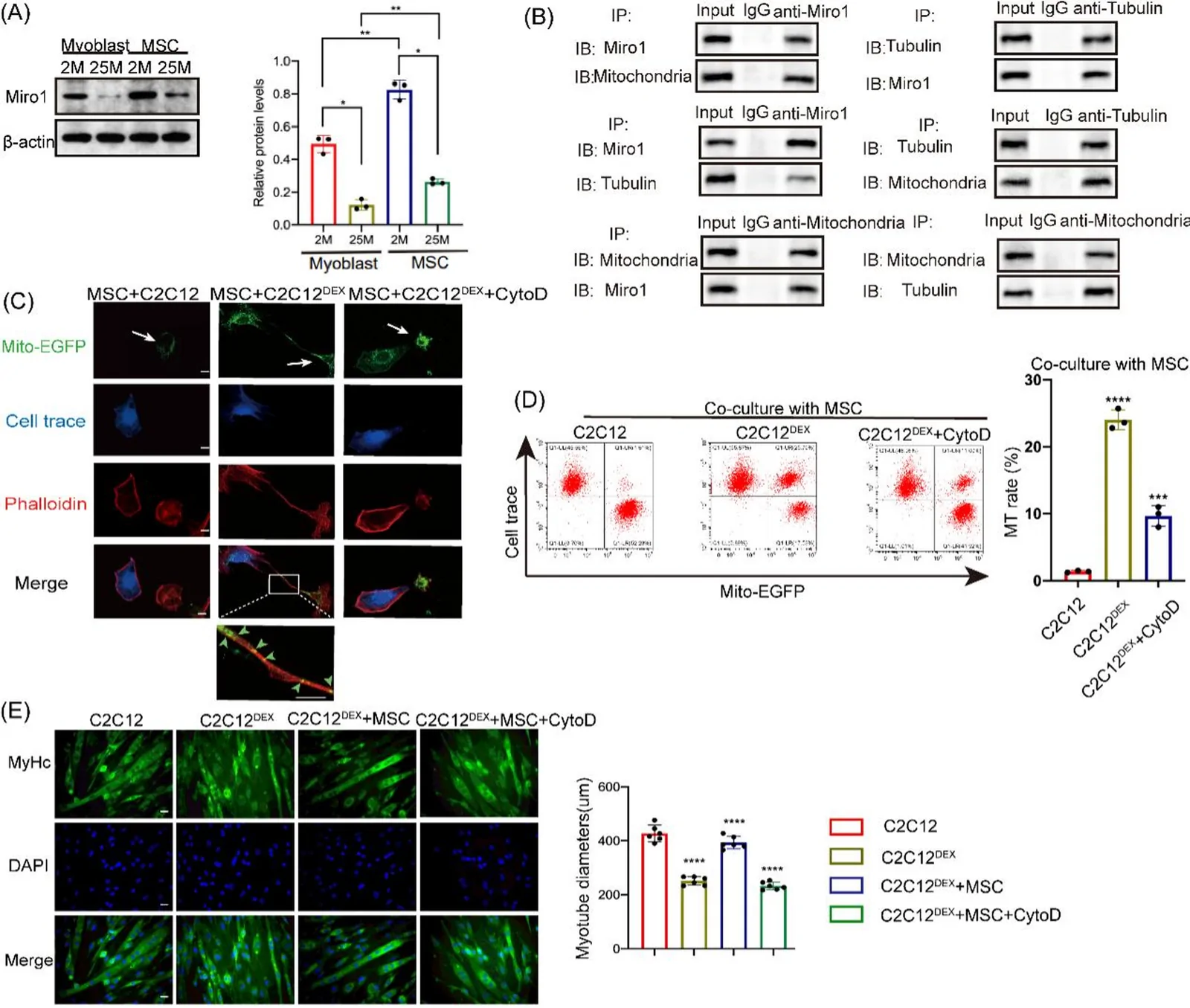
MSCs transfer mitochondria to damaged myotubes via TNTs for therapeutic effect. **A** Protein expression of Miro1 in MSCs and myoblasts isolated from 2 M and 25 M mice. (Myoblasts from 2 M mice versus from 25 M mice; MSCs from 2 M mice versus from 25 M mice; myoblasts from 2 M mice versus MSCs from 2 M; myoblasts from 25 M mice versus MSCs from 25M. **B** Co-IP assay detecting physical interactions among Miro1, mitochondrial outer membrane marker VDAC1, and α-tubulin in MSCs. VDAC1 serves as canonical mitochondrial reference protein in this experiment. **C** Confocal observation of TNT formation and MT in MSCs co-culture system with C2C12 or C2C12^DEX^. Mito-EGFP-labeled MSCs (green), CellTrace-labeled C2C12 or C2C12^DEX^ (blue), and Phalloidin-labeled TNTs (red); white arrowheads indicate MSC, and green arrowheads indicate Mito-EGFP signaling in TNTs. Scale bar, 20 μm. **D** FACS analysis of the rate of cells with double-positive signals in a co-culture system of Mito-EGFP-labeled MSCs with CellTrace-labeled C2C12 or C2C12^DEX^ to suggest mitochondrial transfer rates. **E** Immunofluorescence staining of MyH7 in C2C12, untreated C2C12^DEX^, and C2C12^DEX^ after co-culture with MSC or MSC + CytoD. Average diameter was quantified by measuring the maximum width of individual myotubes using Image J software (*n* ≥ 100 myotubes per group, 10 random fields per sample). *n* = 6, Scale bar: 50 μm, 20 μm. Data are means ± SD. (*P < 0.05, **P < 0.01, ***P < 0.001, ****P < 0.0001 by two-sided unpaired Student’s t-test; nonsignificant differences not shown)

To investigate whether MSCs could serve as a mitochondrial donor supplementing Miro1, we first compared the expression of Miro1 in primary myoblasts versus MSCs in mice, and the results showed that Miro1 levels were higher in MSCs than in myoblasts in both young and aged mice (*P* < 0.05) (Fig. 2A) Full-length blots are presented in Supplementary Figure S7. Moreover, we performed Co-IP combined with Western blotting to verify physical interactions among Miro1, the outer mitochondrial marker VDAC1, and α-tubulin in MSCs (Fig. 2B). Full-length blots are presented in Supplementary Figure S8. To visually verify whether MSCs could transfer mitochondria into myotubes via TNTs, MSCs transduced with Mito-EGFP lentivirus were co-cultured with CellTrace-labeled C2C12 or C2C12^DEX^ at a 1:1 ratio for 24 h. Immunofluorescence showed phalloidin-labeled TNTs and transported mitochondria in the C2C12^DEX^ group but not in C2C12. Application of (CytoD (500 nM), an inhibitor of actin polymerization) almost completely inhibited TNT formation (Fig. 2C) without affecting MSC viability (data not shown). FACS analysis revealed that EGFP signals in myotubes were significantly elevated (*P* < 0.0001) when MSCs were co-cultured with C2C12^DEX^ as compared to C2C12 and were reduced after the application of CytoD (*P* < 0.001) (Fig. 2D), indicating that MSCs transfer mitochondria via TNTs into impaired myotubes. Additionally, MSC co-culture ameliorated DEX-induced myotube atrophy (*P* < 0.0001) (Fig. 2E). Thus, our data confirm previous reports that the therapeutic effects offered by MSCs are related to MT through TNTs.

**Fig. 3:**
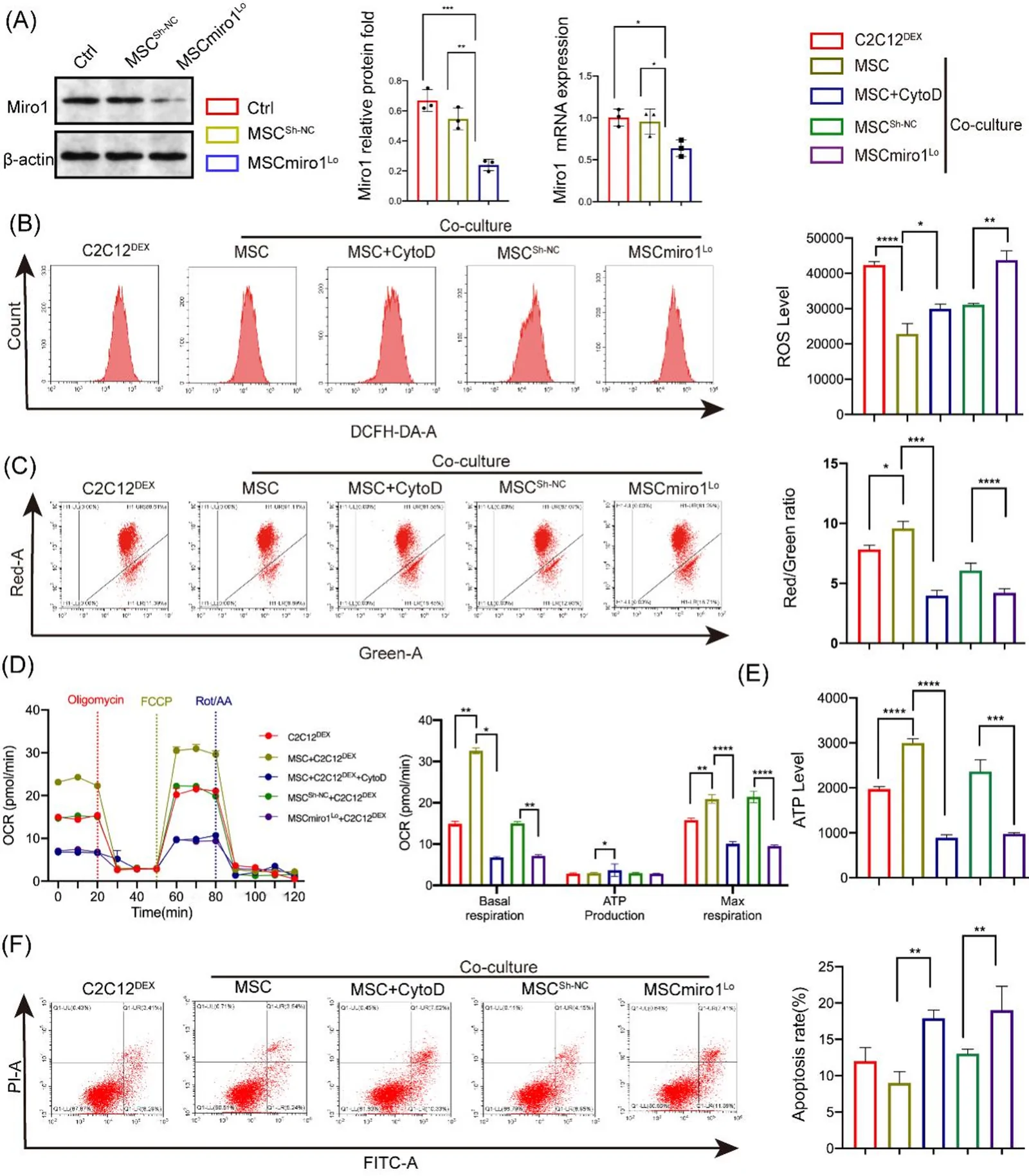
Miro1 is required in MSC-mediated restoration of mitochondrial function. **A** Protein and mRNA expression of Miro1 in MSCs transfected with Miro1-targeting shRNA (MSCmiro1^Lo^) or an empty vector (MSC^sh−NC^) as a control was evaluated. **B** Flow cytometric analysis and histogram of ROS levels in untreated C2C12^DEX^ or those sorted after co-culture with MSC, MSC + CytoD, MSC^sh−NC^, and MSCmiro1^Lo^, respectively. (Untreated C2C12^DEX^ compared with those after MSC co-culture; C2C12^DEX^ after MSC co-culture compared with those after MSC + CytoD co-culture; C2C12^DEX^ after MSC^sh−NC^ co-culture compared with those after MSCmiro1^Lo^ co-culture) (*n* = 6). **C** Flow cytometric analysis and histogram of MMP levels in C2C12^DEX^ under five different treatment conditions (as in B) (*n* = 6). **D** The OCR of C2C12^DEX^ under five different treatment conditions (as in B) was measured over time (in minutes), and the basal mitochondrial OCR, ATP production, and maximal respiration of those were calculated. **E** Histogram depicting the ATP levels in C2C12^DEX^ under five different treatment conditions (as in B). **F** Flow cytometric examination and histogram of the apoptosis rates in C2C12^DEX^ under five different treatment conditions (as in B). Data are means ± SD. (*P < 0.05, **P < 0.01, ***P < 0.001, ****P < 0.0001 by using one-way ANOVA followed by Tukey’s test; nonsignificant differences not shown)

To further investigate the role of Miro1 in MSC therapy, MSCs were then divided into two groups: those transfected with Miro1-targeting short hairpin RNA (shRNA), designated MSCmiro1^Lo^, to achieve lower Miro1 expression (*P* < 0.05), and those transfected with scrambled RNA, designated MSC^sh−NC^ (Fig. 3A) Full-length blots are presented in Supplementary Figure S9. We measured mitochondrial function and apoptosis levels in untreated C2C12^DEX^ or C2C12^DEX^ cells sorted after co-culture with MSC, MSC་CytoD, MSC^sh−NC^, and MSCmiro1^Lo^, respectively. MSC treatment reduced ROS (*P* < 0.0001), increased MMP (*P* < 0.05), OCR (*P* < 0.05), and ATP levels (*P* < 0.0001) in C2C12^DEX^ (Fig. 3B–E). In contrast, these therapeutic effects were abolished when using MSCmiro1^Lo^ or CytoD (*P* < 0.05), indicating that Miro1 is essential for TNT-mediated MT and subsequent restoration of mitochondrial function in impaired myotubes.

### The role of Miro1 in the therapeutic effects of MSCs on skeletal muscle in aging mice

**Fig. 4:**
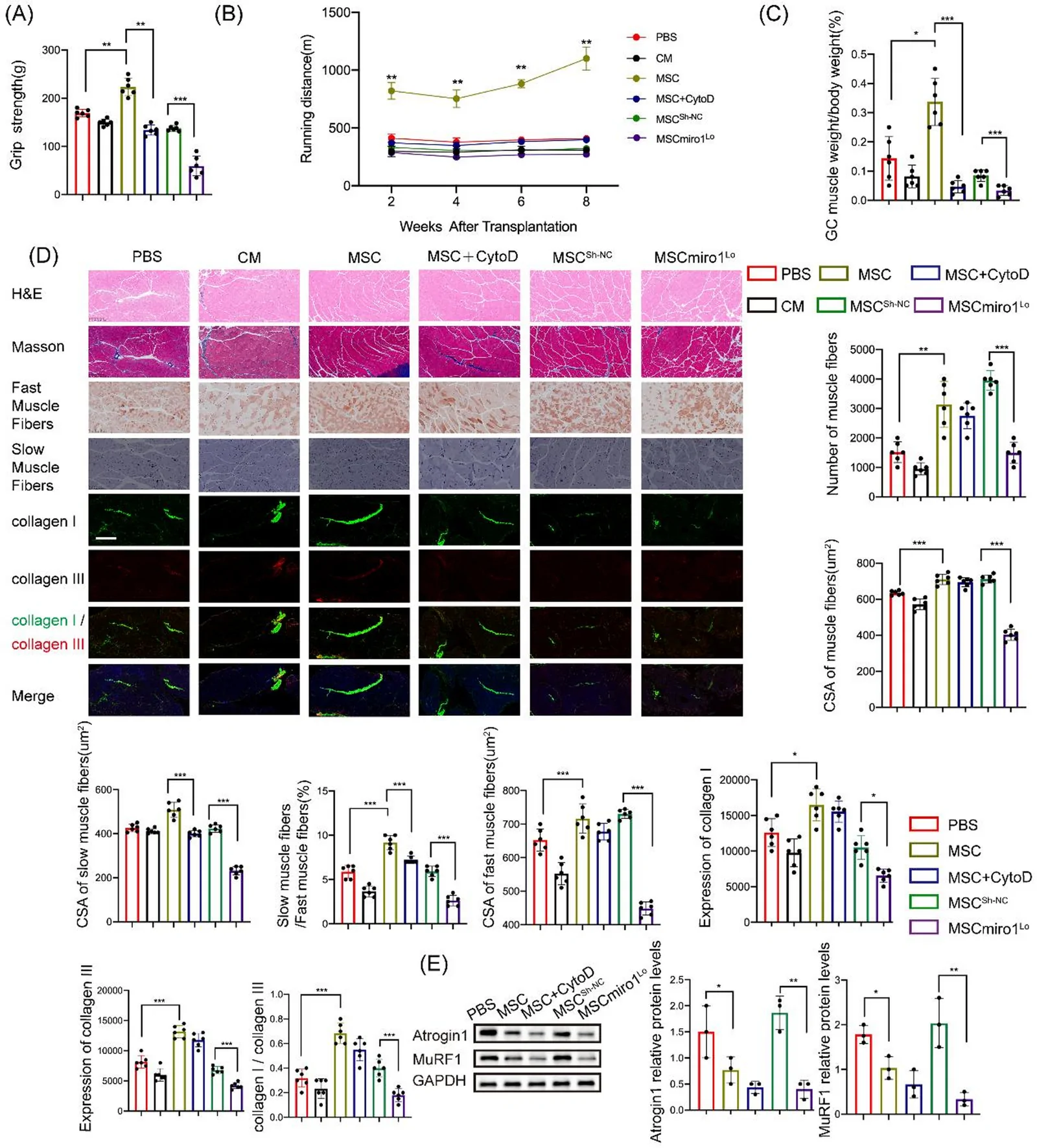
Miro1-mediated MSC therapy improves muscle function and mass in muscle atrophy mice. **A** Hindlimb grip strength of mice after four courses of PBS, CM, MSC, MSC + CytoD, MSC^sh−NC^, and MSCmiro1^Lo^ injections, respectively (PBS compared with CM, compared with MSC; MSC compared with MSC + CytoD; MSC^sh−NC^ compared with MSCmiro1^Lo^) (*n* = 6). **B** Running distance of five groups of mice (as in A) assessed via treadmill endurance test of mice (*n* = 6). **C** GC weight to body weight ratio in five groups of mice (as in A) (*n* = 6). **D** H&E staining, Masson staining (scale bar, 250 μm), and immunohistochemical staining (scale bar, 250 μm) of GC fibers from five groups of mice (as in A). Measure and count the CSA of myofibers, fast and slow fibers, and the slow-to-fast fiber ratio (*n* = 6). Immunofluorescence expression of collagen I and collagen III from five groups of mice (as in A). (The ratio of collagen I to collagen III in fast-twitch muscle-dominated muscles) collagen III(slow-twitch muscle-dominated muscles) (scale bar, 250 μm) (*n* = 6) (**E**) (Protein expression levels of Atrogin1 and MuRF1 in GC from five groups of mice (PBS, MSC, MSC + CytoD, MSC^sh−NC^, and MSCmiro1^Lo^ injections, respectively (PBS compared with MSC; MSC compared with MSC + CytoD; MSC^sh−NC^ compared with MSCmiro1^Lo^)). Data are means ± SD. (*P < 0.05, **P < 0.01, ***P < 0.001, ****P < 0.0001 by two-sided unpaired Student’s t-test; nonsignificant differences not shown)

**Fig. 5:**
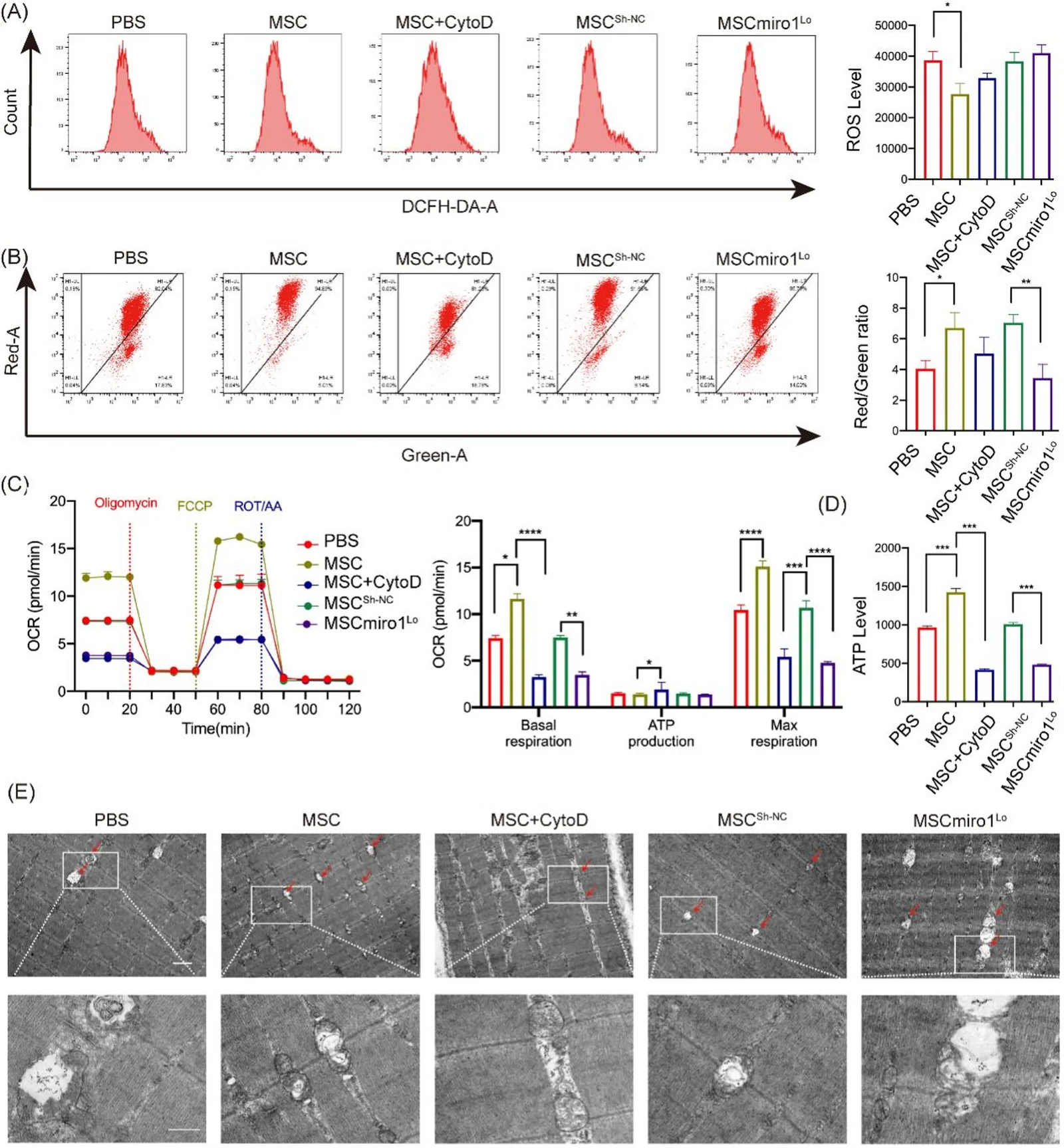
Miro1-mediated MSC therapy improves mitochondrial function and structure in muscle atrophy mice. **A** Flow cytometric analysis and histogram of ROS levels in isolated GC from mice after four courses of PBS, MSC, MSC + CytoD, MSC^sh−NC^, and MSCmiro1^Lo^ injections, respectively (PBS compared with MSC; MSC compared with MSC + CytoD; MSC^sh−NC^ compared with MSCmiro1^Lo^). **B** Flow cytometric analysis and histogram of MMP levels in GC from five groups of mice (as in A). **C** The OCR of GC from five groups of mice (as in A) was measured over time (in minutes), and the basal mitochondrial OCR, ATP production, and maximal respiration of those were calculated. **D** Histogram depicting the ATP levels in GC from five groups of mice (as in A). **E** Electron microscopic representation of GC slices from five groups of mice (as in A), mitochondria are indicated by red arrows. Scale bar, 2.0 μm. 500 nm. Data are means ± SD, *n* = 6. (*P < 0.05, **P < 0.01, ***P < 0.001, ****P < 0.0001 by two-sided unpaired Student’s t-test; nonsignificant differences not shown)

To validate our findings in an in vivo model, we treated 25 M mice with four courses of intramuscular injections of MSCs into the GC. MSC therapy significantly enhanced hindlimb grip strength (*P* < 0.01) (Fig. 4A), treadmill endurance (*P* < 0.01) (Fig. 4B), and GC mass (*P* < 0.05) (Fig. 4C) in mice. Histological analysis showed that in contrast to obvious myofiber loss in the model group, the treatment group exhibited significant recovery in myofiber quantity. (*P* < 0.01) (including fast and slow muscle fibers), Meanwhile, it upregulated the expression of myogenesis-related markers (MyoD and Myogenin, Supplementary Fig. S12) and reduced myofiber loss. And the cross-sectional area (CSA) of these fibers also increased (*P* < 0.05). Moreover, the ratio of slow muscle fibers to fast muscle fibers was higher (*P* < 0.001) (Fig. 4D), suggesting recovery of myofibers, especially fatigue-resistant slow muscle fibers. Immunofluorescence staining of different types of collagen showed that the contents of collagen I and collagen III were significantly increased (*P* < 0.0001), and the ratio of collagen I to collagen III was significantly elevated, suggesting improvements in tissue structure and function. However, Relative to the PBS group and MSC whole-cell group, the MSC-conditioned medium (CM) control group showed no meaningful efficacy, with efficacy observed solely in the whole-cell group—thus ruling out the dominant contribution of the paracrine effect. Protein expression of muscle atrophy markers Atrogin1 and MuRF1 was also reduced (*P* < 0.05) (Fig. 4E) Full-length blots are presented in Supplementary Figure S9. In addition, isolated GC tissues from mice treated with four courses of MSCs showed decreased ROS (*P* < 0.05) and enhanced MMP (*P* < 0.05), OCR (*P* < 0.05), and ATP levels (*P* < 0.0001), as well as improved mitochondrial structure and integrity as seen by TEM 24 h after MSCs transplantation (Fig. 5A–E). Again, these beneficial effects were negated by CytoD application (*P* < 0.05). Importantly, the effect of MSC therapy on muscle atrophy was absent when Miro1 was knocked down (*P* < 0.05), and TEM showed mitochondrial deformation, swelling, cavitation, and cristae breakage in the GC (Fig. 5E). These findings indicate that MSC-mediated improvements in muscle and mitochondrial function in aging mice rely on TNT-mediated MT and are critically dependent on Miro1.

### Miro1 regulates MT from MSCs to muscle cells without altering TNT formation

**Fig. 6:**
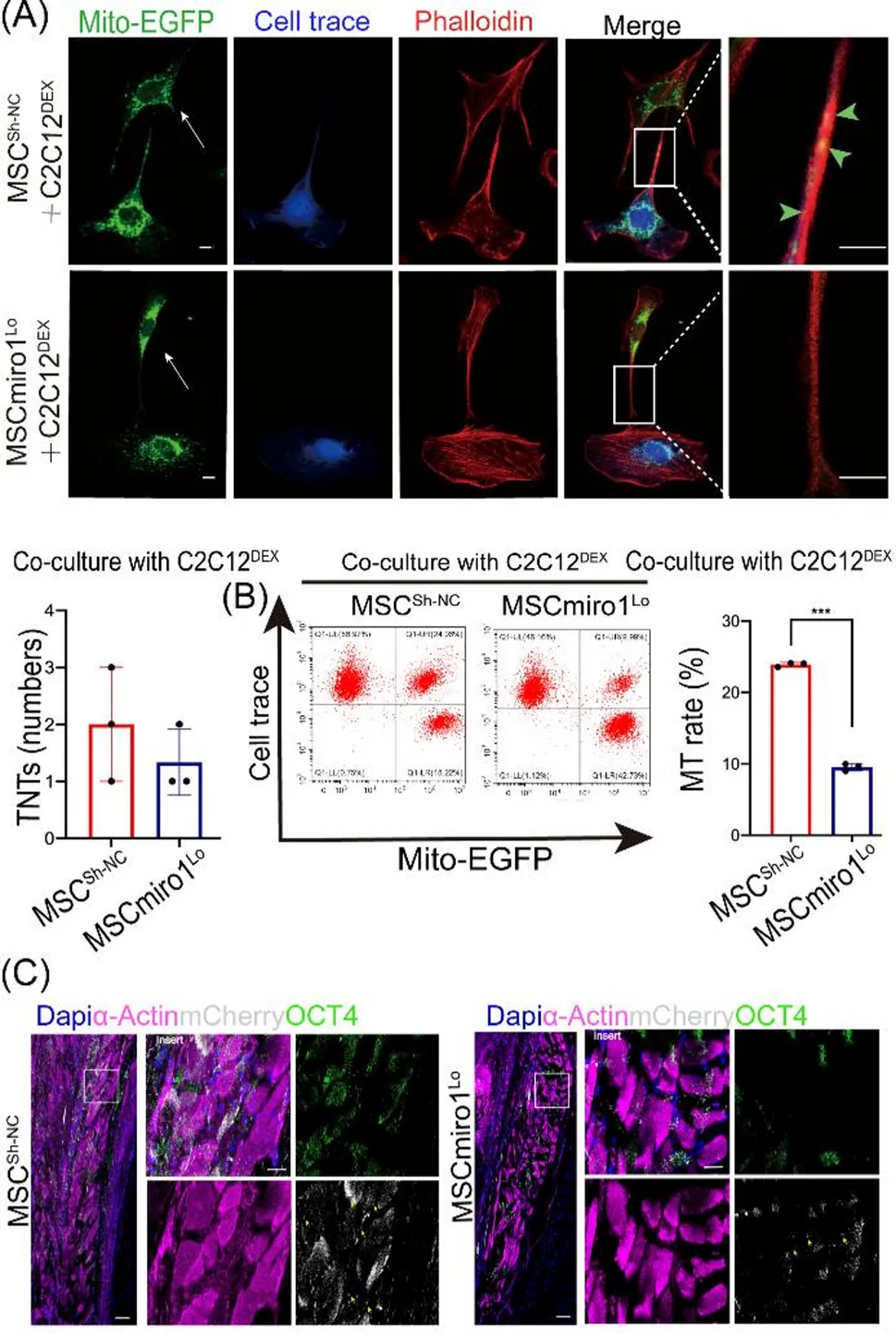
Miro1 regulates MT from MSCs to muscle cells without altering TNT formation. **A** Confocal observation of TNT formation and MT in C2C12^DEX^ co-culture system with MSC^sh−NC^ or MSCmiro1^Lo^. Mito-EGFP-labeled MSC^sh−NC^ or MSCmiro1^Lo^ (green), CellTrace-labeled C2C12^DEX^ (blue), and Phalloidin-labeled TNTs (red); white arrowheads indicate MSC^sh−NC^ or MSCmiro1^Lo^, and green arrowheads indicate Mito-EGFP signaling in TNTs. Scale bar, 20 μm. Histogram depicting the TNT number between C2C12^DEX^ and control MSC^sh−NC^ or MSCmiro1^Lo^. **B** FACS analysis of the rate of cells with double-positive signals in a co-culture system of CellTrace-labeled C2C12^DEX^ with Mito-EGFP-labeled MSC^sh−NC^ or MSCmiro1^Lo^ to suggest mitochondrial transfer rates. **C** Immunofluorescence staining of GC muscle sections from mice treated with four courses of MSC^sh−NC^ or MSCmiro1^Lo^ pre-labeled mitochondria with Mito-mCherry. After treatment, muscle sections were stained with OCT4 for MSCs (green), α-actin for muscle cells (red), and DAPI for nuclei (blue). Scale bar: 2.0 μm, 500 nm. Data are means ± SD, *n* = 3. (*P < 0.05, **P < 0.01, ***P < 0.001, ****P < 0.0001 by two-sided unpaired Student’s t-test; nonsignificant differences not shown)

Previous studies have pointed out that Miro1, in addition to playing an important role in intracellular mitochondrial dynamics and homeostasis, also regulates intercellular MT via TNTs [21]. We co-cultured Mito-EGFP-labeled MSC^sh−NC^ or MSCmiro1^Lo^ with CellTrace-labeled C2C12^DEX^. Confocal microscopy showed that the number of phalloidin-labeled TNTs was similar in both co-culture systems (*P*>0.05); however, the MSCmiro1^Lo^ group clearly had less mitochondrial signal within TNTs (Fig. 6A). Further analysis by FACS revealed that the MT rate of MSCmiro1^Lo^ was significantly decreased (*P* < 0.001) (Fig. 6B). The MT rate was calculated as the percentage of double-positive cells (CellTrace⁺/Mito-EGFP⁺) among the total C2C12 cells detected by flow cytometry. Additionally, in 25 M mice treated with Mito-mCherry-labeled MSC^sh−NC^ or MSCmiro1^Lo^ via GC injections, OCT4-positive MSCs were detected around α-actin-labeled myocytes at 6 h post-transplantation (results not shown), and at 24 h, mCherry-labeled mitochondria were evidently observed in muscle cells in the MSC^sh−NC^ group but were diminished in the MSCmiro1^Lo^ group (Fig. 6C). These findings indicate that while TNT formation remains unaffected, Miro1 is indispensable for efficient MT from MSCs to muscle cells, with its knockdown resulting in reduced mitochondrial translocation and compromised Miro1 compensation.

### Miro1-mediated MT improves the MQC system

**Fig. 7:**
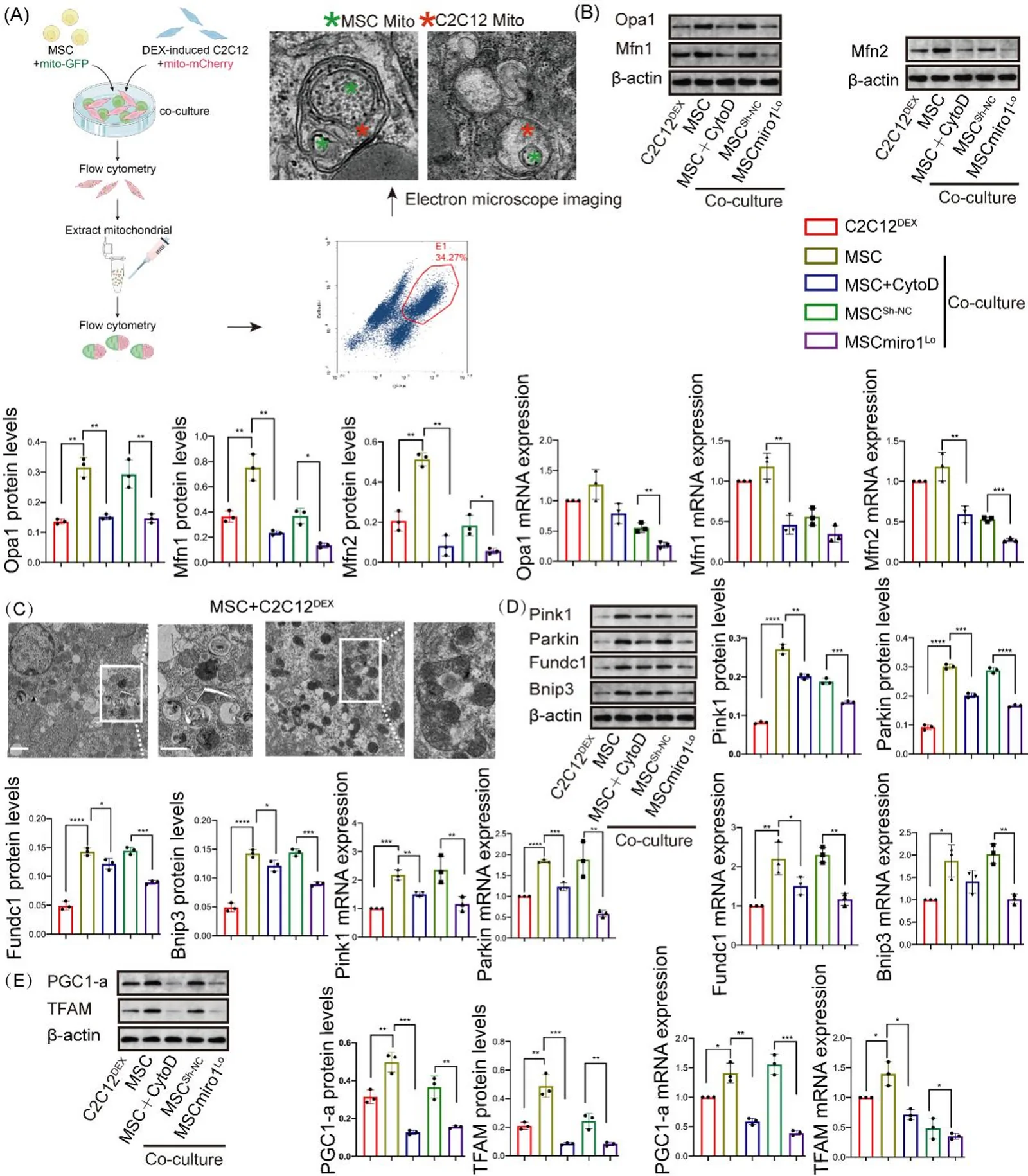
Miro1-mediated MT improves the MQC system. **A** Double-positive mitochondria from the co-culture system of Mito-EGFP-labeled MSCs with Mito-mCherry-labeled C2C12^DEX^ were screened by FACS, and TEM was performed to observe mitochondrial fusion. **B** Protein and mRNA expression levels of mitochondrial fusion markers in untreated C2C12^DEX^ or those sorted after co-culture with MSC, MSC + CytoD, MSC^sh−NC^, and MSCmiro1^Lo^, respectively. (Untreated C2C12^DEX^ compared with those after MSC co-culture; C2C12^DEX^ after MSC co-culture compared with those after MSC + CytoD co-culture; C2C12^DEX^ after MSC^sh−NC^ co-culture compared with those after MSCmiro1^Lo^ co-culture) (**C**) TEM observation of mitophagy in the co-culture system of MSC and C2C12^DEX^. **D** Protein and mRNA expression levels of mitophagy markers in C2C12^DEX^ under five different treatment conditions (as in B). **E** Protein and mRNA expression levels of mitochondrial biogenesis markers in C2C12^DEX^ under five different treatment conditions (as in B). Data are means ± SD, *n* = 3. (*P < 0.05, **P < 0.01, ***P < 0.001, ****P < 0.0001 by two-sided unpaired Student’s t-test; nonsignificant differences not shown)

To elucidate the mechanisms by which Miro1-mediated MT rescues damaged myocytes, we investigated key aspects of MQC, including fusion, mitophagy, and biogenesis. MSCs transduced with Mito-EGFP lentivirus were incubated with Gold Enhance EM plus and then co-cultured with Mito-mCherry-labeled C2C12^DEX^. Cells with double-positive signals (damaged myotubes that had taken up mitochondria from MSCs) were screened by FACS, and mitochondria were extracted; 32.41% of the mitochondria exhibited double-positive signals (E1 region, mitochondrial fusion rate). Under TEM, mitochondria originating from MSCs exhibited high-density electron signals on their outer membranes due to gold particle labeling. These labeled mitochondria have undergone heterotypic fusion with unlabeled mitochondria of C2C12^DEX^ (Fig. 7A). Protein and mRNA levels of mitochondrial fusion markers (Mfn1, Mfn2, and Opa1) were significantly elevated in myotubes sorted after MSC co-culture (*P* < 0.05), but were suppressed when CytoD was added to the culture medium or when Miro1 was knocked down in MSCs (*P* < 0.05) (Fig. 7B). The mtDNA/nDNA ratio was measured by qPCR, and no significant difference in mtDNA copy number was observed between the MSC co-culture group and the model group (Supplementary Fig. S13). This result ruled out the possibility that the upregulation of mitochondrial fusion-related genes was simply caused by an increase in total mitochondrial content, and further confirmed that the improvement in mitochondrial function resulted from enhanced mitochondrial quality control rather than increased mitochondrial quantity. Furthermore, TEM revealed mitophagosomes (left) and mitolysosomes (right) in the MSC and C2C12^DEX^ co-culture system, indicating enhanced mitophagy (Fig. 7C). Consistently, mitophagy-related markers (Parkin, Pink1, Fundc1, and Bnip3) were upregulated in MSC-treated myotubes (*P* < 0.05); however, these effects were diminished upon Miro1 knockdown or CytoD treatment (*P* < 0.05) (Fig. 7D). Western blot was used to detect the autophagic substrate p62 and the LC3-II/LC3-I ratio for autophagic flux assessment (Supplementary Fig. S16). Full-length blots are presented in Supplementary Figure S19-S22. We found that p62 expression was significantly downregulated in both the MSC co-culture group and the MSCmiro1Hi group (*P* < 0.01), and this downregulation was abolished after CytoD treatment. These results confirmed that Miro1-mediated mitochondrial transfer promotes the degradation of autophagic substrates. After treatment with chloroquine (CQ), an autophagolysosome inhibitor, the LC3-II/LC3-I ratio was markedly elevated compared with the untreated group (*P* < 0.05). However, no such alteration was observed in the MSCmiro1Lo group, which directly verified the activation of autophagic flux in a Miro1-dependent manner. In addition, MSC treatment ameliorated mitochondrial biogenesis in C2C12^DEX^, as evidenced by increased expression of PGC-1α and TFAM (*P* < 0.05) (Fig. 7E) Full-length blots are presented in Supplementary Figure S10. Quantitative analysis of mtDNA copy number by qPCR and immunofluorescence quantification of the mitochondrial specific marker Tomm20 demonstrated no significant difference in the total mitochondrial content between the Miro1-overexpressing MSC treatment group and the control group, ruling out the possibility that functional improvement was merely attributed to the increased mitochondrial abundance Supplementary Figure S15.Similar improvements in the expression of MQC-related markers were observed in the age-related muscle atrophy mouse model (Figure S1A, B) Full-length blots are presented in Supplementary Figure S11. These results confirm that Miro1-mediated MT of MSCs promotes mitochondrial heterologous fusion, improves mitophagy, and enhances biogenesis in damaged myocytes, which may key to the eventual restoration of mitochondrial function.

### Miro1 overexpression further enhances the protection offered by MSC therapy

**Fig. 8:**
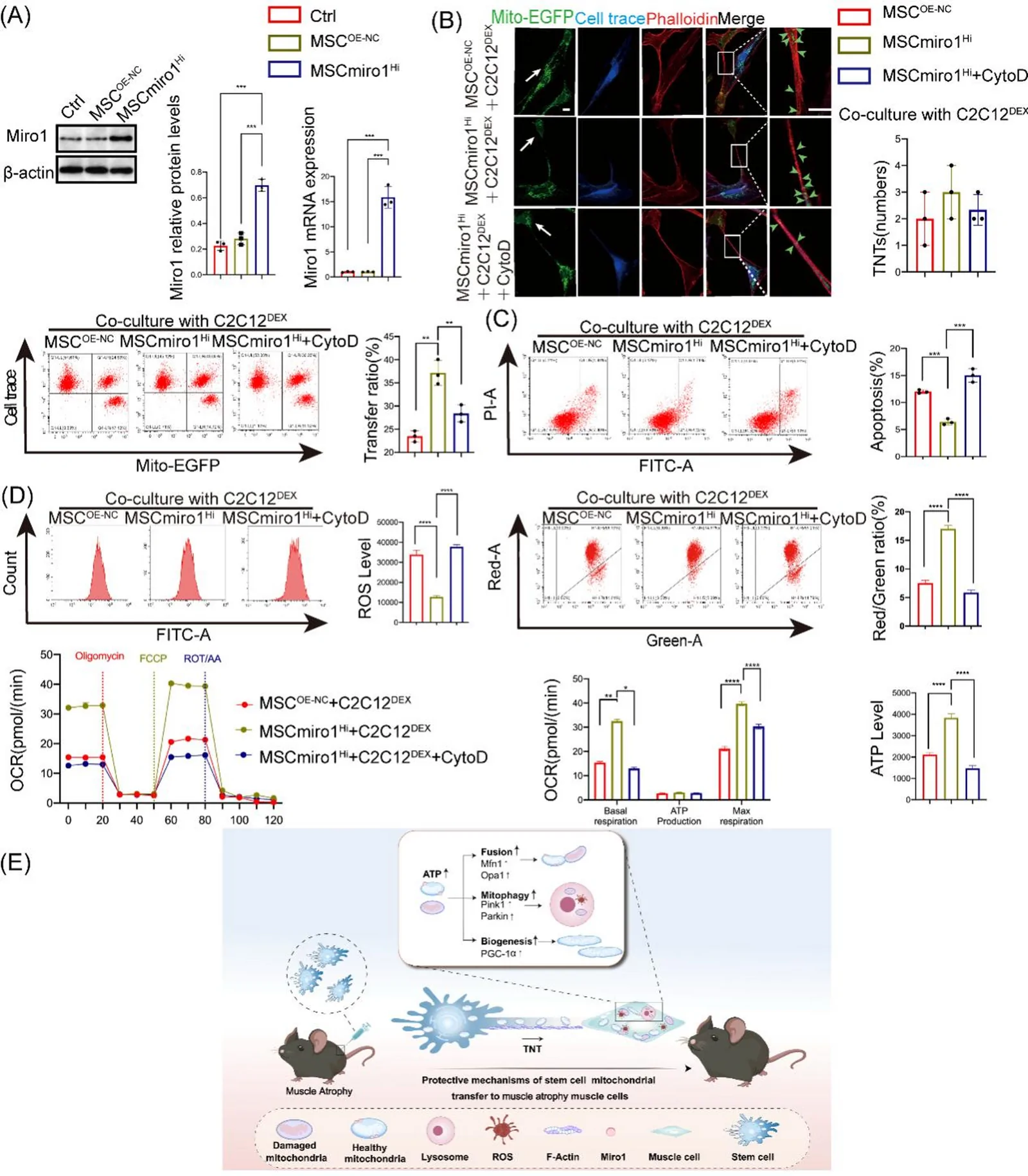
Miro1 overexpression further enhances the protection offered by MSC therapy. **A** Protein and mRNA expression of Miro1 in MSC transduced with a Miro1 lentiviral vector (MSCmiro1^Hi^) or an empty vector (MSC^OE−NC^) as a control was evaluated. **B** Confocal observation of TNT formation and MT in C2C12^DEX^ co-culture system with MSC^OE−NC^, MSCmiro1^Hi^, or MSCmiro1^Hi^ + CytoD. Mito-EGFP-labeled MSC^OE−NC^, MSCmiro1^Hi^, or MSCmiro1^Hi^ + CytoD (green), CellTrace-labeled C2C12^DEX^ (blue), and Phalloidin-labeled TNTs (red); white arrowheads indicate MSC^OE−NC^, MSCmiro1^Hi^, or MSCmiro1^Hi^ + CytoD., and green arrowheads indicate Mito-EGFP signaling in TNTs. Scale bar, 20 μm. Histogram depicting the TNT number between C2C12^DEX^ and MSC^OE−NC^, MSCmiro1^Hi^, or MSCmiro1^Hi^ + CytoD. FACS analysis of the ratio of cells with double-positive signals in these co-culture systems to suggest mitochondrial transfer rates. (MSC^OE−NC^ compared with MSCmiro1^Hi^; MSCmiro1^Hi^ compared with MSCmiro1^Hi^ + CytoD). **C** Flow cytometric examination and histogram of the apoptosis rates in C2C12^DEX^ under three different treatment conditions (as in B). **D** Analysis of mitochondrial functional indicators (ROS, MMP, OCR, and ATP) in C2C12^DEX^ under three different treatment conditions (as in B) (*n* = 6). **E** Schematic representation of the protective mechanisms of MSCs in muscle atrophy treatment. After GC injection of MSCs into muscle atrophy mice, MSCs transfer healthy mitochondria to damaged muscle cells via Miro1-mediated TNTs, enhancing mitochondrial function and muscle recovery. This process involves the upregulation of mitochondrial fusion (Mfn1, Opa1), mitophagy (Pink1, Parkin), and biogenesis (PGC-1α). Data are means ± SD. (*P < 0.05, **P < 0.01, ***P < 0.001, ****P < 0.000 using one-way ANOVA followed by Tukey’s test)

To further validate whether Miro1-overexpressing would provide extra benefits for MSC-based therapy and MT, MSCs were transduced with a Miro1 lentiviral vector (MSCmiro1^Hi^), which was confirmed by elevated Miro1 protein and mRNA levels compared to an empty vector control (MSC^OE−NC^) (*P* < 0.001) (Fig. 8A) Full-length blots are presented in Supplementary Figure S9. When co-cultured with C2C12^DEX^, MSCmiro1^Hi^ increased MT (*P* < 0.01) without affecting TNT formation (*P* >0.05) and had greater efficacy in reducing ROS generation (*P* < 0.0001), restoring MMP (*P* < 0.0001), OCR (*P* < 0.05), and ATP levels (*P* < 0.0001), as well as decreasing apoptosis (*P* < 0.001), whereas inhibition of TNTs with CytoD reduced the positive effects (*P* < 0.05) (Fig. 8B–D). Overexpression of the mitochondrial protein Milton and co-culture with C2C12^DEX^ revealed no mitochondrial transfer yet did not impair TNT formation (Figure S6). This suggests that Milton overexpression alone—without Miro or motor proteins—does not fundamentally improve mitochondrial transfer, whereas Miro1 is the most critical driver of the entire mitochondrial transfer process (Figure S6). In the muscle atrophy mouse model, transplantation of MSCs replenished Miro1 in GC; muscles treated with MSCmiro1^Hi^ showed higher Miro1 expression compared to those receiving MSC^OE−NC^ (Figure S2D). Correspondingly, MSCmiro1^Hi^ treatment further enhanced grip strength, treadmill running endurance, and increased GC mass, muscle fibers quantity、CSA, and the proportion of slow-twitch fibers, Muscle tissue recovery, while reducing the expression of Atrogin1 and MuRF1 (Figure S2A-C) Full-length blots are presented in Supplementary Figure S9. Immunofluorescence staining was performed to detect the satellite cell-specific marker Pax7(Supplementary Fig. S14). The number of Pax7-positive cells showed no significant difference between the MSC treatment group and the model group, confirming that the improvement of muscle atrophy by mitochondrial transfer was not mediated by satellite cell activation and de novo myonucleus formation. TEM showed superior mitochondrial integrity in MSCmiro1^Hi^-treated muscle tissues compared to MSC^OE−NC^ (Figure S2B), and sorted myocytes exhibited lower ROS and improved MMP, OCR, and ATP levels (Figure S3A-D). As expected, the efficacy of MSCmiro1^Hi^ was inhibited by CytoD (Figure S2A-C, Figure S3A-D). Furthermore, the expression of mitochondrial fusion, mitophagy, and biogenesis-related markers was significantly upregulated in MSCmiro1^Hi^-treated muscle tissues (Figure S4A-F, Figure S5A-C) Full-length blots are presented in Supplementary Figure S10-11. Together, these results demonstrate that Miro1-overexpressing MSCs enhance MT efficiency and Miro1 compensation, leading to improved MQC and augmented therapeutic effects on mitochondrial function and muscle mass in both cellular and mouse models.

## Discussion

Mitochondrial Rho GTPases Miro1/2 (Rhot1/2), localized on the outer mitochondrial membrane, are key regulators of mitochondrial transport and distribution by coupling mitochondria to kinesin- and dynein-dependent microtubule transport pathways [4]. It has been suggested that despite 60% sequence homology between Miro1 and Miro2, Miro1 plays a primary and major role in mitochondrial localization, transport, and homeostasis compared to Miro2, which acts as a backup [22]. Miro1 is essential for mitochondrial distribution, and its absence leads to perinuclear aggregation of mitochondria, which impairs the peripheral energy state of the cell, while restricting the perinuclear distribution of ROS affects the cellular response to elevated mitochondrial ROS [23]. Miro1 is also required for normal mitochondrial morphology, cristae architecture, and endoplasmic reticulum-mitochondria contact sites [6]. In addition, Miro1 is extensively involved in MQC mechanisms: it can regulate the mitochondrial fusion protein MFN and the splitting protein DRP1 to affect mitochondrial dynamics [24]; ubiquitination of Miro1 is necessary for parkin-associated autophagic clearance of damaged mitochondria [25]; and Miro1 can participate in the transport of mitochondrial contents or clearance of damage by promoting the formation of mitochondrial-derived vesicles (MDVs) [26].

Muscle atrophy is a reduction in muscle mass and function, including primary muscle atrophy (age-related) and secondary muscle atrophy (disease-induced). High-dose or long-term glucocorticoid administration induces muscle atrophy, and mitochondrial dysfunction and oxidative stress have been shown to be involved [27]. Here, we used chronological aging mice (primary muscle atrophy) and DEX-induced C2C12 cells (secondary) as models, confirming impaired mitochondrial function. Miro1 expression was significantly reduced in both, suggesting its downregulation may contribute to this impairment. But upregulating Miro1 in C2C12^DEX^ cells did not significantly improve mitochondrial function. This may be dueto the fact that the positive regulatory effect of Miro1 on mitochondrial dynamics depends on a sufficient number of healthy mitochondria, whereas the damaged mitochondria in muscle atrophy exceed the rescue capacity of direct Miro1 overexpression.

MT can regulate the energy metabolism balance between cells to adapt to the needs of the organism; mitochondria from healthy cells can compensate for damaged cells to restore their energy supply in case of tissue lesion; in addition, donor cells can eject impaired mitochondria into recipient cells for degradation to achieve quality control [28]. MT of MSCs is currently becoming widely used in promoting repair and regeneration of damaged tissues. When target cells are injured or under stress, the mitochondria released from them can be phagocytosed and degraded by MSCs, which stimulates the anti-apoptotic function of MSCs and triggers the transfer of healthy mitochondria from MSCs to recipient cells via TNTs [29]. Via Miro1-mediated mitochondrial transfer, MSCs donate healthy mitochondria to injured myocytes. On the one hand, they restore the integrity of the mitochondrial network through heterologous fusion; on the other hand, they activate mitophagy to eliminate damaged mitochondria. Meanwhile, MSCs upregulate signaling pathways associated with mitochondrial biogenesis. Ultimately, this rescues mitochondrial energy metabolism with increased ATP production, inhibits the ubiquitin–proteasome system via downregulation of Atrogin1 and MuRF1, reduces muscle protein degradation, and consequently ameliorates myofiber atrophy and functional impairment. We demonstrated that MSCs transfer their mitochondria to injured myotubes via TNTs by immunofluorescence and flow cytometry. We found that the expression of Miro1 in MSCs isolated and cultured from either young or old mice was significantly higher than that in myoblasts. Thus, we clarified that MSC treatment is a modality that can transfer mitochondria with relatively high Miro1 expression into the muscle atrophy model, which not only provides a sufficient amount of healthy mitochondria, but also effectively complements Miro1. Using mitochondrial function measurements, histopathology and mouse behavioral tests, we showed MSCs’ salvage effect, with reduced efficacy when Miro1 is downregulated—possibly linked to decreased Miro1 supplementation in muscle atrophy models. Notably, Miro1 directly affects mitochondrial function and regulates MT efficiency in MSCs [16–18]. Miro1 acts as a core molecular hub for MT from MSCs to muscle cells: it binds to Milton to stabilize mitochondrial outer membrane localization, forms the Miro1-Milton-kinesin ternary complex to drive microtubule-dependent mitochondrial transport via kinesin’s ATPase activity, and recruits SNARE proteins to mediate MSC-muscle cell membrane fusion, consequently facilitating mitochondrial release from MSCs and their subsequent uptake by muscle cells [30–31]. Our results also substantiate the notion that Milton overexpression does not directly initiate mitochondrial transfer but provides critical molecular and structural support via mitochondrial remodeling and enhanced intracellular transport. However, Milton overexpression alone fails to significantly improve transfer efficiency in the presence of Miro1 dysfunction—consistent with the core mechanism of mitochondrial transfer and further supporting Milton’s “rate-limiting regulatory” role. Our study revealed that Miro1-knockdown MSCs mediated a reduced number of MT to the muscle atrophy model without affecting TNT formation. It suggests that MSCs with low Miro1 expression end up with diminished therapeutic effects by reducing the number of mitochondria donations on the one hand and, on the other hand, due to the decreased Miro1 expression carried by these supplemented mitochondria. In contrast, up-regulation of Miro1 levels in MSCs showed better therapeutic effects in all aspects, which is of great significance for the application and optimization of MSC therapy in patients with muscle atrophy in clinical practice.

To further explore the specific mechanisms by which Miro1-regulated MSC treatment rescues mitochondrial function, we focused on the MQC system, which is important for maintaining mitochondrial structure and function in response to organismal demands (Fig. 8E). Mitochondria fuse to share mtDNA and metabolites, reduce harmful accumulation in individual mitochondria, and extend their network to prepare for high-energy needs. MFN1/2 controls outer mitochondrial membrane fusion, and OPA1 regulates inner membrane fusion. Studies show that MFN1/2 deficiency in mouse skeletal muscle causes mitochondrial dysfunction, oxidative stress, and muscle atrophy [32, 33]. DEX-induced muscle atrophy is associated with decreased OPA1 and Mfn2 expression [34, 35]. In the present study, we found that mitochondrial fusion protein levels were elevated after MSC treatment in muscle atrophy models. Since previous studies have shown that donor cell mitochondria fuse with the original mitochondrial network after transferring to recipient cells [36], in addition to the fact that Miro1 can regulate mitochondrial fusion calcium-dependently [37], we hypothesized that both mitochondrial donation and Miro1 supplementation may contribute to healthy mitochondria fusing with old mitochondria, thereby restoring mitochondrial mass and improving energy supply. Damaged mitochondria in cells under stress, aging or disease are cleared by mitophagy to adjust mitochondrial quantity/quality and maintain mitochondrial homeostasis and cellular energy balance. Mitophagy involves processes: MMP reduction, activation of mitophagy-related proteins, autophagosome encapsulation, lysosome fusion and degradation of damaged mitochondria, and involves PINK1, Parkin, BNIP3 and FUNDC1. While the correlation between aging and mitophagy is controversial across studies, most reports show lower expression of mitophagy-related proteins in aging mice’s skeletal muscle, and mitophagy impairment may drive accumulation of dysfunctional mitochondria in muscle aging [21]. However, Miro1 can recruit cytoplasmic Parkin translocation to mitochondria in a PINK1-independent manner by binding to CaN; when mitochondria are damaged, ubiquitination of Miro1 is necessary for Parkin enrichment into those mitochondria and subsequent mitophagy [25]. Autophagic substrate p62 and the LC3-II/LC3-I ratio for autophagic flux assessment MSCs activate autophagosome degradation in a Miro1-dependent manner, thereby refining the mechanistic understanding of autophagy and mitophagy.

In this study, elevated mitophagy in muscle atrophy models following mesenchymal stem cell (MSC) treatment may result from Miro1 supplementation. For mitochondrial biogenesis, coordination between mitochondrial and nuclear genomes is essential: PGC-1α activates the nuclear respiratory factor 1 (NRF1) promoter, which in turn drives the mitochondrial transcription factor A (TFAM) promoter, and TFAM thereby facilitates transcription of the mitochondrial genome [38]. It has been shown that DEX-induced muscle atrophy is associated with reduced expression of mitochondrial biogenesis-related proteins, including PGC-1α and TFAM [39]; PGC-1α declined in the muscles of elderly subjects [40]; and the overexpression of PGC-1α maintains mitochondrial function and muscle integrity by stimulating mitochondrial biogenesis and attenuating cell death [41]. Previous studies have indicated that MSCs can phagocytose and degrade cardiomyocyte-derived damaged mitochondria, thus stimulating mitochondrial biogenesis of MSCs [29], and further promote mitochondrial donation [42]. However, our study found elevated expression of PGC-1α and TFAM in models after MSC treatment, suggesting that MT from MSCs also activates mitochondrial biogenesis in recipient cells. Notably, in addition to providing mitochondria, MSCs can also exert their paracrine effect by transcribing and secreting various factors (such as cytokines and immunomodulatory proteins), thereby synergistically promoting processes like angiogenesis and immune regulation, aiding tissue repair. This represents a key focus of future research.

Only C57BL/6J inbred mice were used in this study, which fails to reflect human genetic diversity. Relevance to human biology is anchored in conserved human MSC-skeletal muscle TNT formation, high miro1 homology (≥ 95%) [43], and pathological overlap between the hindlimb immobilization (HLI) model and human muscle atrophy. Additionally, regarding clinical translation: the therapeutic efficacy of human-derived MSCs has not been evaluated, and species differences may affect the feasibility of clinical translation. Future studies may validate the findings in mice of different strains, explore the optimal administration parameters for miro1-overexpressing MSCs, and investigate in depth the downstream molecular pathways through which miro1 regulates TNTs.

In conclusion, our study revealed the presence of Miro1 downregulation-associated mitochondrial dysfunction in muscle atrophy models, and although it could not be rescued by direct overexpression of Miro1, MSC transplantation could mediate the selective translocation of Miro1-rich mitochondria into damaged skeletal muscle cells via TNTs. The donation of a sufficient amount of healthy mitochondria, along with the supplementation of Miro1 they carry, may contribute to the restoration of mitochondrial function, muscle mass, and strength through upregulation of mitochondrial fusion, mitophagy, and biogenesis. Meanwhile, the expression level of Miro1 in MSCs regulates their MT efficiency. Ultimately, the expression of Miro1 was shown to be positively correlated with the therapeutic effect of MSCs, which is important for guiding the clinical application of MSCs to improve the therapeutic efficiency in muscle atrophy.

Limitations: Only C57BL/6 inbred mice were used, human MSC therapeutic efficacy was not verified, and optimal in vivo administration regimens remain unexplored. In addition, although we confirmed that Miro1 drives tunneling nanotube-mediated mitochondrial transfer without altering nanotube formation, the precise downstream molecular cascades regulated by Miro1 during intercellular mitochondrial transport require further mechanistic investigation. Collectively, these constraints suggest that further preclinical validation across multiple animal strains and human cell models is necessary before clinical translation.

## Supplementary Information

Supplementary Material 1.

## Data Availability

The data that support the findings of this study are available on request from the corresponding author.
