# Supplementary material for: Miro1-mediated mitochondrial transfer boosts stem cell therapy for muscle atrophy via restoring mitochondrial homeostasis
Source: Stem Cell Res Ther. 2026 Jul 20;17:369. doi: 10.1186/s13287-026-05191-2 (PMC13670476; doi:10.1186/s13287-026-05191-2)
Supplement: Supplementary file 1 [file 13287_2026_5191_MOESM1_ESM.pdf]

Supplementary Data

Supplementary Tables

Supplementary Table 1. Lentiviral particles for transfection.

Lentiviral particles	vector	Order of vector elements	Company
Mito-EGFP	GV223	PCMV-EGFP-MCS-PURO	GeneChem Co., Ltd
MSC$^{Sh\text{-}NC}$	GV112	hU6-MCS-CMV-Puromycin	GeneChem Co., Ltd
MSCmiro1^{Lo}	GV341	hU6-MCS-CMV-Puromycin	GeneChem Co., Ltd
MSC$^{OE\text{-}NC}$	GV341	Ubi-MCS-3FLAG-SV40-puromycin	GeneChem Co., Ltd
MSCmiro1^{Hi}	GV341	Ubi-MCS-3FLAG-SV40-puromycin	GeneChem Co., Ltd
Mito-mCherry	VL3512	pLV-Mito-mCherry	Yingmao Prosperous
MSC$^{OE\text{-}NC}$	VL3001	pGMLV-EF1a-SV40T-Puro Lentivirus	Yeasen
MSCmilton^{Hi}	VL3001	pGMLV-EF1a-SV40T-Puro Lentivirus	Yeasen

Supplementary Table 2. Antibodies for the immunoassays.

Antibody	Host	Company	Catalog No.	Dilution
β-actin	Mouse	Santa CruZ	SC-47778	1:5000
GAPDH	Mouse	Abcam	ab8245	1:5000
Miro1	Mouse	Santa CruZ	Sc398520	1:200
FLAG	Mouse	Merck	F3165	1:2000
OPA1	Mouse	Abcam	Ab157457	1:3000
Mfn2	Mouse	Santa CruZ	Sc515647	1:200
Mfn1	Mouse	Santa CruZ	Sc166644	1:200
Pink1	Mouse	Abcam	ab186303	1:1000
Fundc1	Mouse	Abcam	Ab224722	1:3000
Parkin	Mouse	Abcam	Ab77924	1:2000
Bnip3	Mouse	Abcam	Ab109362	1:1000
TFAM	Mouse	Abcam	Ab307302	1:1000
PGC-1α	Mouse	Abcam	Ab54481	1:1000
Atrogin1	Mouse	Proteintech	67172-1-Ig	1:5000
MuRF1	Mouse	Proteintech	55456-1-AP	1:1000
MyHC (Myosin Heavy Chain)	Mouse	Santa CruZ	SC-53094	1:100
α -actin (SMA)	Mouse	Abcam	Ab7817	1:2000
OCT4	Mouse	Abcam	ab96899	1:250
anti-mouse, HRP		Jackson ImmunoResearch	115-035-003	1:3000
anti-rabbit, HRP		Jackson ImmunoResearch	111-035-144	1:3000

anti-Fast Myosin Skeletal Heavy Chain	Mouse	Abcam	ab91506	1:2000
anti-Slow Myosin Skeletal Heavy Chain	Mouse	Abcam	ab234431	1:2000
anti-Collagen I	Mouse	Abcam	ab270993	1:1000
anti-Collagen III	Mouse	Abcam	ab184993	1:1000
α-Tubulin	Mouse	Sigama	T6074	1:5000
MyoD	Mouse	PTG	18943-1-AP	1:200
Myogensin	Mouse	Abcam	ab124800	1:100
Tomm20	Mouse	CST	42406	1:200
Pax7	Mouse	Abcam	Ab187339	1:500
P62	Mouse	Abcam	Ab109012	1:500
LC3A/B	Mouse	Abcam	Ab62721	1:300
VDAC1	Mouse	Santa Cruz	sc-390996	1:200

Supplementary Table 3. Primers for the qRT–PCR analysis.

**Gene name**		**Primer sequence**
β-actin	Forward	5‘- CACGATGGAGGGGCCGGACTCATC -3’
	Reverse	5‘-TAAAGACCTCTATGCCAACACAGT-3’
GAPDH	Forward	5‘- TCAACGGCACAGTCAAGG -3’
	Reverse	5‘- TGAGCCCTTCCACGATG-3’
Rhot1	Forward	5‘- TGAGGCTGAGACCATTTGTG-3’
	Reverse	5‘-GAGGTGGAGGCATTTTGTGT -3’
Opa1	Forward	5‘- GATGACACGCTCTCCAGTGA-3’
	Reverse	5‘-TCGGGGCTAACAGTACAACC-3’
Mfn2	Forward	5‘-GCCAGCTTCCTTGAAGACAC-3’
	Reverse	5‘-AACTTTGTCCCAGAGCATGG -3’
Mfn1	Forward	5‘-GAAGATGGCATGGGAAGAAA-3’
	Reverse	5‘-GGGAAAACCGAAACACAATG-3’
Pink1	Forward	5‘-CCCACACCCTAACATCATCC-3’
	Reverse	5‘-ACTGGGAGTCTGCTCCTCAA -3’
Fundc1	Forward	5‘-GCGATGACGAATCATACGAA-3’
	Reverse	5‘-CAGTTGCAGCAAGTTTTCCA-3’
Parkin	Forward	5‘-GTGGTTGCTAAGCGACAGGG-3’
	Reverse	5‘-TGAGGTGCTCTGGGGTTCGT-3’
Bnip3	Forward	5‘-CTTCAGCAATGGCAATGGGAGCA-3’
	Reverse	5‘-TTGTGGTGTCTGGGAGCGAGGTG-3’

PGC1-a	Forward	5'-GACAGCTTTCTGGGTGGATTG-3'
	Reverse	5'-GGAGACTGGGCCGTTTAGT-3'
TFAM	Forward	5'-AACACCCAGATGCAAAACTTTCA-3'
	Reverse	5'- GACTTGGAGTTAGCTGCTCTTT-3'

I. Core Information of 9 Experimental Groups (C57BL/6J Mice, n=2/group, 3 independent replications, total n=6/group)

Group No.	Group Name	Core Intervention (all underwent right hindlimb immobilization, RHLI)	Group Purpose
1	PBS Group (Model + PBS)	Intragastroc nemius injection of 100 μL sterile PBS	Blank control for muscle atrophy model; verify model validity
2	CM Group (Model + CM)	Intragastroc nemius injection of 100 μL MSC-conditioned medium (cell-free)	Exclude the effect of soluble factors secreted by MSCs
3	MSC Group (Model + MSC)	Intragastroc nemius injection of 1×10^6 wild-type MSCs in 100 μL	Core intervention group; verify basic therapeutic effect of MSCs
4	MSC + CytoD Group	MSC injection + intraperitoneal injection of TNTs inhibitor Cytochalasin D (CytoD, 5 μg/kg/d)	Verify whether MSC efficacy depends on TNTs after TNTs inhibition
5	MSC $^{Sh\text{-}Nc}$ Group	Injection of MSCs transfected with miro1 negative control shRNA lentivirus	Negative control for gene transfection; exclude lentiviral vector effect
6	MSCmiro1^{Lo} Group	Injection of MSCs with miro1 low expression (shRNA interference)	Verify the effect of miro1 low expression on MSC-TNTs-muscle atrophy efficacy
7	MSC $^{OE\text{-}NC}$ Group	Injection of MSCs transfected with miro1 overexpression negative control lentivirus	Negative control for overexpression experiment; exclude vector interference
8	MSCmiro1^{Hi} Group	Injection of MSCs with miro1 overexpression	Verify whether miro1 overexpression enhances MSC efficacy
9	MSCmiro1^{Hi} + CytoD Group	Injection of miro1-overexpressing MSCs +	Verify that the synergistic effect of miro1 depends

		intraperitoneal injection of CytoD	on the TNTs pathway

II. a. Descriptive Statistics (Outcome Indicators: mean±SD, data conform to normality/homogeneity of variance)

Outcome Indicators	PBS	CM	MSC	MSC+CytoD	MSC $^{Sh-Nc}$	MSCmiro1^{Lo}	MSC $^{OE-NC}$	MSCmiro1^{Hi}	MSCmiro1^{Hi} + CytoD
**Primary Outcome Indicators**	-	-	-	-	-	-	-	-	-
Gastrocnemius Wet Weight (mg)	140.2±10.8	145.6±11.2	183.5±13.4	152.1±11.6	181.2±12.9	158.7±12.3	182.8±13.1	205.3±14.7	155.4±11.9
Myofiber Cross-Sectional Area (CSA, μm²)	78.6±10.3	82.4±10.8	120.5±15.2	89.3±11.5	118.7±14.8	95.6±12.1	119.2±15.0	138.4±16.6	92.7±11.8
Relative MyoG Expression (fold change)	0.5±0.1	0.6±0.1	2.8±0.5	0.9±0.2	2.7±0.4	1.2±0.3	2.7±0.5	3.9±0.6	1.0±0.2
**Secondary Outcome Indicators**	-	-	-	-	-	-	-	-	-
Number of TNTs per Myofiber	1.7±0.6	1.9±0.7	5.6±1.2	2.3±0.8	5.4±1.1	2.8±0.9	5.5±1.2	7.8±1.5	2.5±0.8
Relative miro1 Expression (fold change)	0.4±0.1	0.4±0.1	1.0±0.2	1.0±0.2	1.0±0.2	0.3±0.1	1.0±0.2	3.2±0.5	3.1±0.5
Serum CK Level	262.4±32.1	251.7±29.8	180.3±24.6	239.5±30.2	183.6±25.1	225.8±28.7	181.9±24.8	158.2±22.5	232.6±29.5

(U/L)									

Note: CytoD was purchased from Sigma-Aldrich (Cat. No. C8273), and the intervention duration was synchronized with MSC injection (lasting for 28 days); the transfection efficiency of miro1 was verified by qPCR (the overexpression group showed a 3.2-fold increase, and the low-expression group showed a 70% decrease).

III. b. Core Comparison Effect Sizes and 95% CIs (Cohen's d)

Comparison Pairs	Outcome Indicator	Cohen's d	95% CI	Effect Level	Mechanism Interpretation
Group 3 vs Group 1 (MSC **vs** Model Blank)	Gastrocnemius Wet Weight	3.68	[2.92, 4.44]	Large Effect	MSCs significantly improve muscle atrophy
Group 3 vs Group 4 (MSC **vs** MSC + CytoD)	Number of TNTs	4.25	[3.40, 5.10]	Large Effect	MSC-induced TNTs depend on CytoD-sensitive pathways
Group 8 vs Group 3 (MSCmiro1^{Hi} **vs** MSC)	MyoG Expression	2.03	[1.35, 2.71]	Large Effect	miro1 overexpression enhances the myogenic effect of MSCs
Group 8 vs Group 9 (MSCmiro1^{Hi} **vs** MSCmiro1^{Hi} + CytoD)	Myofiber CSA	3.82	[3.05, 4.59]	Large Effect	The synergistic effect of miro1 depends on TNTs
Group 6 vs Group 3 MSCmiro1^{Lo} **vs** MSC)	Number of TNTs	3.15	[2.40, 3.90]	Large Effect	miro1 low expression impairs the TNT-promoting ability of MSCs
Group 5 vs Group 3 (MSC$^{Sh\text{-}Nc}$ **vs** MSC)	Gastrocnemius Wet Weight	0.19	[-0.48, 0.86]	No Effect	Interference vectors do not affect MSC efficacy

Methods and Materials

Cell Culture

Mouse bone marrow MSCs and C2C12 myoblasts were provided by Abiowell Biotechnology. MSCs were directly used in the treatment of muscle atrophy models. C2C12 were cultivated in DMEM with 10% FBS. At 70%-80% confluence, they were induced to differentiate in DMEM with 2% heat-inactivated horse serum for 4 days with daily medium changes. Muscle atrophy was modeled by treating cells with 1 μM dexamethasone (DEX).

Animals

25-month-old male C57BL/6J mice were acquired from (Charles River Laboratories) which were randomly divided into experimental (MSC intervention) and control (MSC-conditioned medium, CM) groups (n=6/group). Prior to interventions, all mice were acclimatized for 7 days in an SPF barrier environment (22–28 °C, 40%–70% relative humidity, 12-h light/dark cycle; 5 mice/cage, ad libitum diet/water). The initial body weight of the mice ranged from 28 to 32 g upon arrival. On Day 0, both groups underwent hindlimb immobilization under anesthesia (5% isoflurane induction, 2–3% maintenance with 1 L/min oxygen flow). The experimental group received 4 weekly intramuscular injections (Day 0/7/14/21) of passage 3 murine bone marrow-derived MSCs (1×10^6 cells/100 μL sterile PBS, ≥95% purity) into the GC; the control group received 100 μL 0.22 μm-filtered MSC-CM via the same route and frequency. All procedures were performed in a sterile surgical suite. Daily health monitoring (body weight, activity) and biweekly GC diameter measurement were performed; euthanasia and tissue collection were conducted on Day 28. Twenty-four hours after the last injection, the mice were euthanized by cervical dislocation. The bilateral gastrocnemius muscles were quickly dissected and isolated, and residual blood was rinsed with pre-cooled PBS. A part of the muscle tissue was snap-frozen in liquid nitrogen and then transferred to a -80 °C ultra-low temperature refrigerator for subsequent molecular biology detection; the other part was fixed with 4% paraformaldehyde for pathological section preparation. After the experiment, the mouse carcasses were temporarily stored in a dedicated -20 °C freezer at the Animal Center. Once the freezer was full, the carcasses were transferred to a professional solid waste disposal company with the "Hazardous Waste Operation Permit" for centralized harmless disposal. The disposal process strictly complied with the "Guidelines for Ethical Review of Laboratory Animal Welfare"、 "The ARRIVE guidelines 2.0." and local environmental protection regulations. Rationale: HLI established a clinically relevant muscle atrophy model; targeted GC injection maximized local intervention efficacy (aligning with the hypothesis that MSCs alleviate atrophy via regulating tunneling nanotubes); CM control clarified MSC-specific effects by excluding soluble factor interference. Animal Ethics Committee of the Second Affiliated Hospital, Zhejiang University School of Medicine (Approval Number: 2024-230; Approval Date: October 25, 2024).

Isolation and Culture of Primary Myoblasts and Bone Marrow-Derived MSCs in Mice

Primary myoblasts and MSCs were isolated from 2M and 25M mice to compare Miro1 expression levels. gastrocnemius(GC) were aseptically dissected, minced (~0.1 cm^3), washed three times with PBS (discarding the supernatant containing floating debris), then digested with 0.25% trypsin-EDTA at 37°C for 30 min with gentle pipetting every 5 min. Digestion was stopped with myoblast growth medium (Ham's F-10 + 20% FBS, 1%

penicillin-streptomycin, 2.5 ng/mL bFGF), and the suspension was filtered through a 70 μm cell strainer, centrifuged at 1000 rpm for 10 min, and resuspended in growth medium. Cells were pre-plated for 1 h at 37°C to remove fibroblasts, and the non-adherent myoblasts were then transferred to a T25 flask. Medium was changed every 4 days in the first week and daily afterwards. Myoblasts adhered within 12 h, became spindle-shaped at 72 h, and began aligning as they proliferated. When confluence exceeded 80%, myoblasts fused into multinucleated myotubes and were collected for further analysis. For MSCs isolation, femurs and tibias were isolated. The epiphyses were trimmed with ophthalmic scissors, and bone marrow was flushed out with MSCs growth medium (α-MEM supplemented with 10% FBS and 1% Gluta MAX) using a 25G needle. The suspension was filtered through a 70 μm strainer, centrifuged at 1000 rpm for 5 min, and the pellet was resuspended in MSCs growth medium and transferred to a T25 flask. Non-adherent cells were removed by changing the medium after 24 h and then every 2–3 days. MSCs formed colonies within 6–9 days, reaching 80–90% confluence with a fibroblast-like morphology.

Lentiviral Transfection

The lentiviral particles (Table S1) for mitochondrial labeling in MSCs were generated from the pCMV-Mito-EGFP-puro vector construct. MSCs were seeded in 6-well plates and transduced with the lentivirus at a titer of 10 TU/mL for 12 hours following the manufacturer's protocol (GeneChem Co., Ltd, Shanghai, China). Post-transduction, cells were cultured for an additional 72 hours to allow for stable expression. Four experimental groups were established: Miro1-knockdown MSCs ($MSCmiro1^{Lo}$), non-targeting shRNA control ($MSC^{Sh\text{-}NC}$), empty vector control ($MSC^{OE\text{-}NC}$), and Miro1-overexpressing MSCs ($MSCmiro1^{Hi}$). empty vector control ($MSC^{OE\text{-}NC}$), and Milton-overexpressing MSCs ($MSCmilton^{Hi}$) (Yeasen). These cell models were utilized in subsequent functional assays.

Flow Cytometry (FC) and Fluorescence-Activated Cell Sorting (FACS)

DEX-induced C2C12 were labeled with CellTrace (Invitrogen) and co-cultured with MSCs for 24 h. Labeled cells were isolated via FACS for subsequent assays, including ROS, MMP, apoptosis, and MQC protein analysis. For apoptosis, cells were incubated with 1 μL PI and 5 μL Annexin V (BD Pharmingen) in 1× binding buffer for 15 min in the dark, then analyzed by FC. MT efficiency was quantified by co-culturing CellTrace-labeled C2C12 with Mito-EGFP-labeled MSCs and detecting dual-positive cells via FACS.

Mitochondrial Function Measurement

ROS levels were measured by incubating cells with DCFH-DA (Beyotime) for 20 min and analyzing fluorescence via FC. MMP was evaluated via JC-1 staining (20 min incubation) with the red/green fluorescence ratio determined by FC. Mitochondrial respiration was assessed using the XF96 Extracellular Flux Analyzer (Agilent) with 1×10^4 cells per well in XF base medium (25 mM glucose, 2 mM pyruvate-glutamine, 1 mM pyruvate). Oligomycin (1.5 μM), FCCP (0.5 μM), and antimycin A/rotenone (1.0 μM) were used. Expressed as either "pmol O_2/min per 10^4 C2C12 cells" (normalized to viable cell count via hemocytometer) or "pmol O_2/min/μg mitochondrial protein" (mitochondria isolated by differential centrifugation, normalized to protein concentration via BCA assay). Intracellular ATP was quantified from cell lysates (0.5% Triton X-100, 100 mmol/L glycine, pH 7.4) Levels expressed as "pmol/μg total cellular protein", normalized to total cellular protein concentration (BCA assay).using an ATP bioluminescent assay kit (Sigma).

Immunofluorescence (IF) and Immunohistochemistry (IHC)

Cells were fixed in 4% formaldehyde (30 min), permeabilized with 0.1% Triton X-100 in PBS, and blocked with goat serum (1 h). Primary antibodies (Table S2) were applied overnight at 4 °C, followed by Alexa Fluor secondary antibodies (1 h). Cryostat-prepared muscle sections (5–10 μm) were similarly processed. Images were acquired using a confocal microscope (63×). After 4% paraformaldehyde fixation, muscle tissues were paraffin-embedded, sectioned at 5 μm, deparaffinized, and subjected to H&E and Masson staining. After antigen retrieval, deactivating endogenous peroxidases (3% H_2O_2, 15 min), and blocking (10% normal goat serum, 30 min), sections were incubated overnight at 4 °C with anti-fast and anti-slow myosin heavy chain antibodies. followed by a 60-min incubation with a secondary antibody and DAB staining. Images were captured with an Olympus BX53 microscope, and fiber cross-sectional areas were quantified using Image-Pro Plus.

Quantitative Real-Time PCR (RT-qPCR)

Total RNA was isolated using TRIzol (Takara), and cDNA was synthesized with the iScript Kit (Bio-Rad). RT-qPCR was performed with Taq Universal SYBR Green Supermix on the CFX Manager Software (Bio-Rad); gene expression was calculated by the $2^{-\Delta\Delta Ct}$ method (primer sequences in Table S3).

Western Blot (WB)

Proteins were resolved by SDS-PAGE, transferred to PVDF membranes (Millipore), blocked with 5% skim milk in TBST (2 h), and incubated with primary antibodies (Table S2) overnight at 4 °C. After incubation with HRP-conjugated secondary antibodies (1 h), bands were visualized using ECL (Millipore) and quantified with ImageJ.

Co-Immunoprecipitation (Co-IP)

The target protein antibody/IgG was conjugated to Protein A/G beads (4 °C, 6-8 h). Approximately 1×10^7 MSCs were lysed in 1 mL RIPA buffer, and lysates were incubated with the antibody-bead complex overnight at 4 °C. After washing with 0.1% NP-40 in PBS, the immunocomplex was eluted and analyzed by WB.

Hindlimb Grip Strength and Treadmill Endurance Test

Mice were acclimated before testing. For grip strength, mice were trained to grasp a horizontal grid connected to an electronic dynamometer using hindlimbs. The mouse was gently pulled backward in a horizontal direction until it released the grip, and the force exerted was recorded. Three trials were averaged. For endurance, after a 2-day acclimation, mice ran at 8 m/min for 20 min, with speed increased by 0.2 m/min until exhaustion (hindlimbs on grid >10 s).

Mitochondrial Isolation

Using the Cell Mitochondria Isolation Kit (MCE, HYK1060) at 4 °C, 1×10^7 cells were resuspended in 2 mL isolation reagent with 1 mM PMSF and incubated on ice for 10-15 min. The suspension was homogenized for 30 min and centrifuged at 750×g (10 min). The supernatant was collected, combined with that from a second homogenization, and centrifuged at 10,000×g (15 min). The mitochondrial pellet was resuspended in 20-25 μL extraction buffer.

Transmission Electron Microscopy (TEM)

Cells or tissues were fixed in 4% phosphate-buffered glutaraldehyde, dehydrated in graded ethanol, embedded in epoxy resin. and sectioned (95-100 nm). Sections were post-stained with uranyl acetate and Gold Enhance EM Plus (2114), then examined using a JEOL TEM.

Statistical Analysis

All statistical analyses were performed using SPSS 26.0 software. Data were first tested for normality using the Shapiro-Wilk test and for homogeneity of variance using Levene's test. Normally distributed data were expressed as mean ± standard deviation (mean ± SD), Two-group comparisons used the t-test, and multiple groups were analyzed by one-way or two-way ANOVA with Bonferroni post hoc. Non-normally distributed data were expressed as median (interquartile range) [M (IQR)] and analyzed by the Mann-Whitney U test. $P < 0.05$ was considered significant.

Supplementary Figures

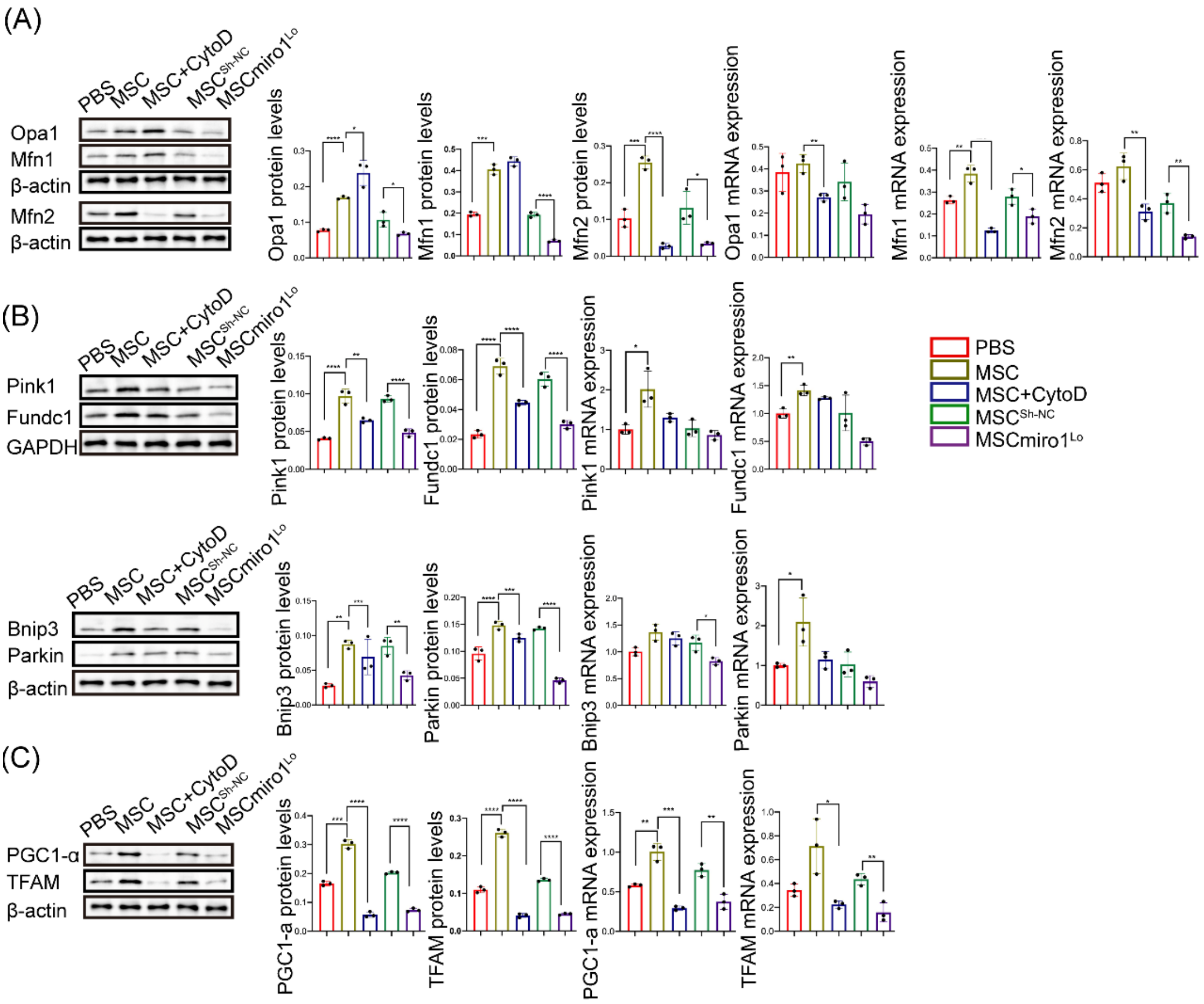

Supplementary Figure S1. Analysis of protein and mRNA expression levels related to mitochondrial quality control mechanism in gastrocnemius muscle of mice with muscle atrophy after 4 courses of injection of PBS, MSC, MSC + CytoD, MSC$^{sh\text{-}NC}$ and MSCmiro1^{Lo}, respectively. (PBS compared with MSC; MSC compared with MSC + CytoD; MSC$^{sh\text{-}NC}$ compared with MSCmiro1^{Lo}) **(A)** Mitochondrial fusion **(B)** Mitophagy **(C)**

Mitochondrial biogenesis. Data are means ± SD, n = 3. (*$P < 0.05$, **$P < 0.01$, ***$P < 0.001$, ****$P < 0.0001$ by two-sided unpaired Student's t-test; nonsignificant differences not shown).

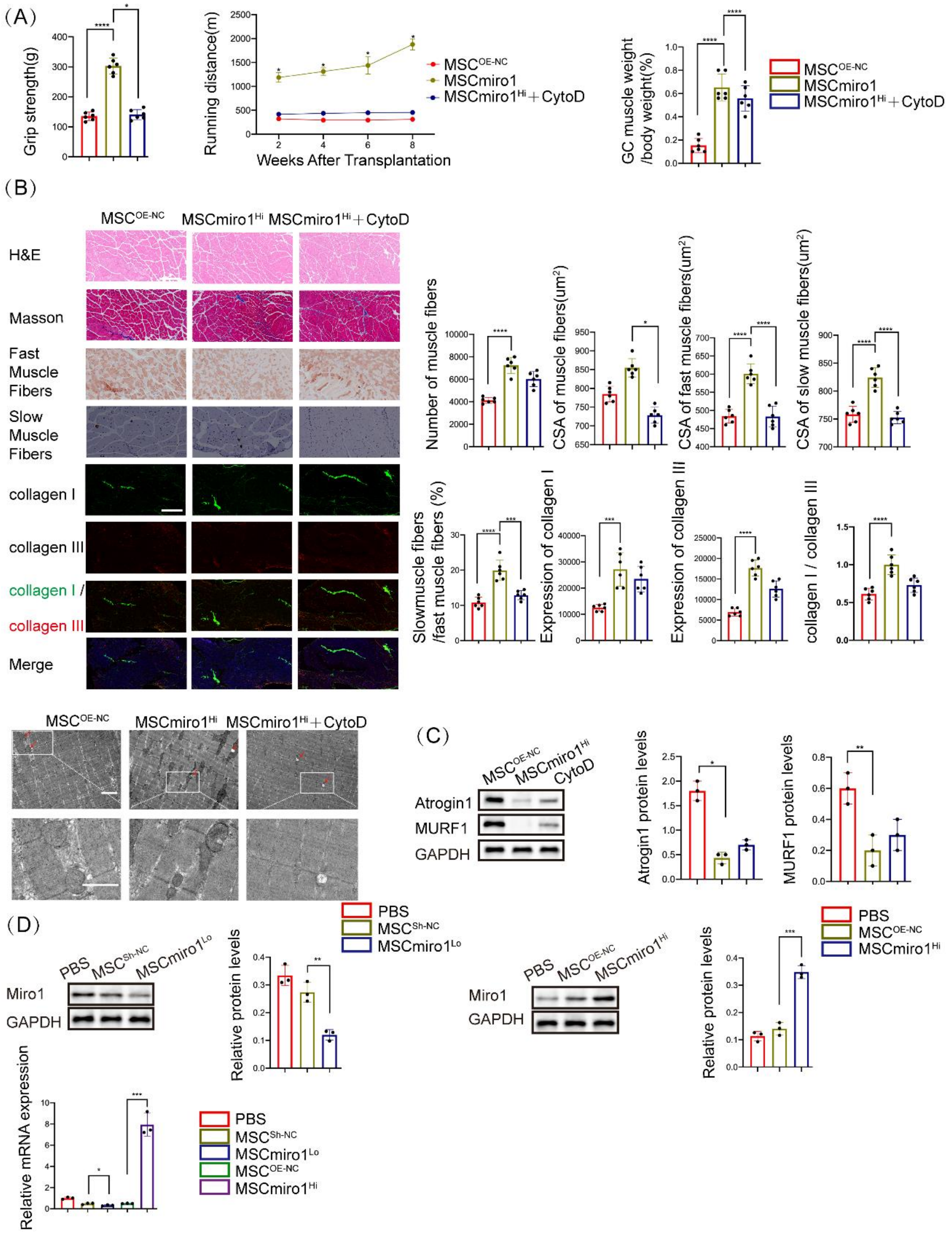

Supplementary Figure S2. Analysis of therapeutic effects of MSC^{OE-NC} 、MSCmiro1^{Hi} and MSCmiro1^{Hi} + CytoD in muscle atrophy mice, respectively. (MSC^{OE-NC}compared with MSCmiro1^{Hi}; MSCmiro1^{Hi} compared with MSCmiro1^{Hi} + CytoD) **(A)** Hindlimb grip strength, running distance, and GC muscle-to-body weight ratio. **(B)** H&E staining, Masson staining (scale bar, 250 μm), and immunohistochemical staining (scale bar, 250 μm) of GC fibers. Measure and count the CSA of myofibers, fast and slow fibers, slow-to-fast fiber ratio. Electr

on microscopic representation of GC tissue slices, mitochondria are indicated by red arrows. Scale bar, 2.0 μm. 5 00 nm. Immunofluorescence expression of collagen I and collagen III，The ratio of collagen I to collagen III in f ast-twitch muscle-dominated muscles） collagen III(slow-twitch muscle-dominated muscles) (scale bar, 250 μ m) (n = 6) **(C)** Protein expression levels of Atrogin1, MuRF1 in GC. **(D)** Protein and mRNA expression levels of Miro1 in GC of five groups of mice (PBS, $MSC^{sh\text{-}NC}$, $MSCmiro1^{Lo}$, $MSC^{OE\text{-}NC}$, $MSCmiro1^{Hi}$). Data are means ± S D. (*$P < 0.05$, **$P < 0.01$, ***$P < 0.001$, ****$P < 0.0001$ by two-sided unpaired Student's t-test; nonsignificant differences not shown).

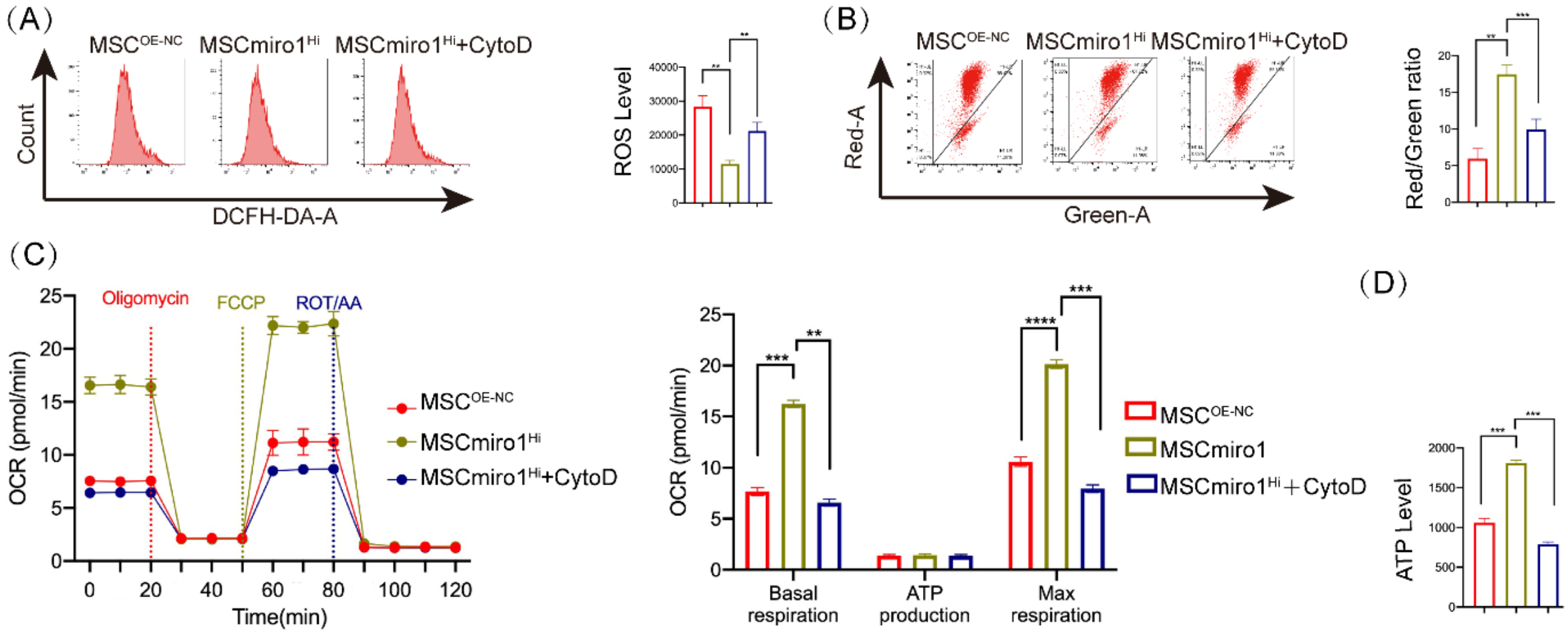

Supplementary Figure S3. The mitochondrial function of mice with muscle atrophy was detected after 4 courses of injection of MSC^{OE-NC}, MSCmiro1^{Hi} and MSCmiro1^{Hi} +CytoD, respectively. (MSC^{OE-NC}compared with MSCmiro1^{Hi}; MSCmiro1^{Hi} compared with MSCmiro1^{Hi} + CytoD) **(A)** ROS Level. **(B)** MMP Level. **(C)** OCR. **(D)**ATP Level. Data are means ± SD, n = 6. (**P* < 0.05, ***P* < 0.01, ****P* < 0.001, *****P* < 0.0001 by using one-way ANOVA followed by Tukey's test; nonsignificant differences not shown).

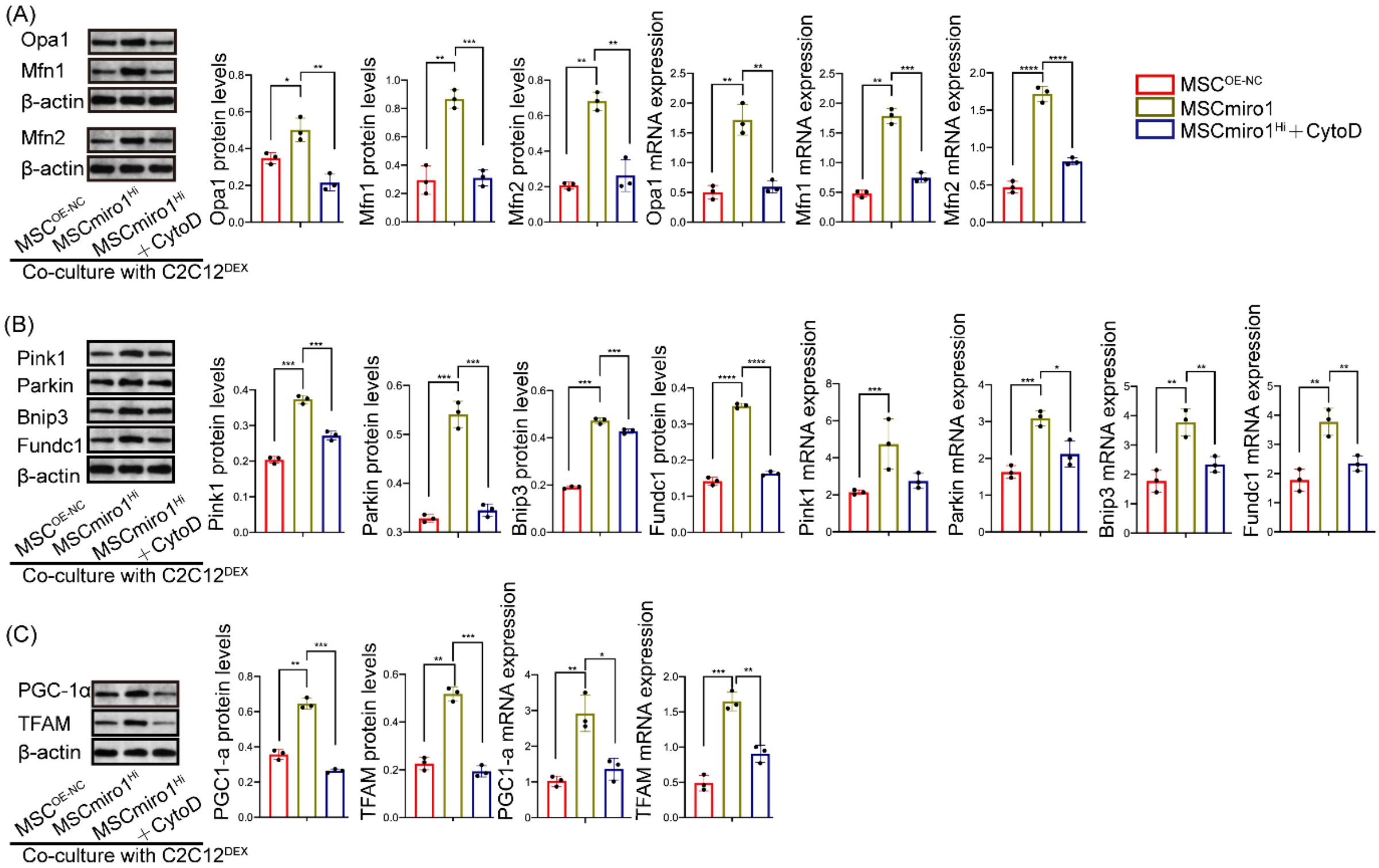

Supplementary Figure S4. Analysis of protein and mRNA expression levels related to mitochondrial quality control mechanism in untreated C2C12^{DEX} or those sorted after co-culture with MSC$^{OE\text{-}NC}$, MSCmiro1^{Hi}, MSCmiro1^{Hi} + CytoD, respectively. (MSC$^{OE\text{-}NC}$compared with MSCmiro1^{Hi}; MSCmiro1^{Hi} compared with MSCmiro1^{Hi} + CytoD) **(A)** Mitochondrial fusion. **(B)** Mitophagy. **(C)** Mitochondrial biogenesis. Data are means ± SD, n = 3. (**P* < 0.05, ***P* < 0.01, ****P* < 0.001, *****P* < 0.0001 by two-sided unpaired Student's t-test; nonsignificant differences not shown).

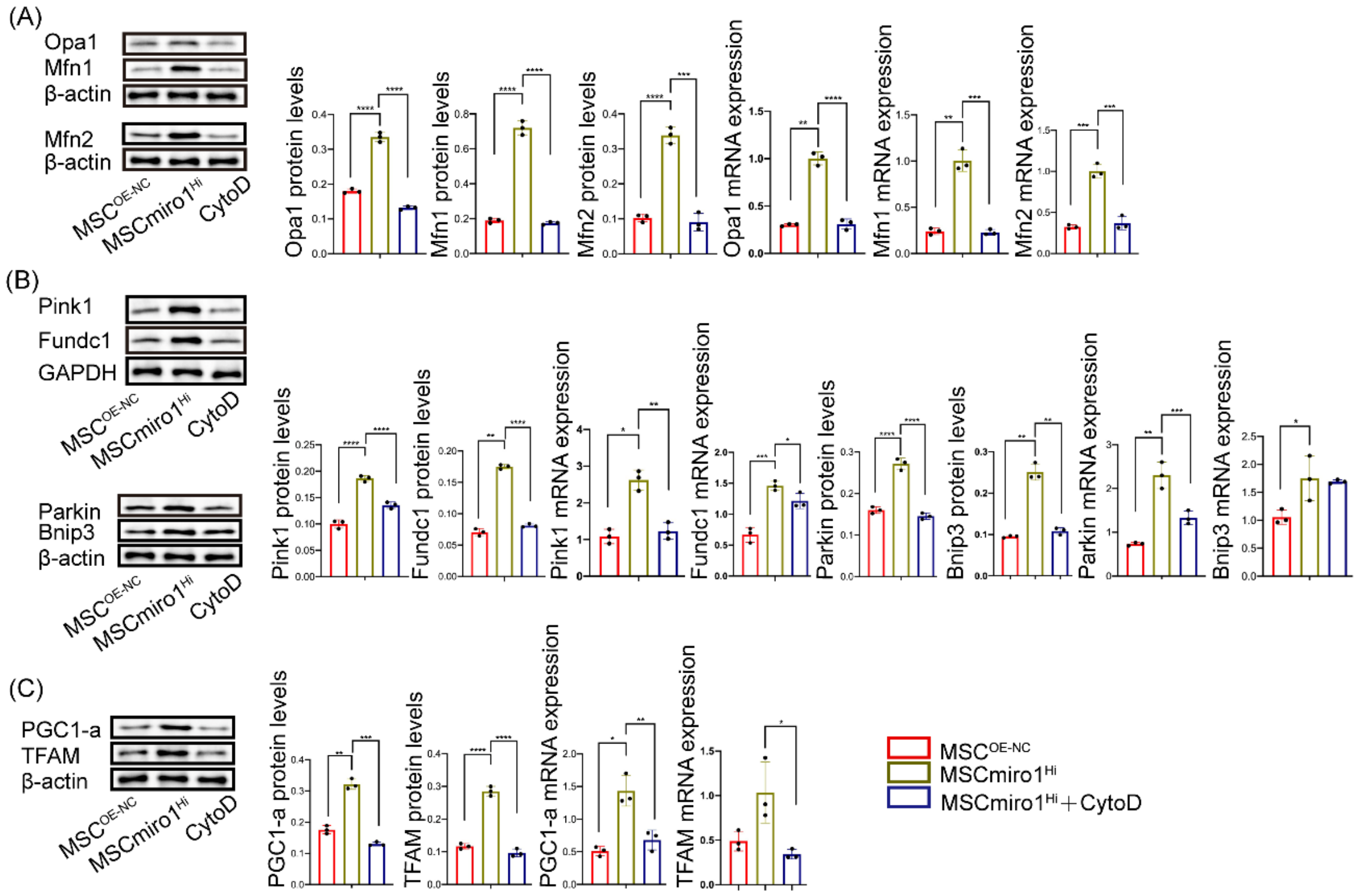

Supplementary Figure S5. Analysis of protein and mRNA expression levels related to mitochondrial quality control mechanism in gastrocnemius muscle of muscle atrophy mice treated with MSC$^{OE\text{-}NC}$, MSCmiro1^{Hi} and MSCmiro1^{Hi} +CytoD, respectively. (MSC$^{OE\text{-}NC}$ compared with MSCmiro1^{Hi}; MSCmiro1^{Hi} compared with MSCmiro1^{Hi} + CytoD)**. (A)** Mitochondrial fusion. **(B)** Mitophagy. **(C)** Mitochondrial biogenesis. Data are means ± SD, n = 3. (**P* < 0.05, ***P* < 0.01, ****P* < 0.001, *****P* < 0.0001 by two-sided unpaired Student's t-test; nonsignificant differences not shown).

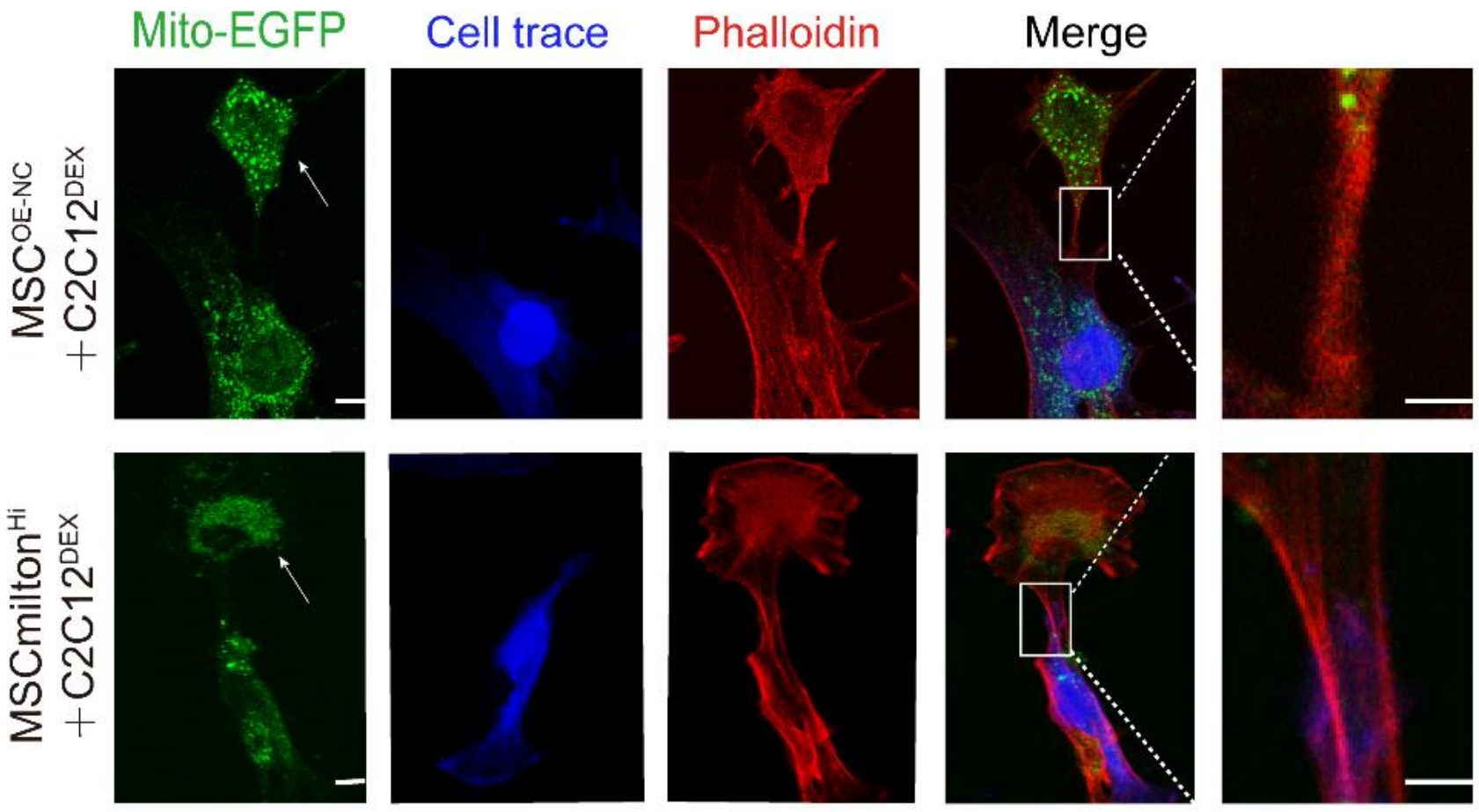

Supplementary Figure S6. Miro1 overexpression further enhances the protection offered by MSC therapy. Confocal observation of TNT formation and MT in C2C12^{DEX} co-culture system with MSC^{OE-NC}, MSCmilton^{Hi} Mito-EGFP-labeled MSC^{OE-NC}, MSCmilton^{Hi}, (green), CellTrace-labeled C2C12^{DEX} (blue), and Phalloidin-labeled TNTs (red); white arrowheads indicate MSC^{OE-NC}, MSCmilton^{Hi}, Scale bar, 20 μm.

Supplementary FigureS7

FIG.1B

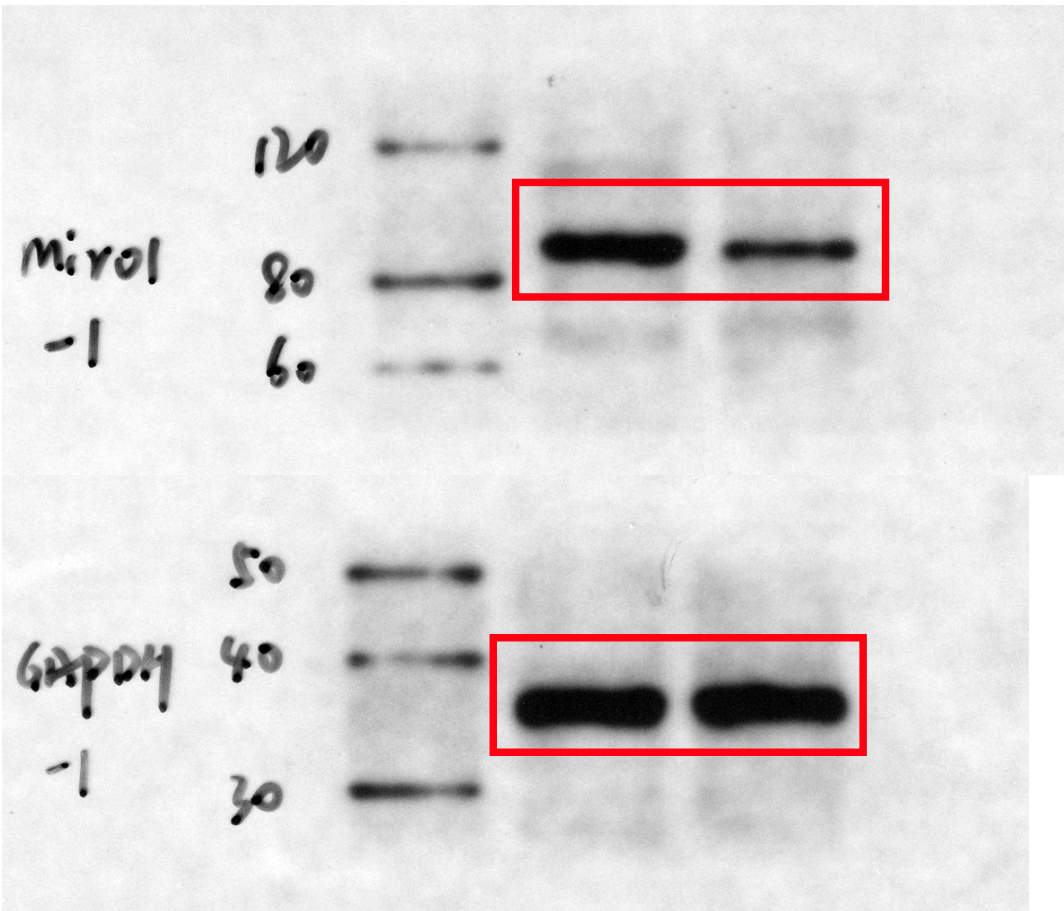

FIG.1C

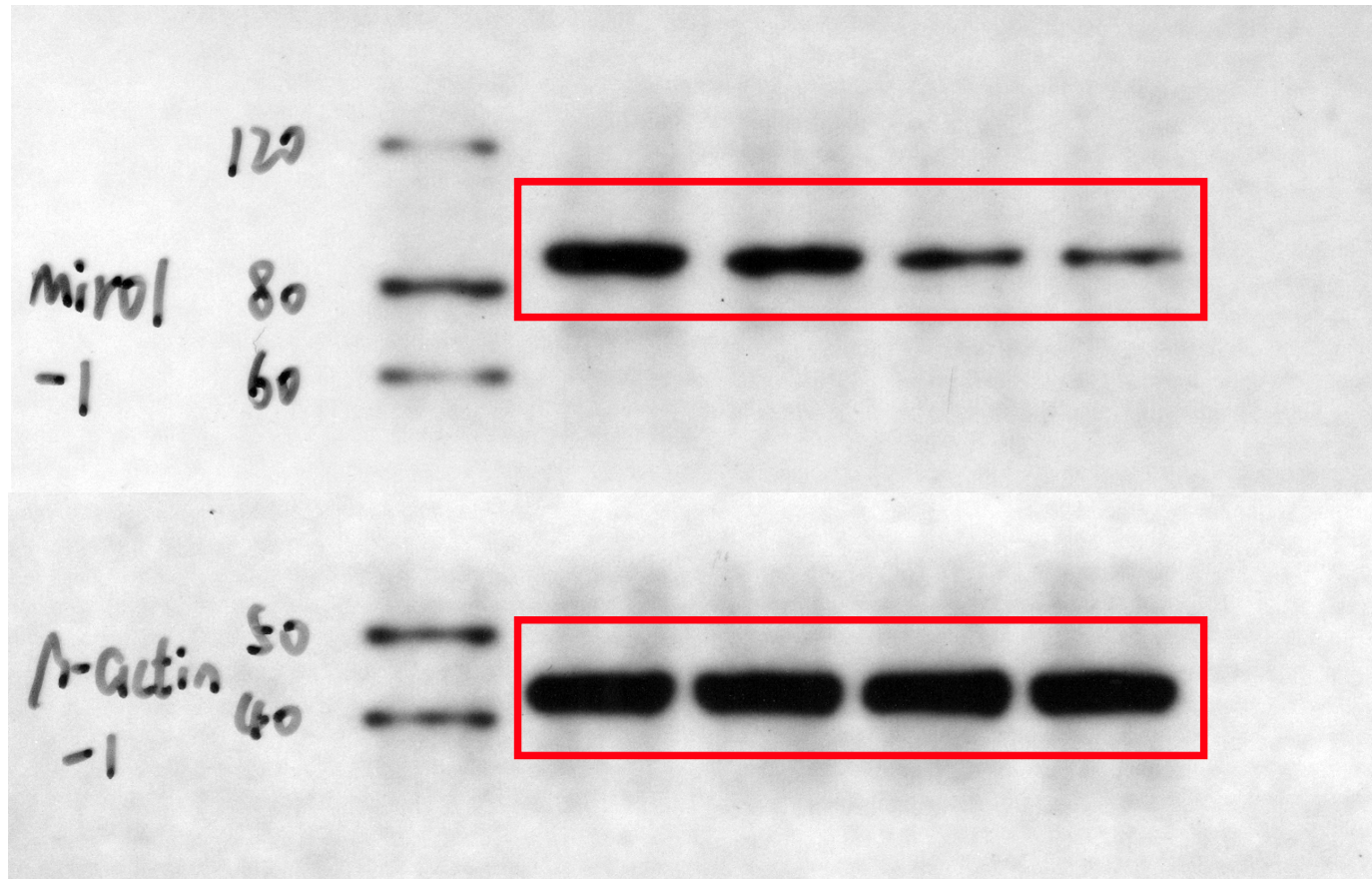

FIG.1D

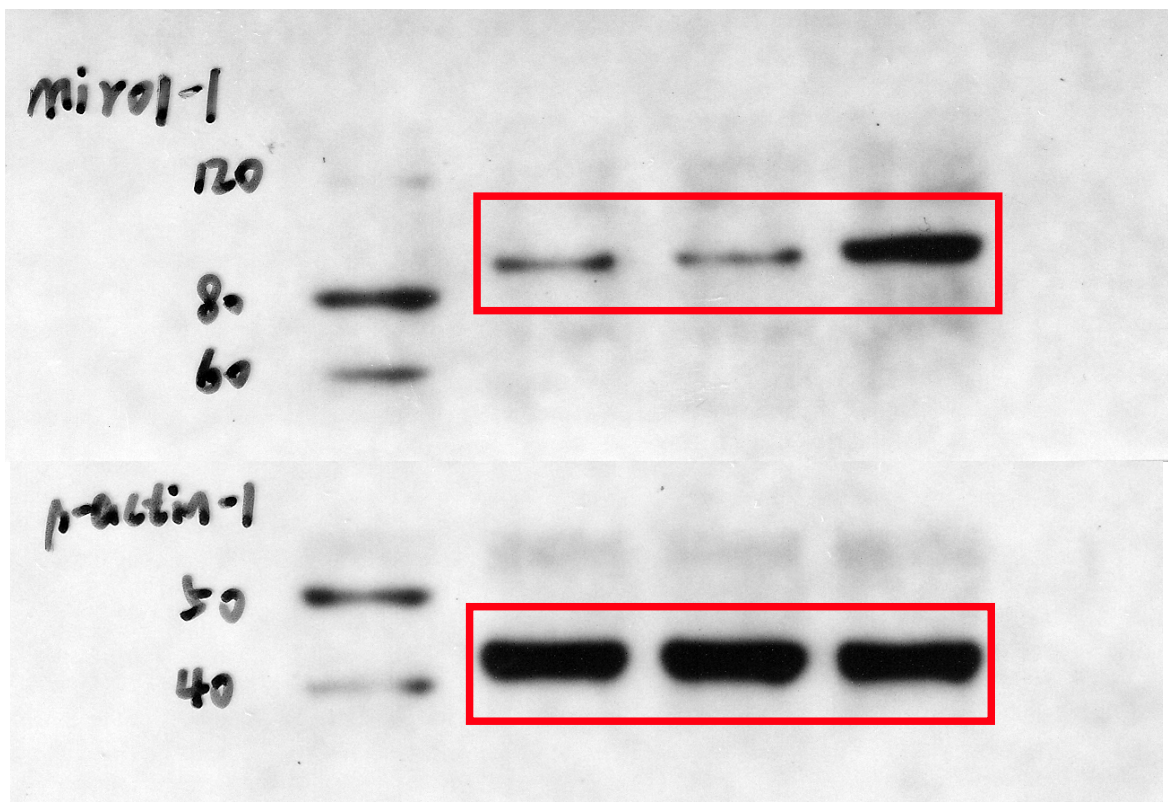

FIG.2A

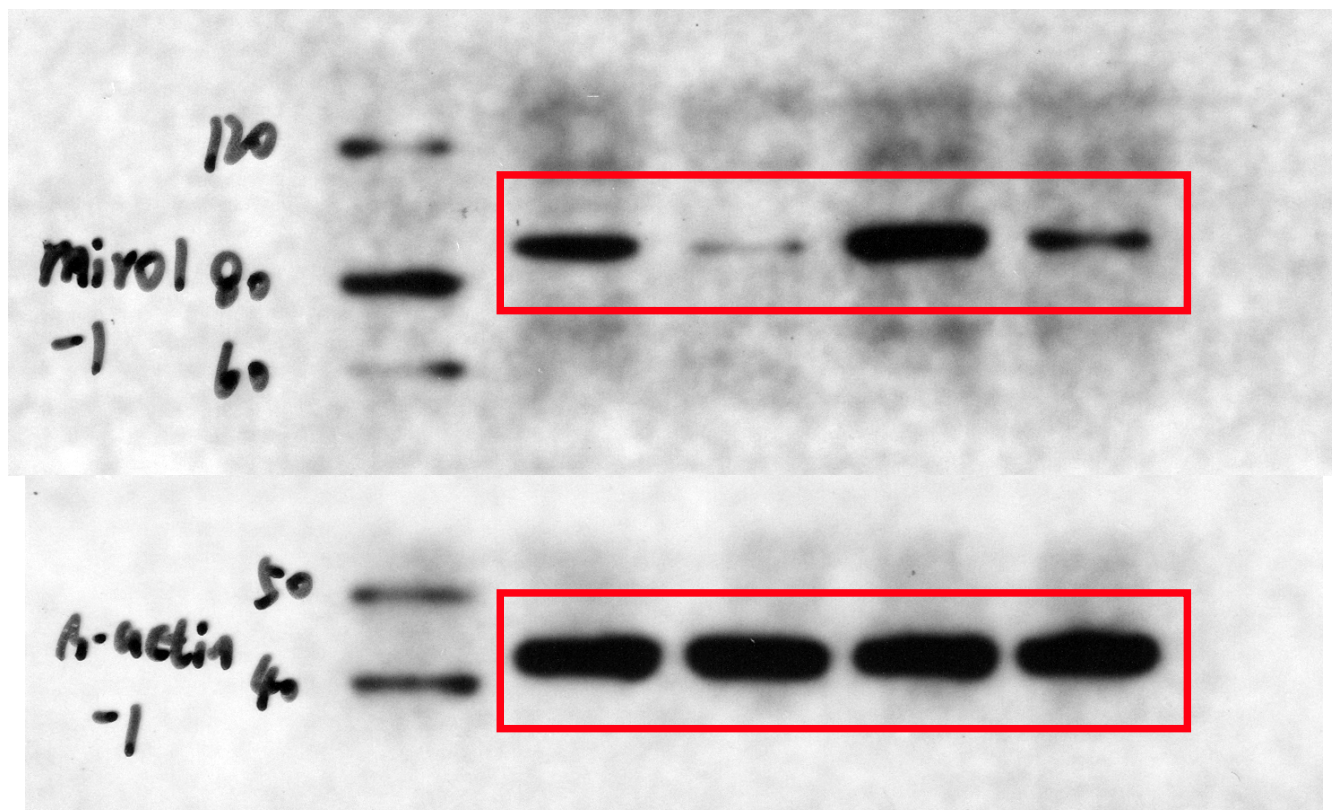

Supplementary FigureS8

FIG.2B

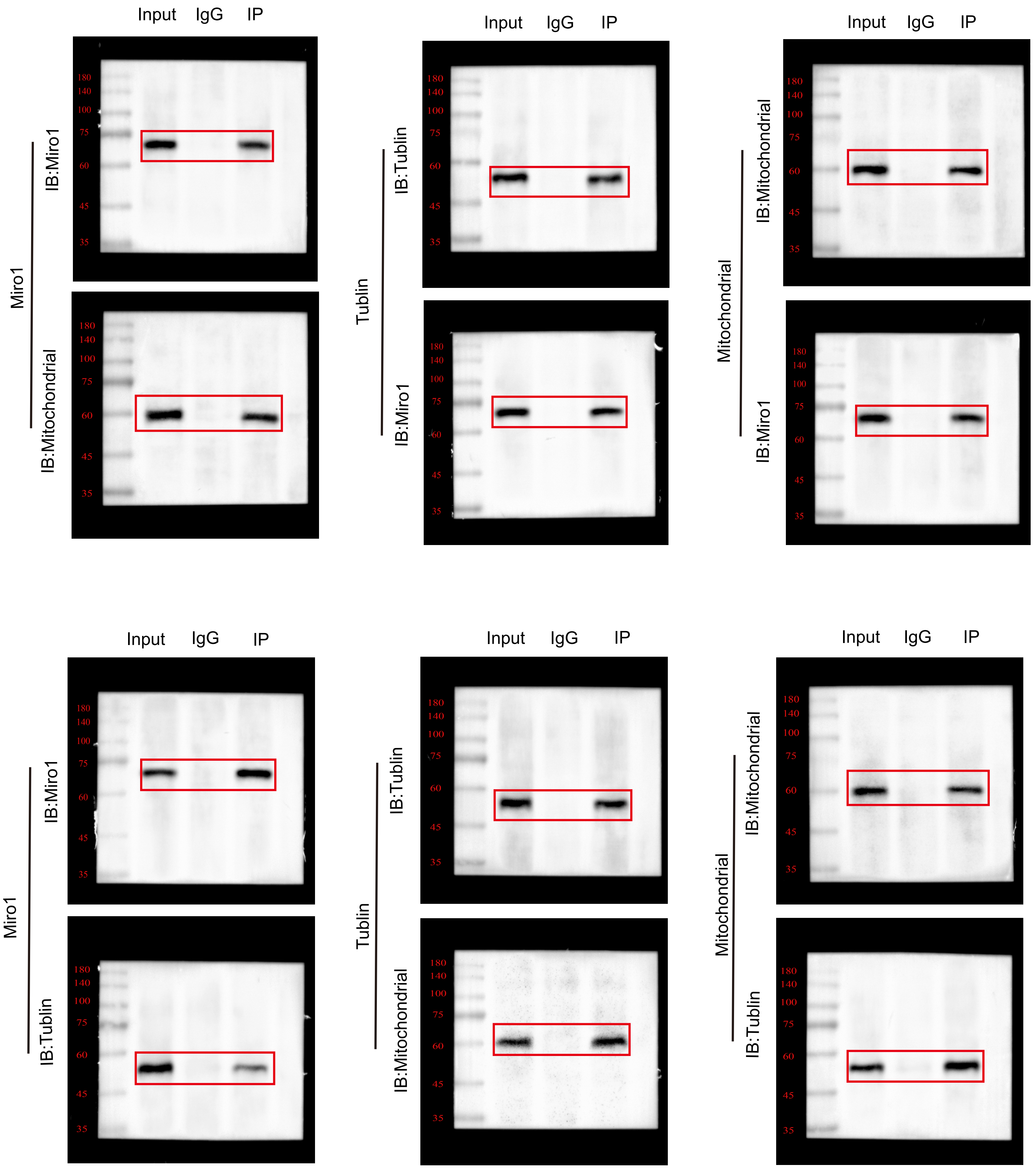

Supplementary FigureS9

FIG.3A

Miro1

B-actin

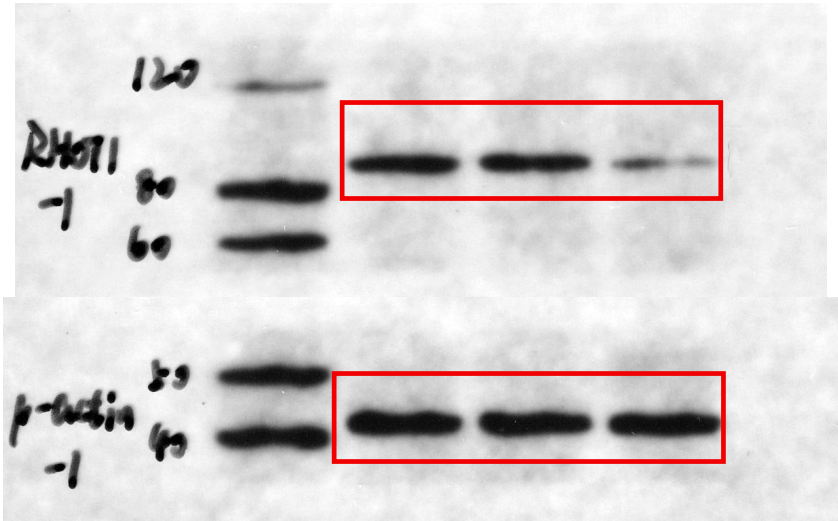

FIG.4E
&SuplementaryFIG.2C

Atrogin1

MuRF1

GAPDH

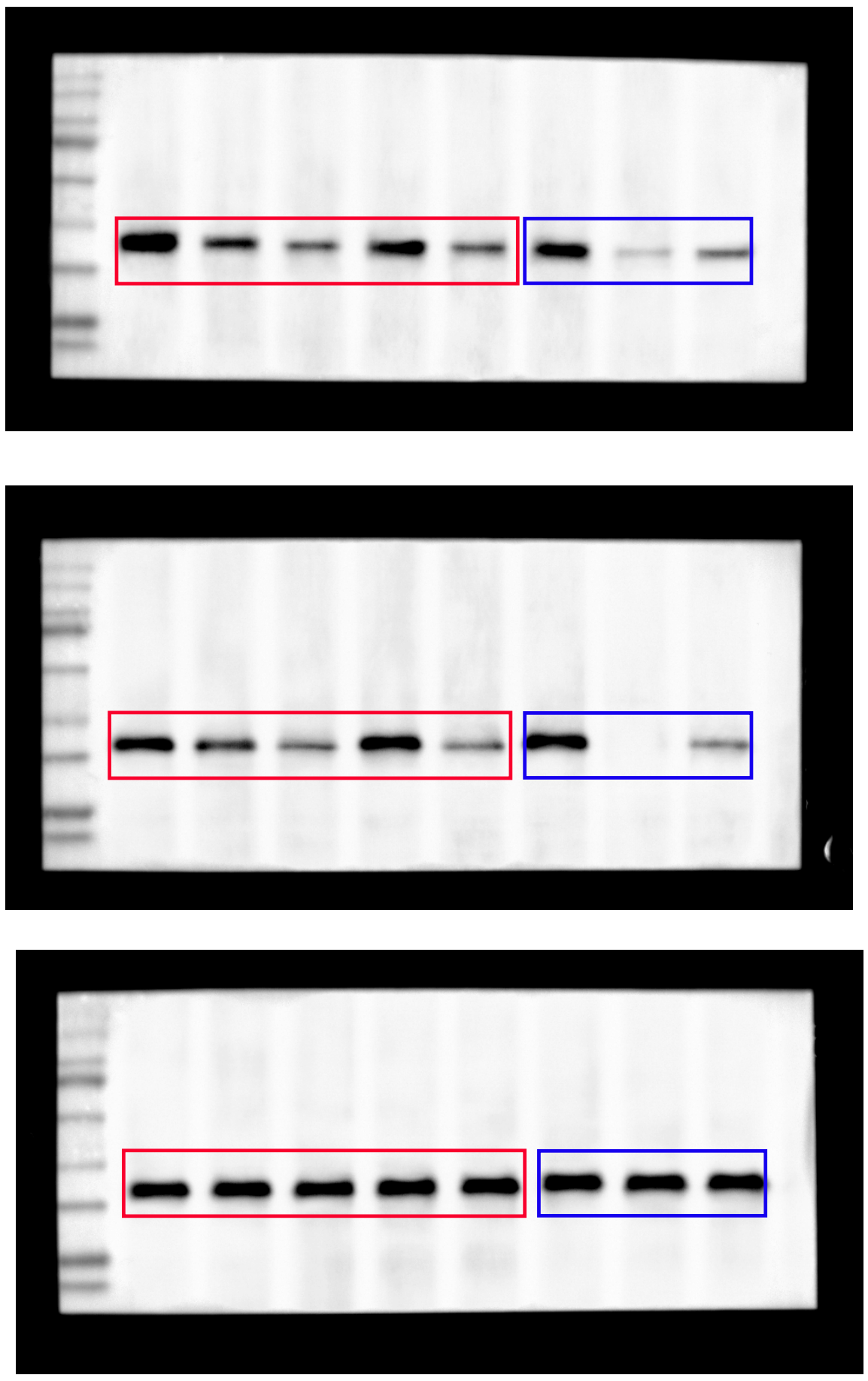

FIG.8A

Miro1

B-actin

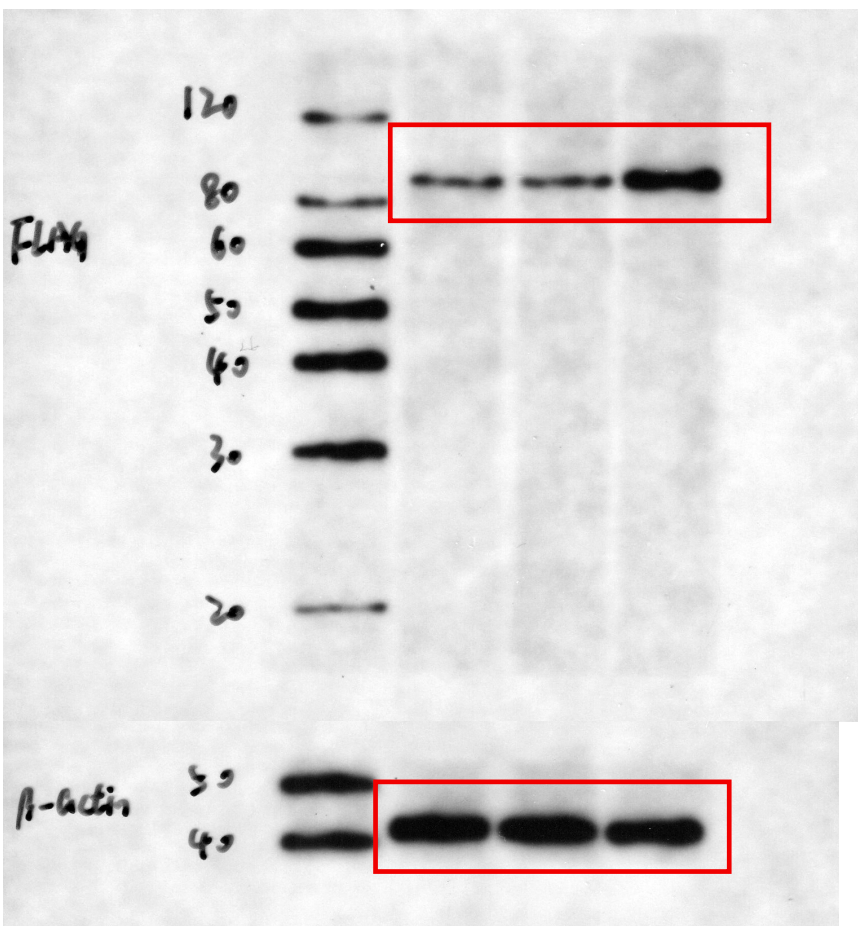

Suplementary FIG.2D

Miro1

Miro1

GAPDH

GAPDH

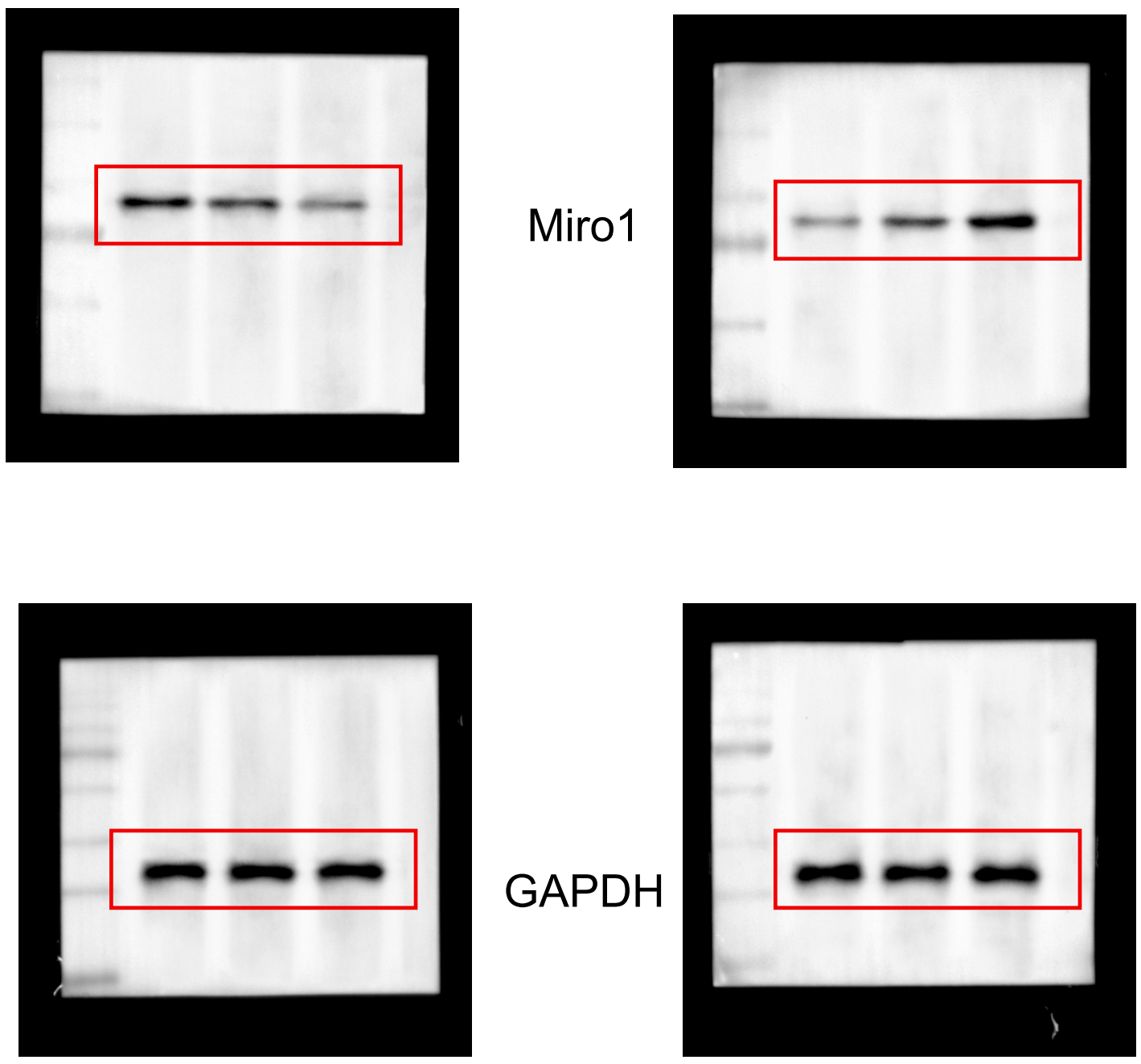

Supplementary FigureS10

FIG.7B & Suplementary FIG.4A

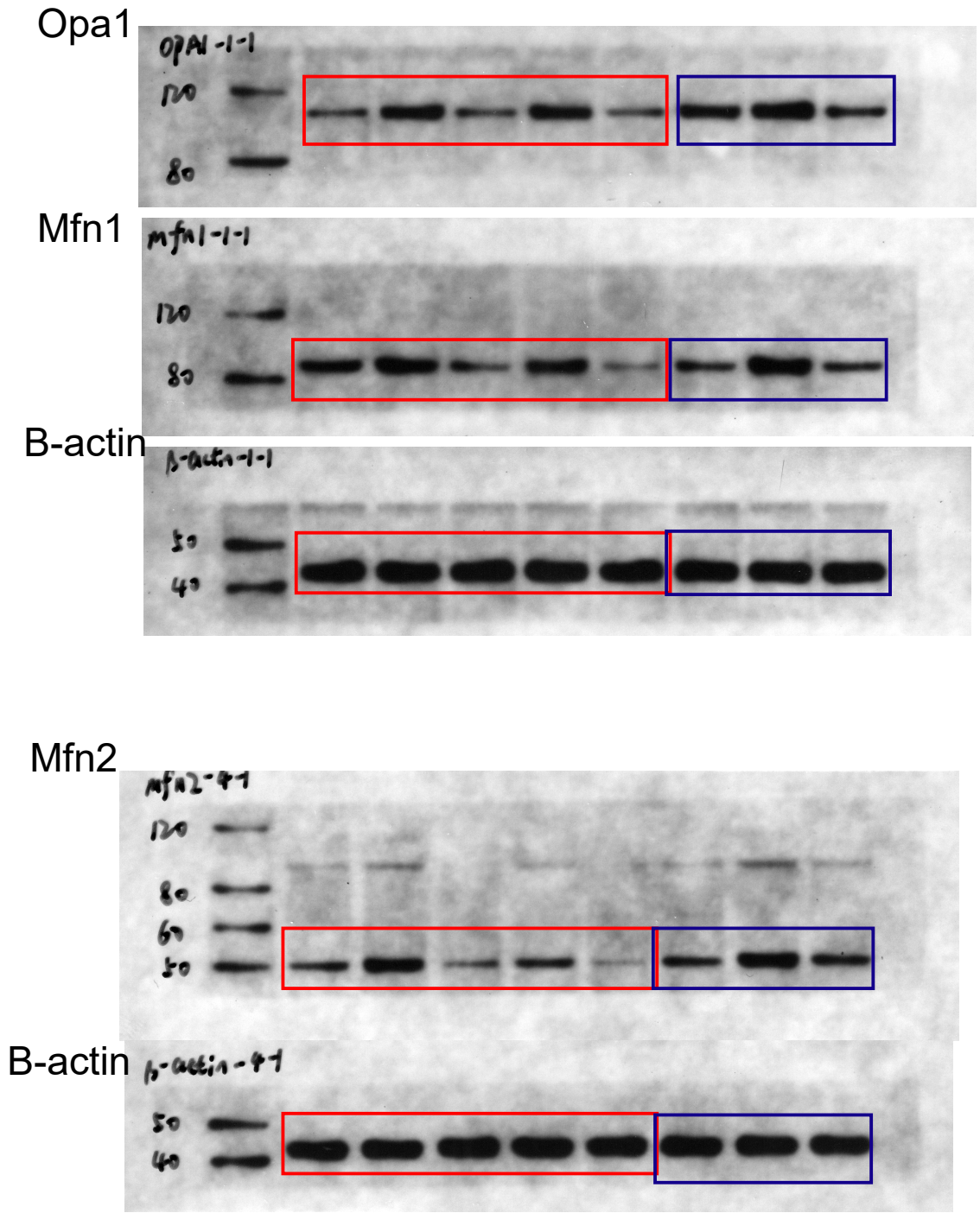

FIG.7D & Suplementary FIG.4B

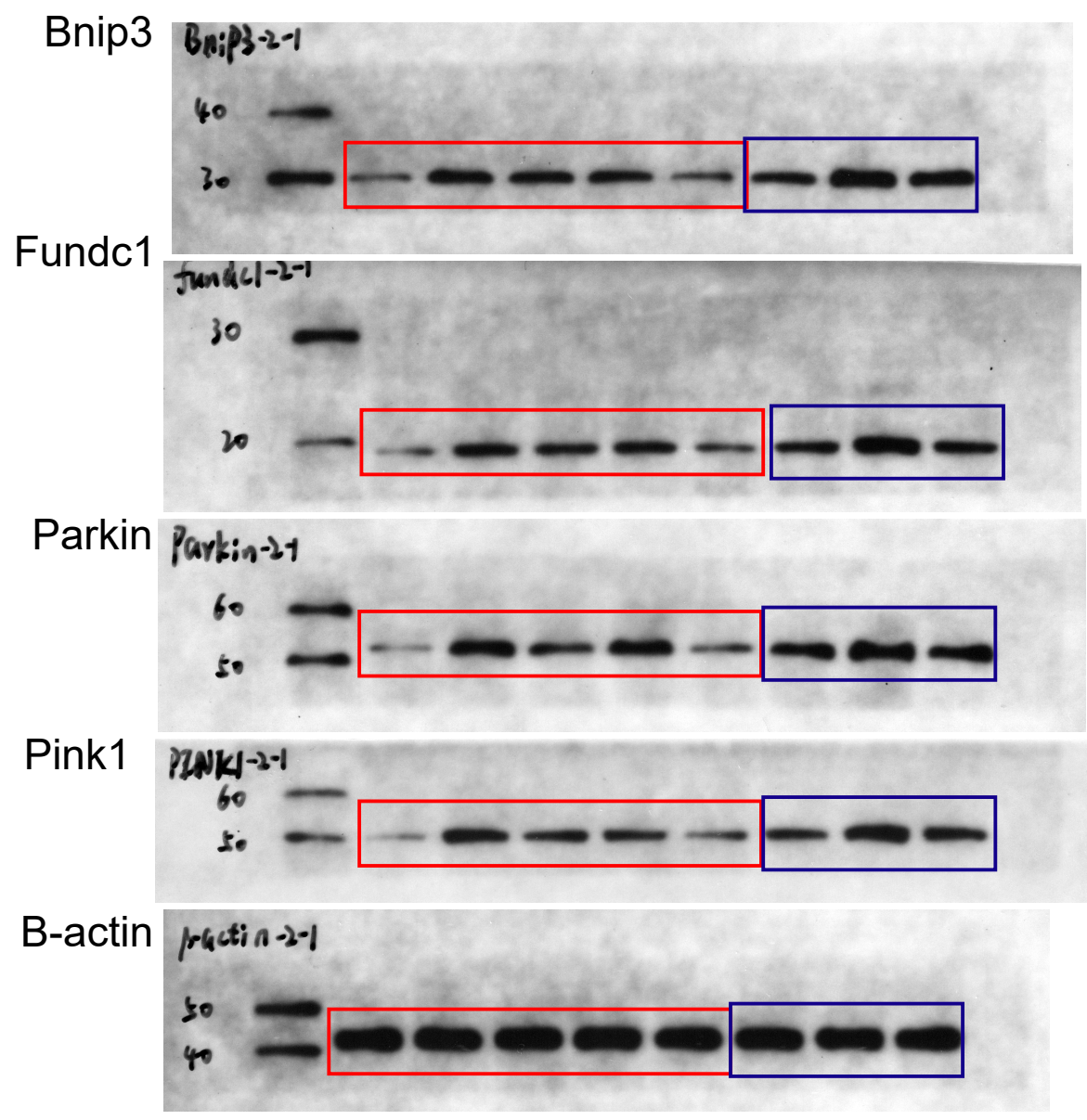

FIG.7E & Suplementary FIG.4C

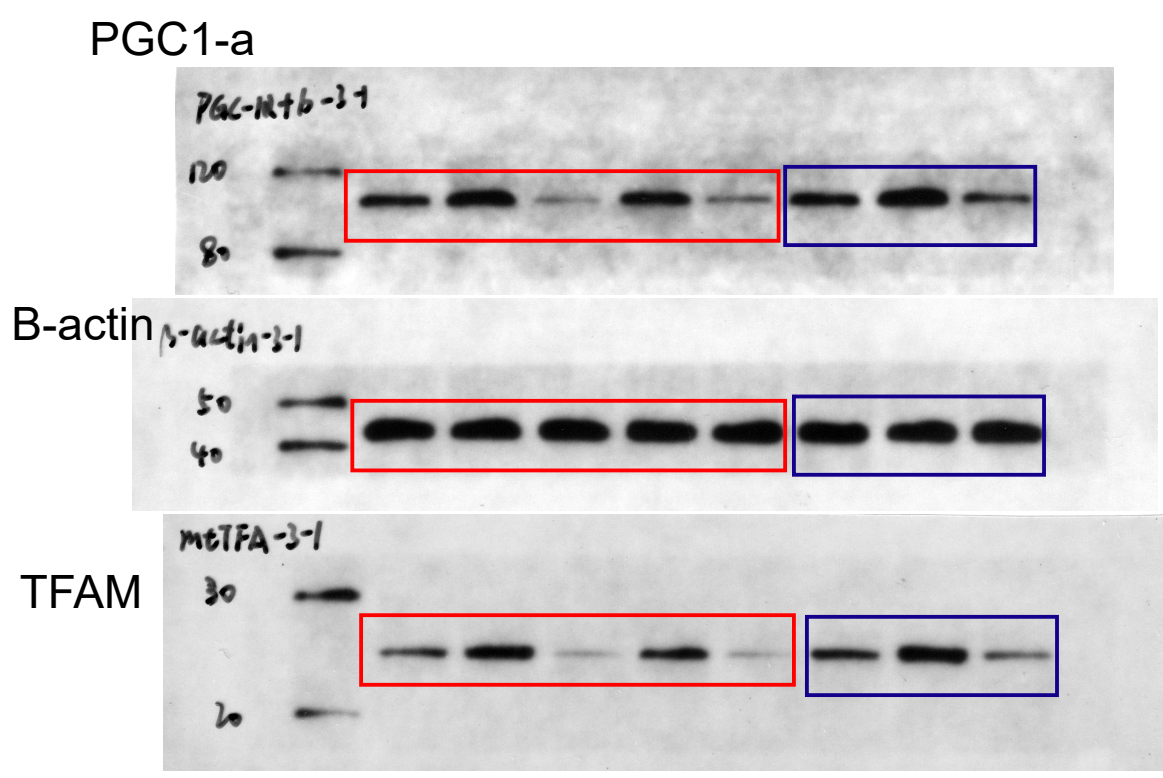

Supplementary FigureS11

Supplementary FIG. 1& SupplementrayFIG. 5

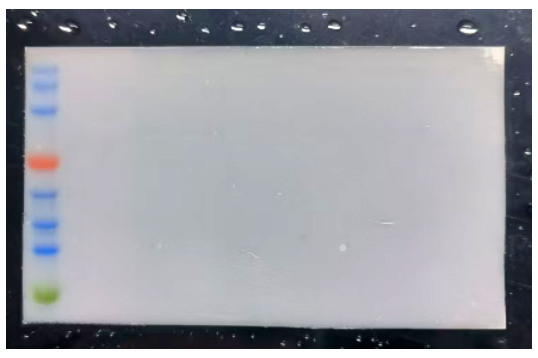

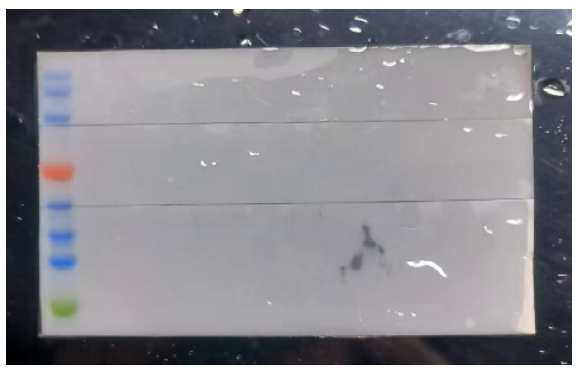

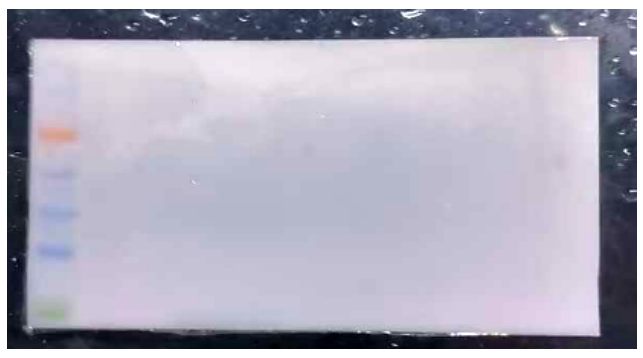

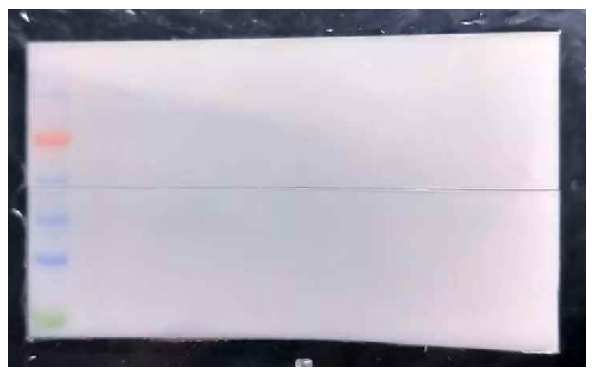

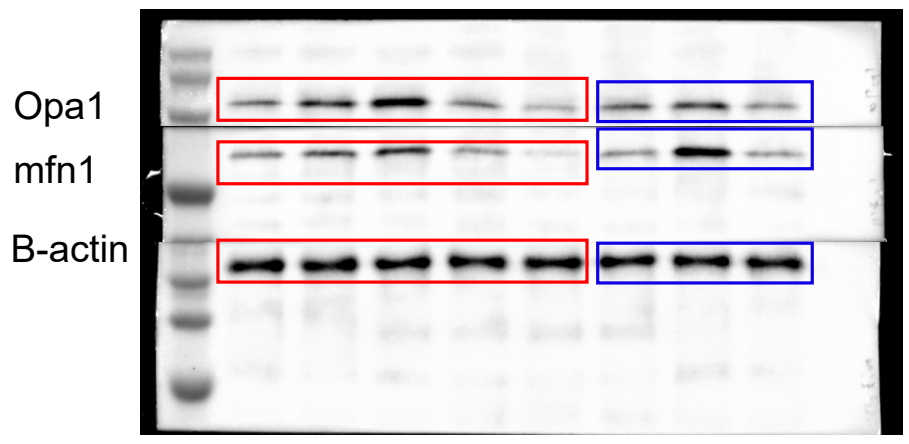

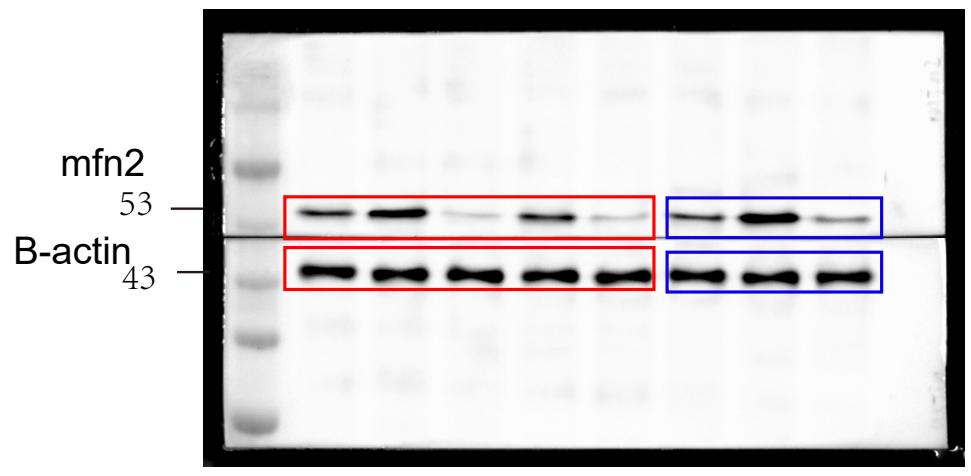

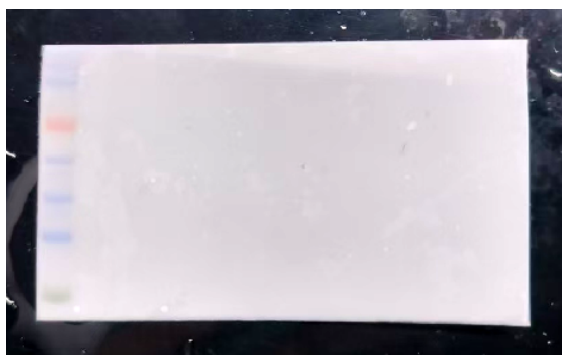

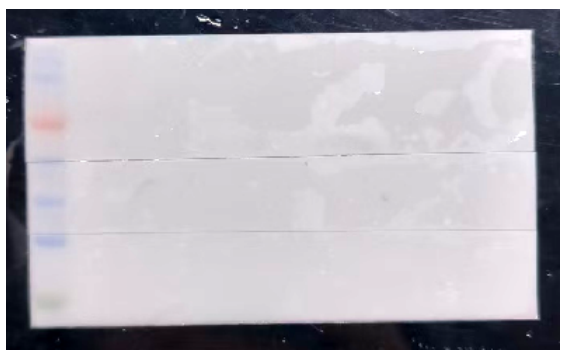

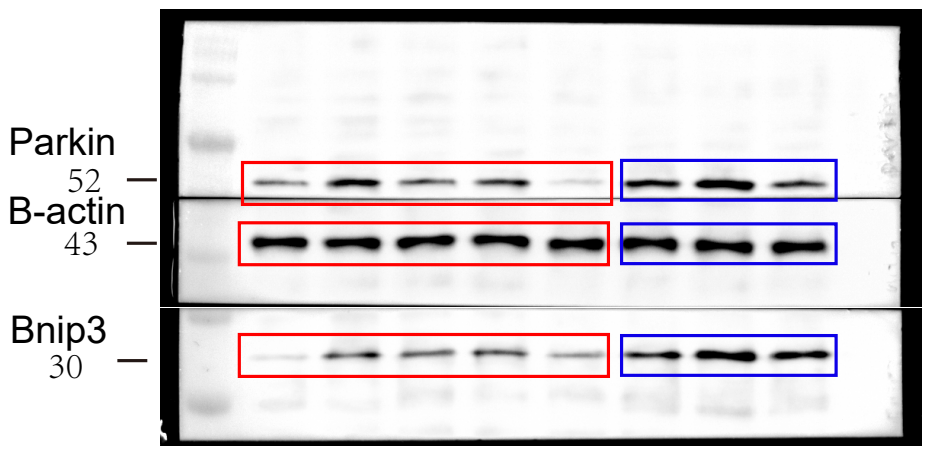

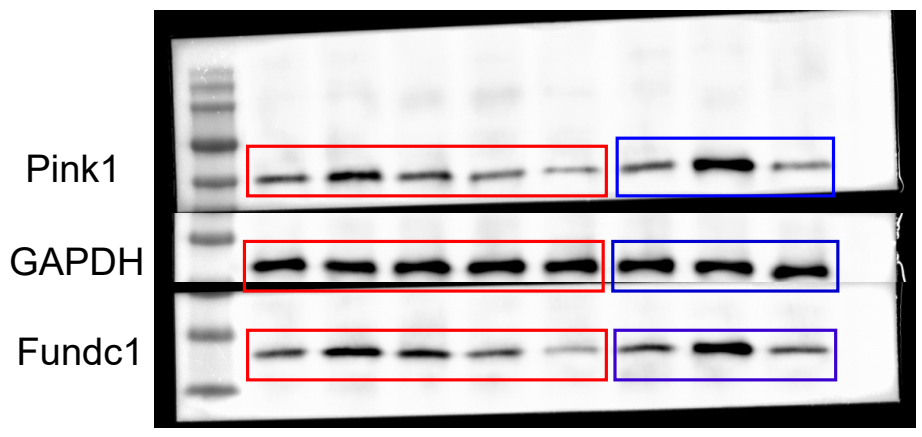

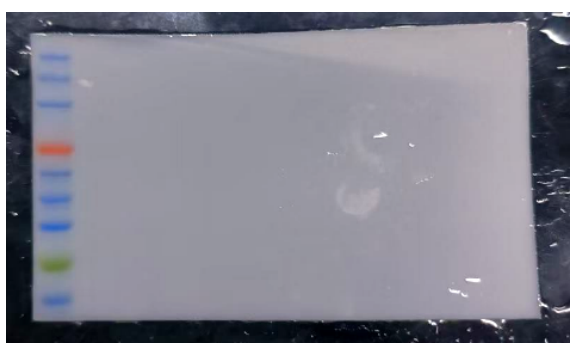

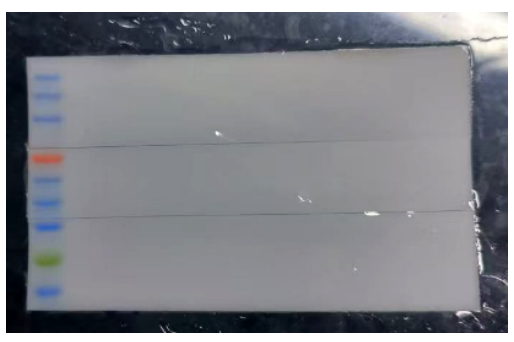

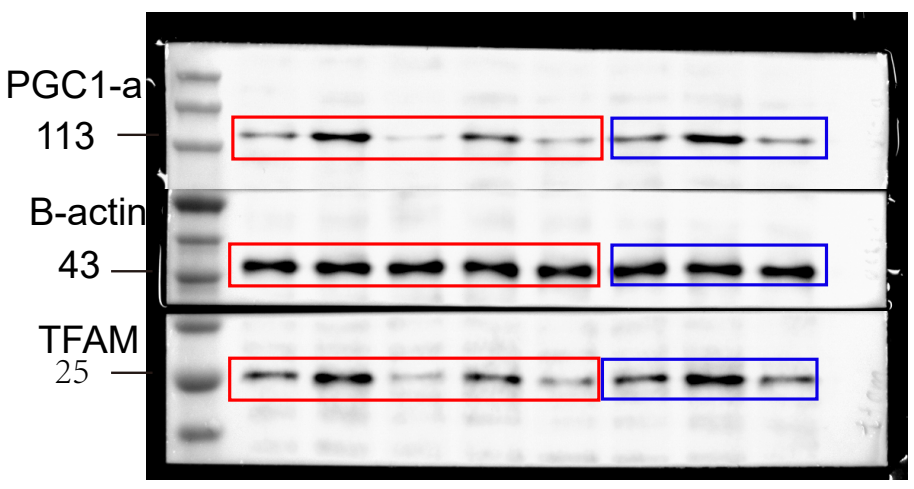

浙江大学医学院附属第二医院动物伦理委员会批件

批件号：（2024 年度）第（230）号

<table>
<tr><td>项目名称</td><td colspan="3">隧道纳米管在干细胞治疗肌少症中的作用和分子机制研究</td></tr>
<tr><td>承担科室</td><td>神经内科</td><td>主要研究者 PI</td><td>刘恺鸣</td></tr>
<tr><td>审查形式</td><td colspan="3">口会议审查　　■快速审查</td></tr>
<tr><td colspan="4">审查日期：2024 年 10 月 25 日　　会议地点：无</td></tr>
<tr><td>审查委员</td><td colspan="3">朱永良 鲁珽</td></tr>
<tr><td>有效期</td><td colspan="3">2024 年 10 月 25 日 至 2025 年 10 月 25 日</td></tr>
<tr><td>审查频率</td><td colspan="3">口 6 个月　■12 个月　口其他________</td></tr>
<tr><td colspan="4">主任/副主任委员签名：　　日期：2024 年 10 月 25 日
浙江大学医学院附属第二医院实验动物伦理委员会（盖章）</td></tr>
<tr><td>备注</td><td colspan="3">1. 研究应在批准之日起 1 年内实施，逾期未实施，本批件自行废止。
2. 严格遵从批准的方案实施，研究过程中对方案有任何修改，请提交修改申请，得到伦理委员会批准后方可继续实施。
3. 及时提交年度审查报告。
4. 方案违背、提前终止均应提供书面报告。
5. 研究结束提供总结报告。</td></tr>
</table>

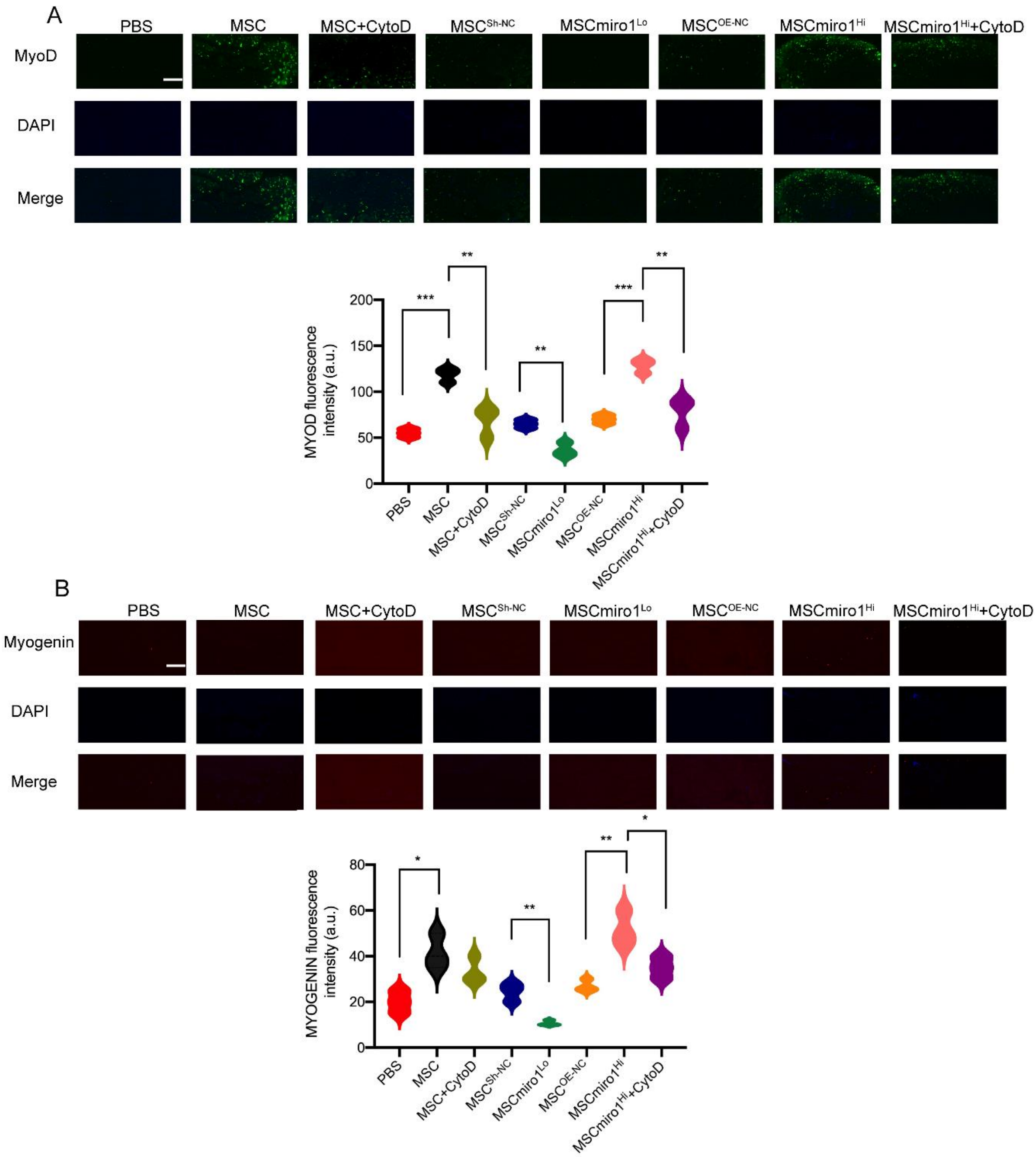

Supplementary Figure S12. Miro1 overexpression upregulates the myogenesis-related markers MyoD and Myogenin. gastrocnemius muscle of muscle atrophy mice treated with PBS, MSC, MSC+CytoD, MSC^{Sh-NC}, $MSCmiro1^{Lo}$, MSC^{OE-NC}, $MSCmiro1^{Hi}$ and $MSCmiro1^{Hi}$ +CytoD, respectively. (PBS compared with MSC, MSC compared with MSC+CytoD, MSC^{Sh-NC} compared with $MSCmiro1^{Lo}$, MSC^{OE-NC} compared with $MSCmiro1^{Hi}$; $MSCmiro1^{Hi}$ compared with $MSCmiro1^{Hi}$ + CytoD). **(A)** Immunofluorescence staining of MyoD. **(B)** Immunofluorescence staining of Myogenin. Data are means ± SD, n = 3. (**P* < 0.05, ***P* < 0.01, ****P* < 0.001, *****P* < 0.0001 by two-sided unpaired Student's t-test; nonsignificant differences not shown).

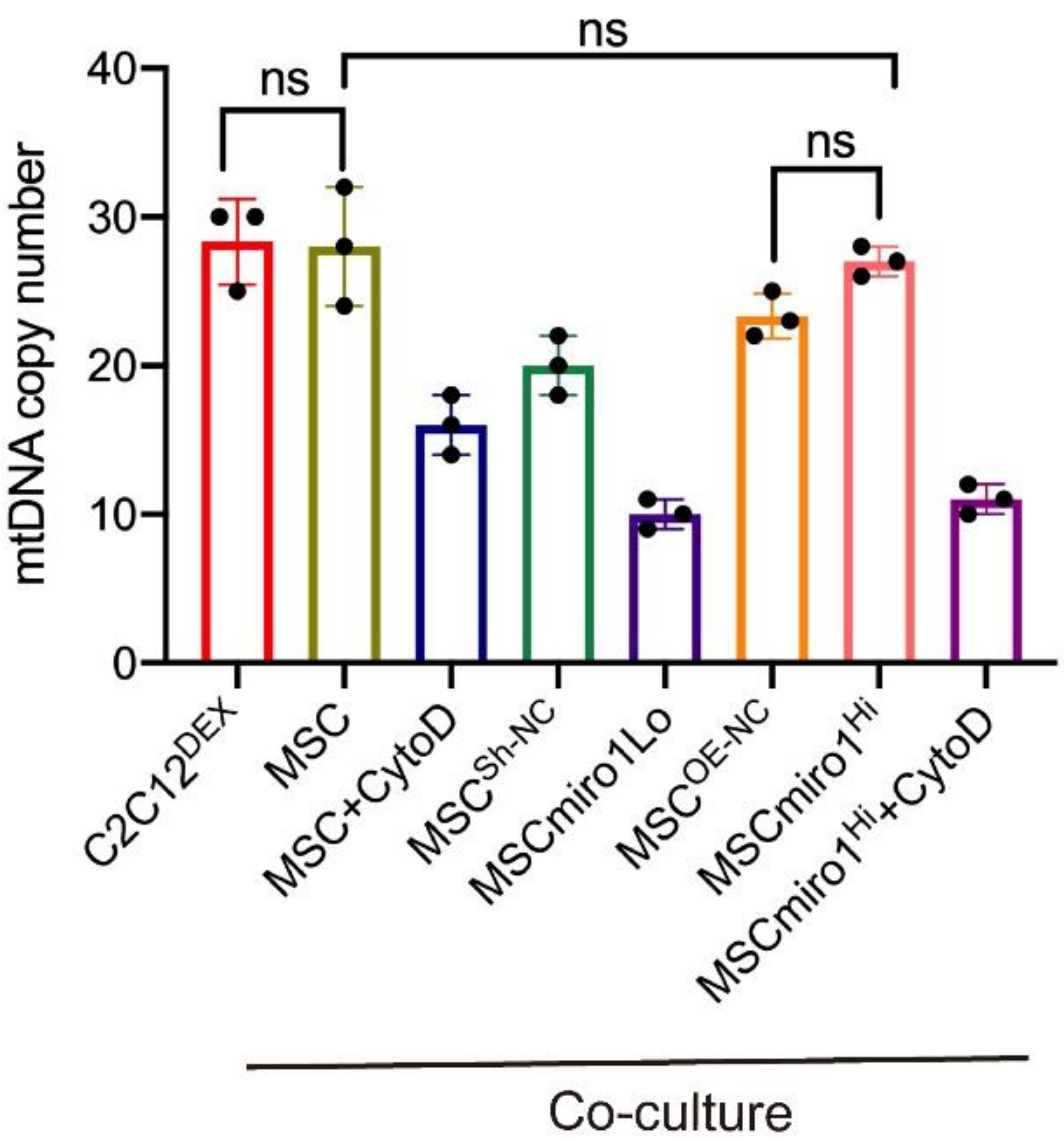

Supplementary Figure S13. QPCR analysis of the mtDNA/nDNA ratio revealed no significant difference in mitochondrial copy number between the MSC co-culture group and the model group.

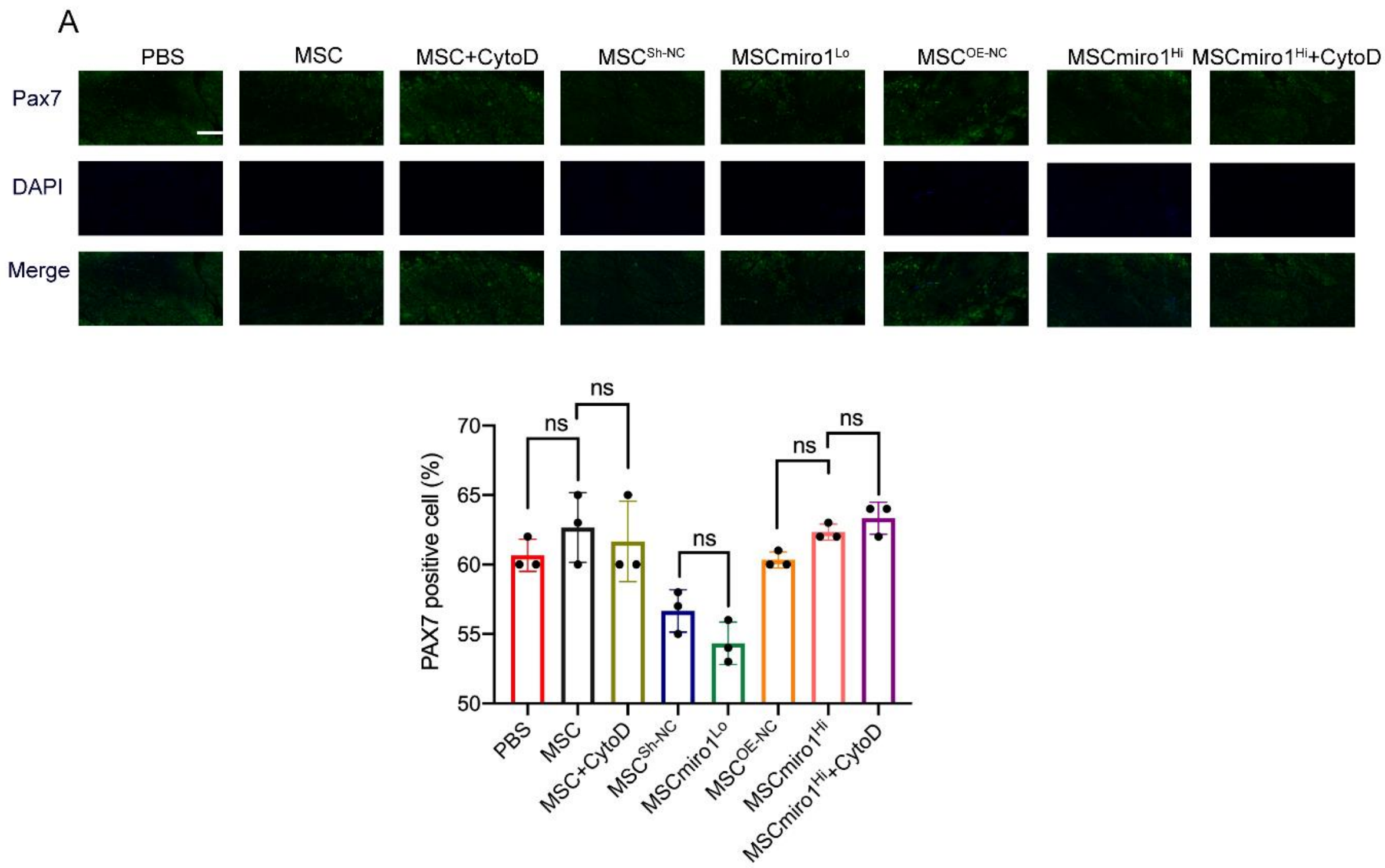

Supplementary Figure S14. Immunofluorescence staining was performed to detect the satellite cell-specific marker Pax7. The results showed that the number of Pax7-positive cells exhibited no significant difference between the MSC treatment group and the model group, confirming that the improvement of muscle atrophy mediated by mitochondrial transfer was not achieved through satellite cell activation and new myonucleus formation.

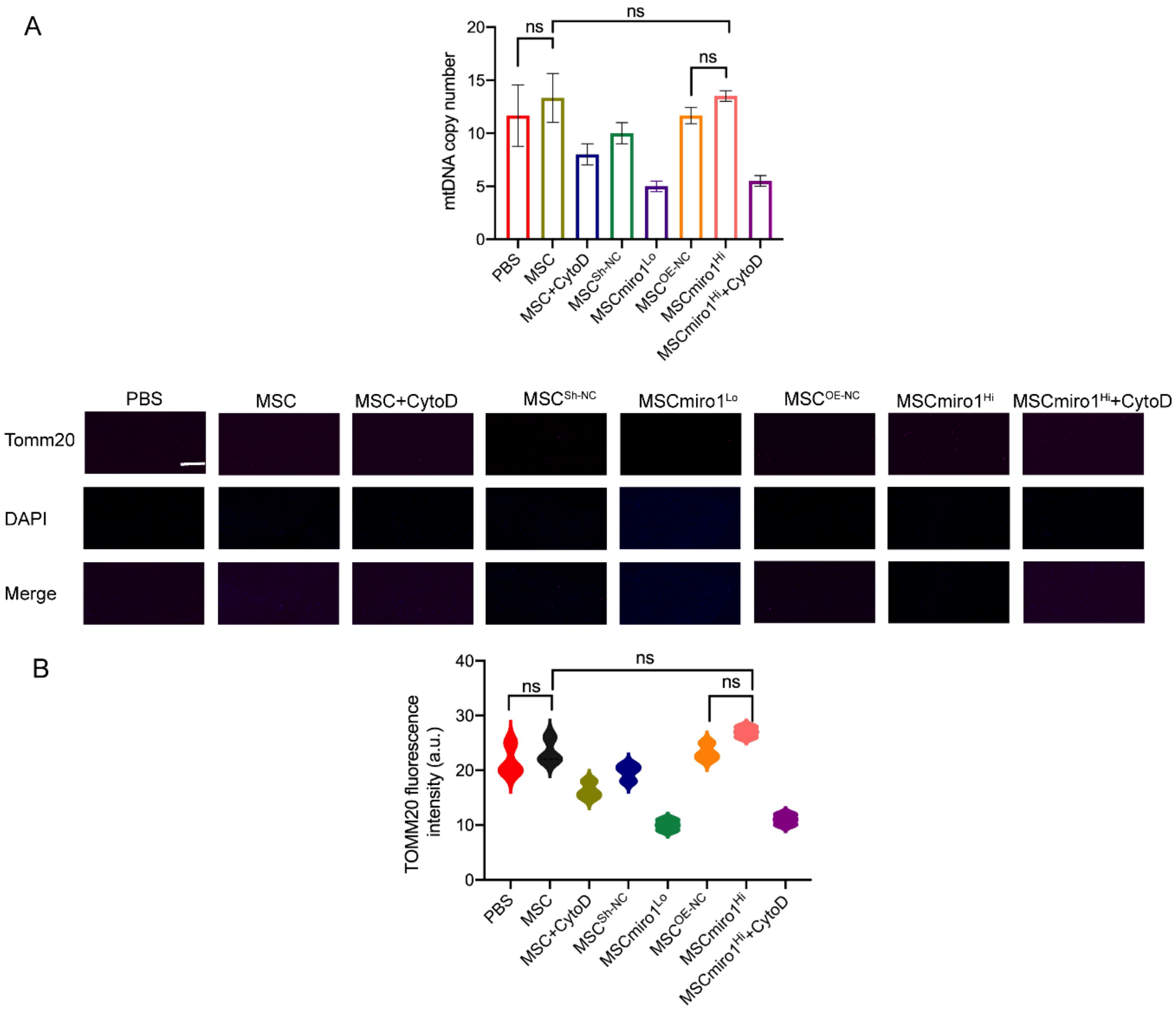

Supplementary Figure S15. QPCR analysis of the mtDNA and Immunofluorescence quantification were performed using the mitochondrial specific marker Tomm20. The results showed that there was no significant difference in the total mitochondrial content between the Miro1-overexpressing MSC treatment group and the control group, which ruled out the possibility that the improvement of mitochondrial function was merely attributed to an increase in mitochondrial mass.

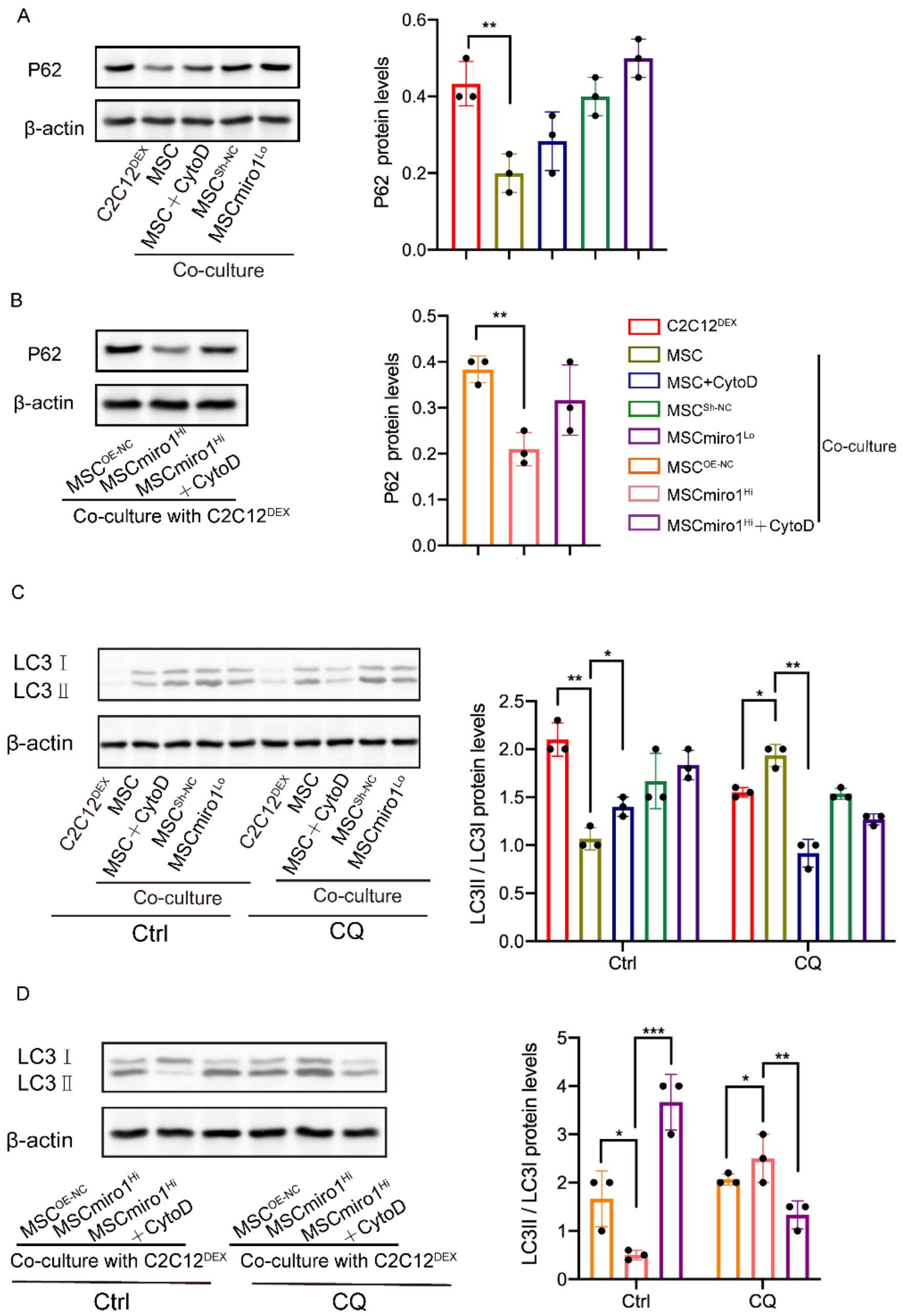

Supplementary Figure S16.
Western blot was used to detect the expression of autophagic substrate p62, and the LC3-II/LC3-I ratio was measured to evaluate autophagic flux in untreated C2C12^{DEX} or those sorted after co-culture with MSC, MSC + CytoD, MSC^{sh-NC}, MSCmiro1^{Lo}, MSC^{OE-NC}, MSCmiro1^{Hi}, and MSCmiro1^{Hi}+CytoD respectively. (Untreated C2C12^{DEX} compared with those after MSC co-culture; C2C12^{DEX} after MSC co-culture compared with those after MSC + CytoD co-culture; C2C12^{DEX} after MSC^{sh-NC} co-culture compared with those after MSCmiro1^{Lo} co-culture; C2C12^{DEX} after MSC^{OE-NC} co-culture compared with those after MSCmiro1^{Hi} co-culture; C2C12^{DEX} after MSCmiro1^{Hi} co-culture compared with those after MSCmiro1^{Hi}+CytoD co-culture) Data are means ± SD, n = 3. (*P < 0.05, **P < 0.01, ***P < 0.001, ****P < 0.0001 by two-sided unpaired Student's t-test; nonsignificant differences not shown).

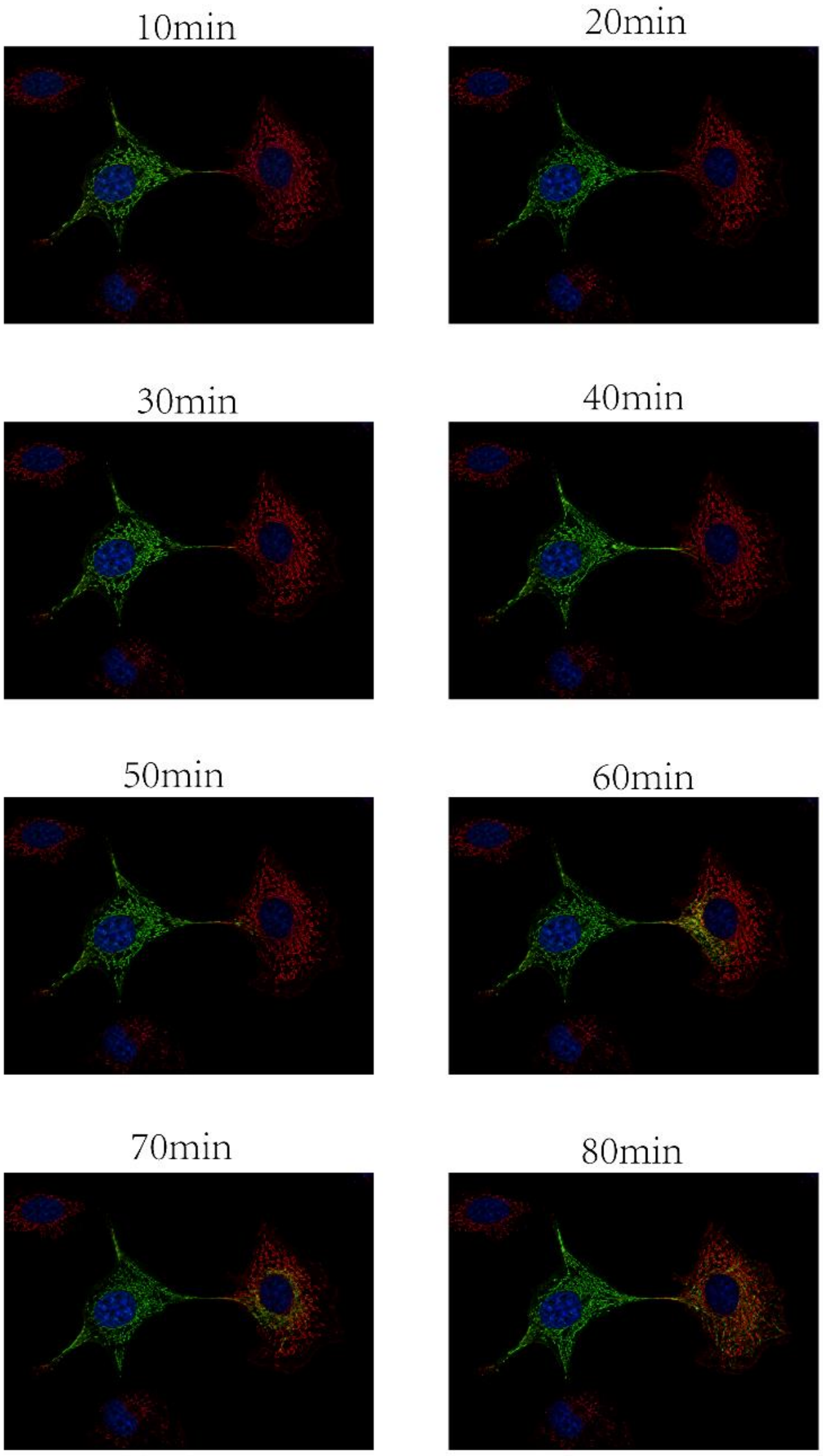

gif.gif

Supplementary Figure S17. **live-cell time-lapse confocal imaging** which clearly records the dynamic process of Mito-EGFP-labeled mitochondria derived from MSCs being transported along TNTs into injured C2C12 myotubes within 10–80 min, directly verifying intercellular mitochondrial transport.

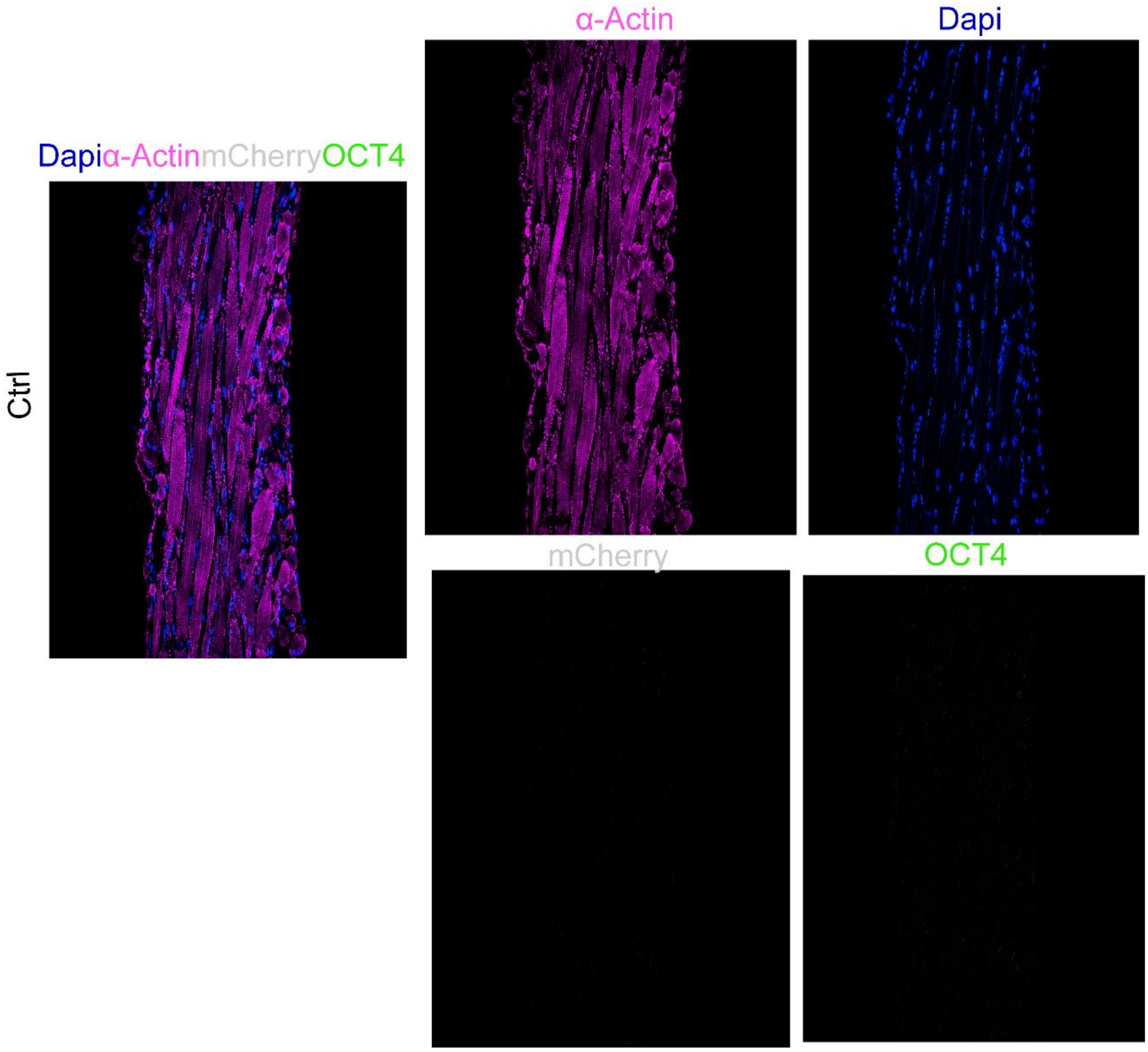

Supplementary Figure S18: a blank control (muscle tissue from mice without MSC injection) and an isotype c ontrol (rabbit IgG was used to replace the primary antibody for OCT4 staining) were established. No specific flu orescent signals were detected in either control group.

Supplementary
Figure S19

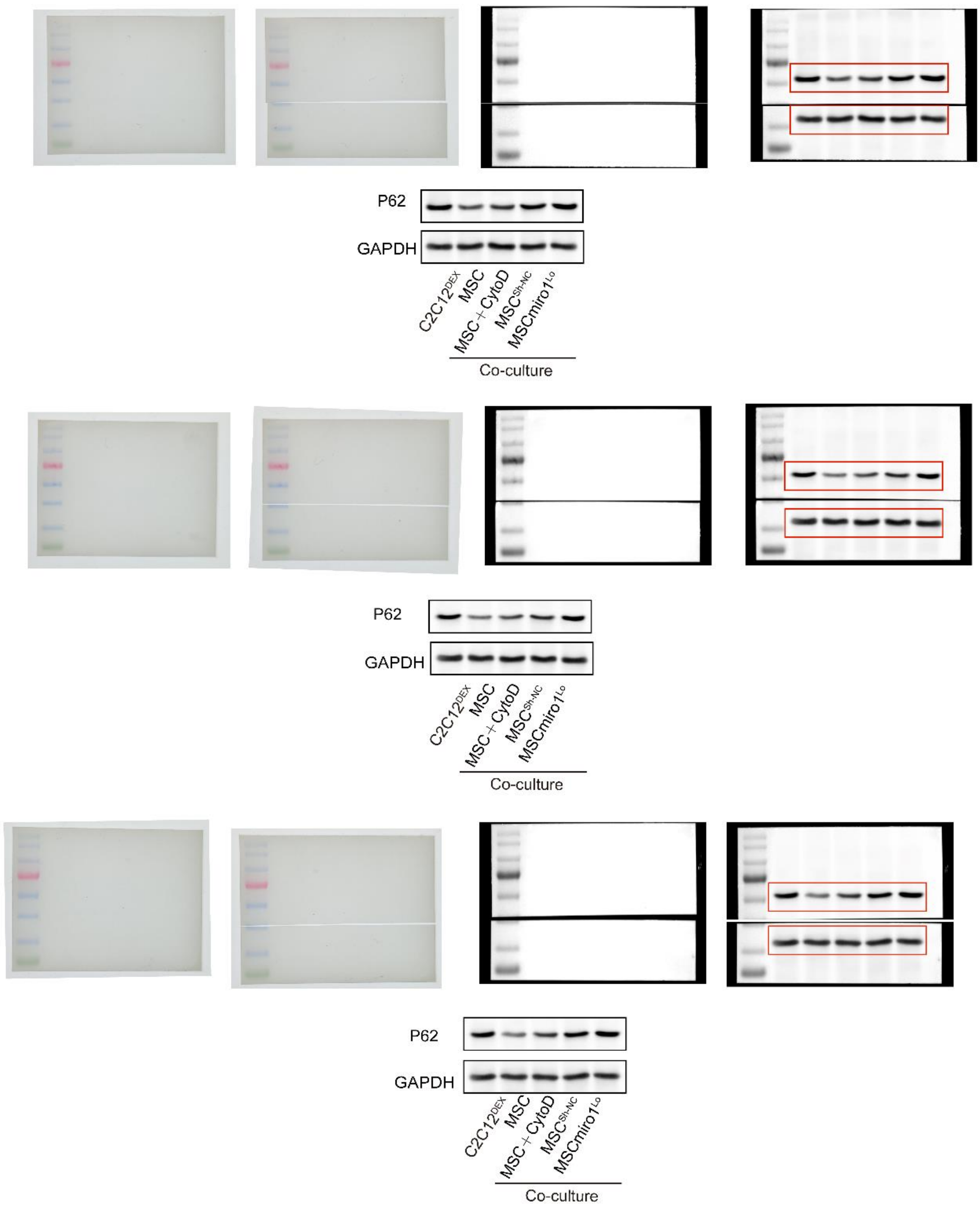

Supplementary
Figure S20

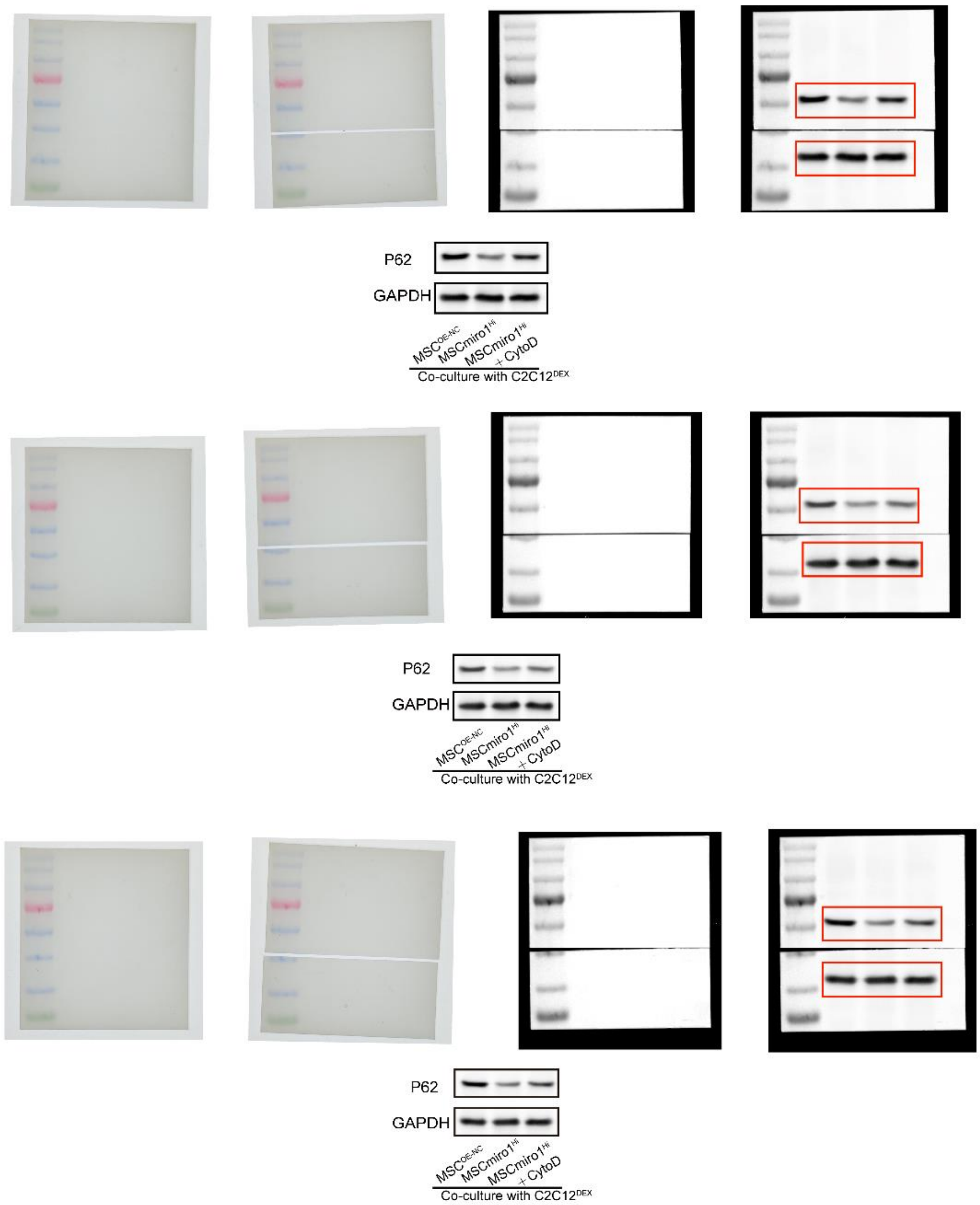

Supplementary
Figure S21

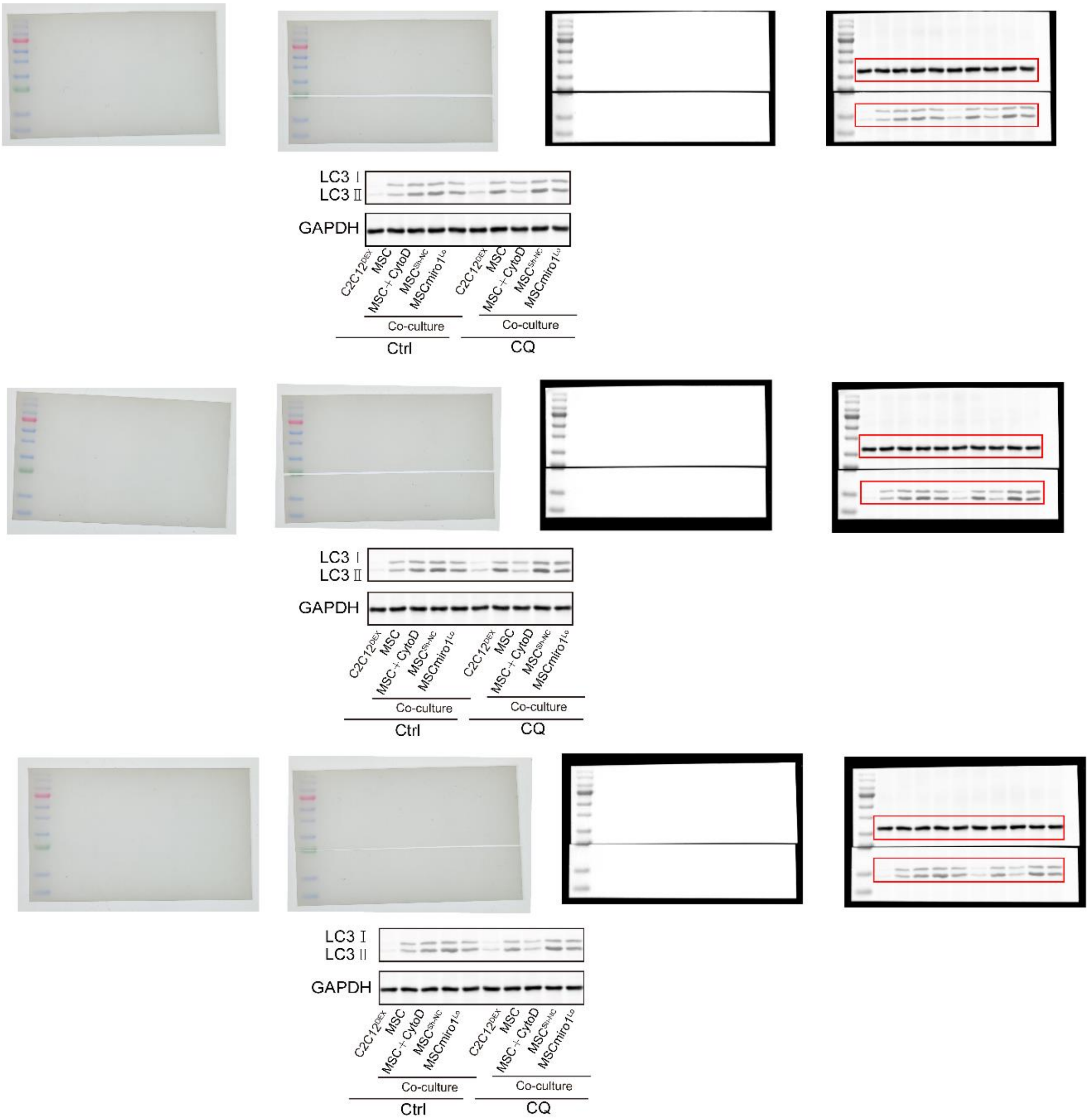

Supplementary
Figure S22

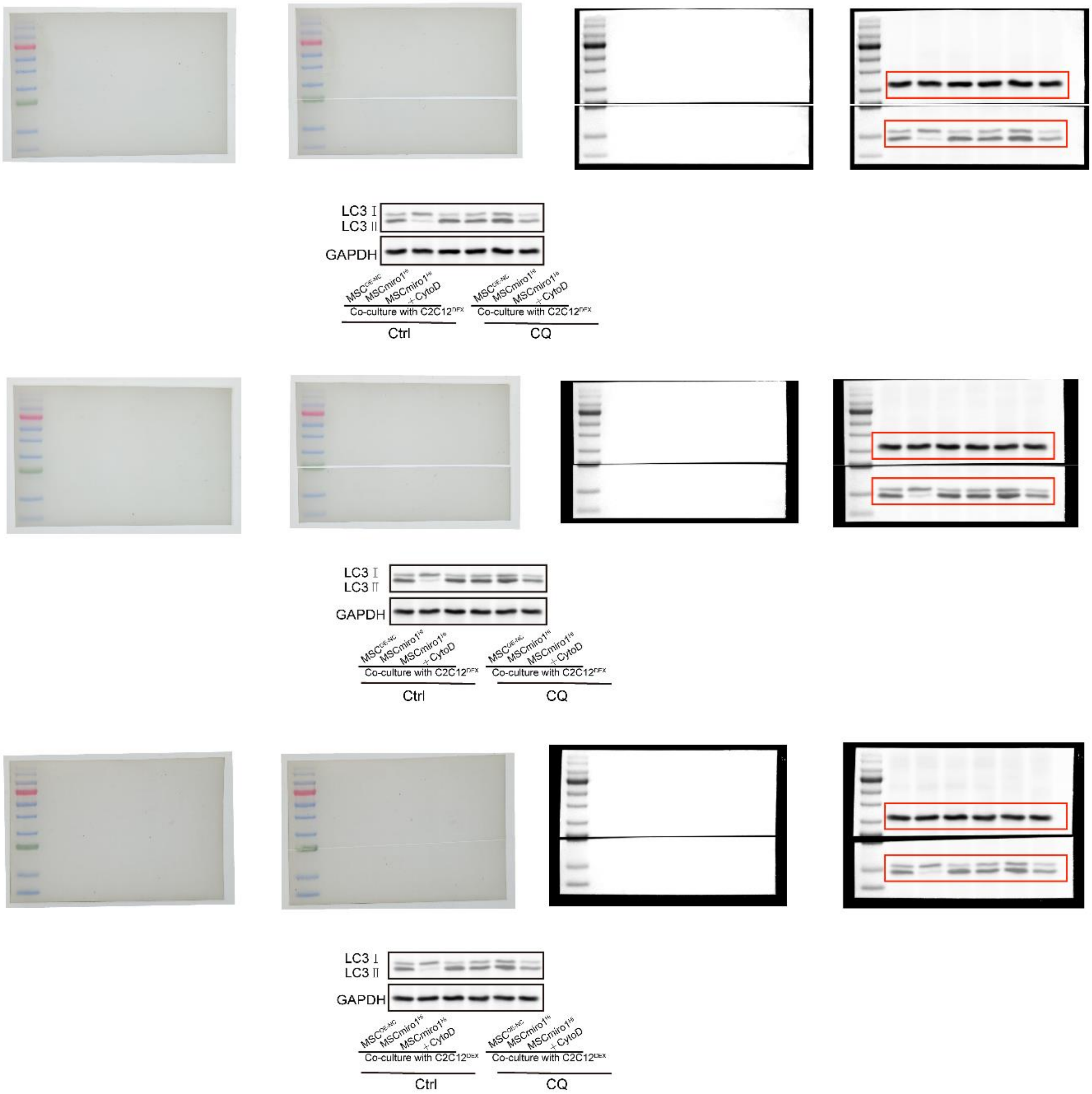

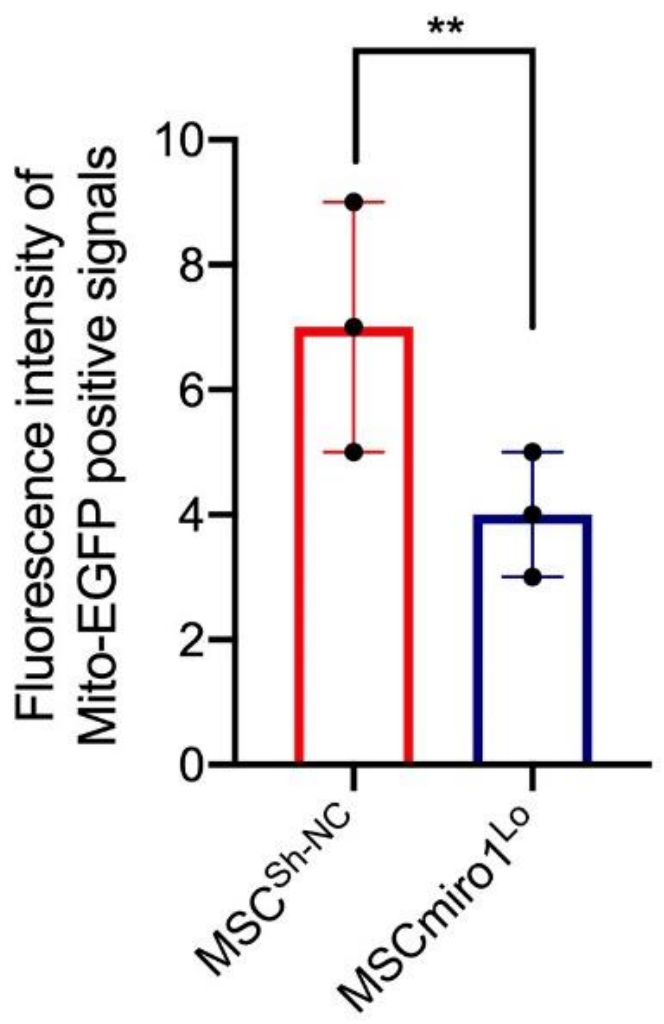

Supplementary Figure 23
The fluorescence intensity of Mito-EGFP-positive signals within TNTs was analyzed using ImageJ software. The results showed that the fluorescence intensity was significantly lower in the MSCmiro1^{Lo} group than in the MSC^{sh-NC} group (P<0.01), providing quantitative support for the conclusion that “mitochondrial signals within TNTs are reduced”.

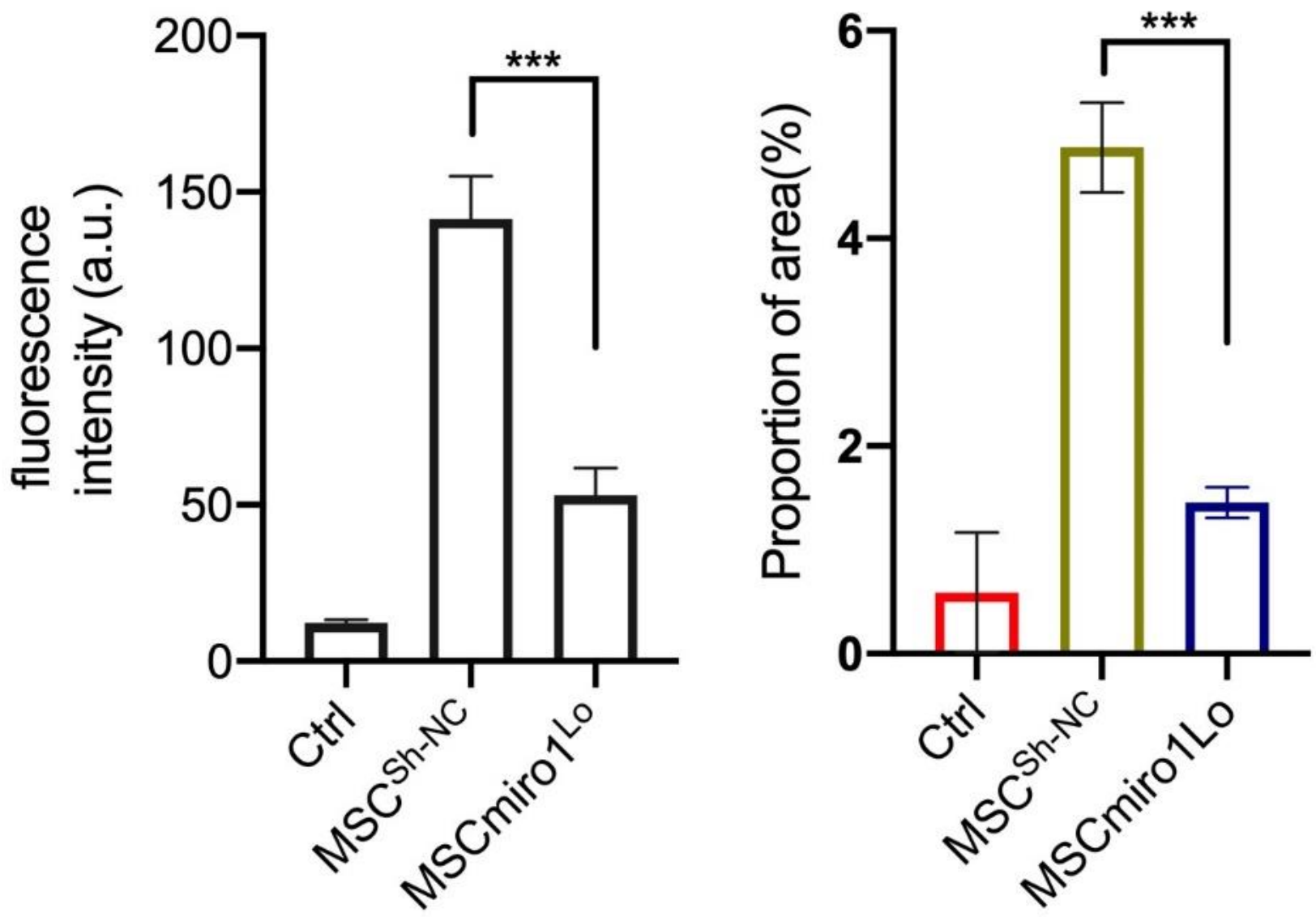

Supplementary Fig. S24. The fluorescence intensity and area percentage of mCherry-positive signals in muscle fibers were analyzed using ImageJ software. The results showed that both the fluorescence intensity and area percentage were significantly lower in the MSCmiro1^{Lo} group than in the MSC^{sh-NC} group (P<0.001), providing quantitative support for this result.
